# Supplementary material for: Kistamicin biosynthesis reveals the biosynthetic requirements for production of highly crosslinked glycopeptide antibiotics
Source: Nat Commun. 2019 Jun 13;10:2613. doi: 10.1038/s41467-019-10384-w (PMC6565677; doi:10.1038/s41467-019-10384-w)
Supplement: Supplementary file 1 — Supplementary Information [file 41467_2019_10384_MOESM1_ESM.pdf]

Supplementary Information

Kistamicin biosynthesis reveals the biosynthetic requirements for production of highly active crosslinked glycopeptide antibiotics

Greule et al.

Supplementary Figures

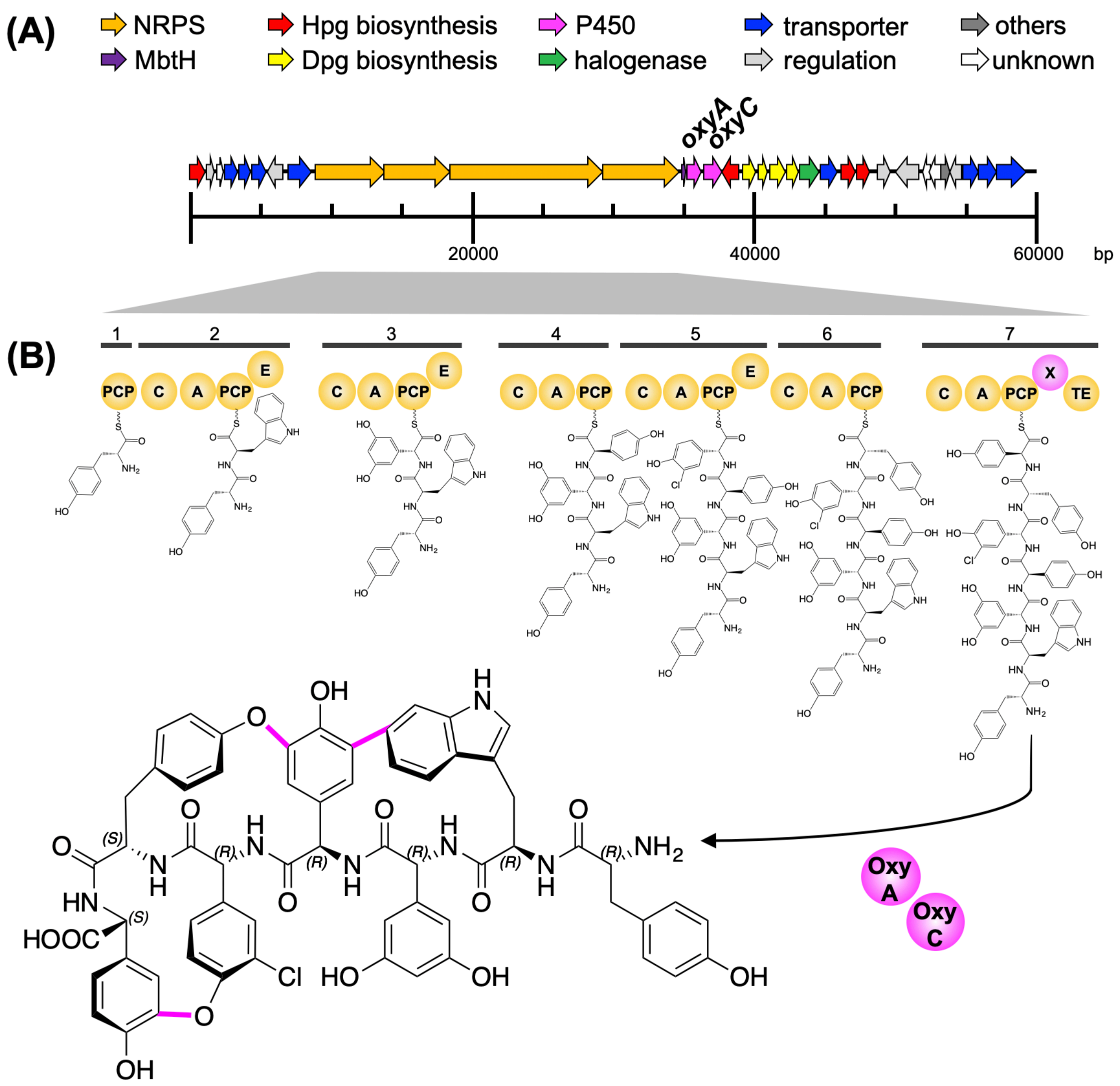

Supplementary Figure 1. Kistamicin biosynthetic gene cluster from *Actinomadura parvosata*

(A) The kistamicin biosynthetic gene cluster from *Actinomadura parvosata* subsp. *kistnae* (*Nono-muraea* sp. ATCC55076) spans around 60 kb and habours four genes (arrows) that encode a non-ribosomal peptide synthetase (orange), an MbtH protein (purple), non-proteinogenic amino acids 4-hydroxyphenylglycine (Hpg, red) and 3,5-dihydroxyphenylglycine (Dpg, yellow), two Cytochrome P450 encoding genes – *oxyA* and *oxyC* (pink), a gene encoding a FAD-type halogenase (green) as well as several transporter genes (blue), two genes code for a two-component system supposed to be involved in regulation of resistance gene expression, three genes encode regulatory proteins belonging to StrR-, LuxR-, and GntR families, respectively (light grey), other functions (dark grey) as well as genes with unknown functions (white). (B) The kistamicin NRPS is comprised of seven modules (module 1-7) that are further subdived into domains (circles) responsible for all catalytic steps involved in formation of the linear heptapeptide precursor. The Oxy enzmes interact with the X domain (pink) found in the last module of the NRPS and catalyse three crosslinking reactions, leading to the final structure of kistamicin. NRPS domain key: A, adenylation domain; C, condensation domain; E, epimerisation domain; PCP, peptidyl carrier protein; TE, thioesterase; X, Oxy recruiting domain.

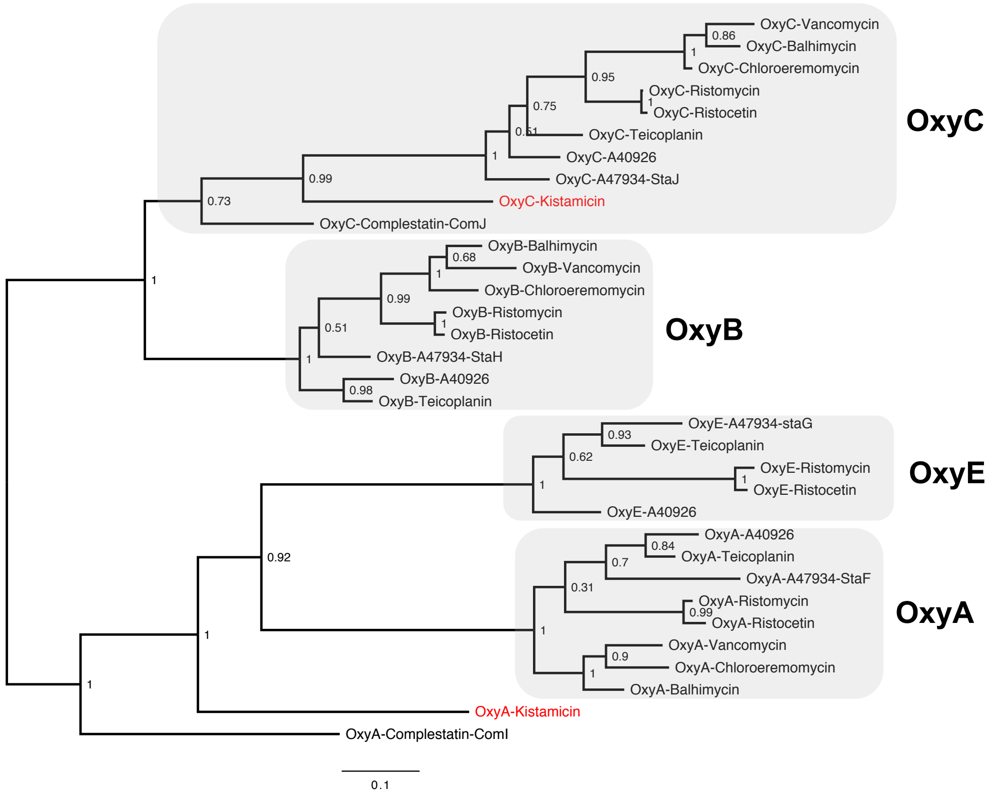

Supplementary Figure 2. Phylogenetic tree of different Oxy enzymes involved in GPA biosynthesis
The protein sequences of the Oxy enyzmes found within the biosynthetic gene clusters of the glycopeptide antibiotics kistamicin (kis), complestatin (com), vancomycin (van), teicoplanin (tei), balhimycin (bal), A47934 (sta), A40926 (dbv), ristomycin/ ristocetin (ris) and chloroeremomycin (cep) were aligned using Muscle[1] before RaxML[2] was used to generate the maximum likelihood phylogeny. Oxy enzymes catalysing similar reactions are evolutionary related to each other and are classified as OxyA, OxyB, OxyC or OxyE enzymes. The kistamicin Oxy enzymes (red) separate (along with the homologous Oxy enyzmes from complestatin biosynthesis) very early within their respective Oxy grouping.

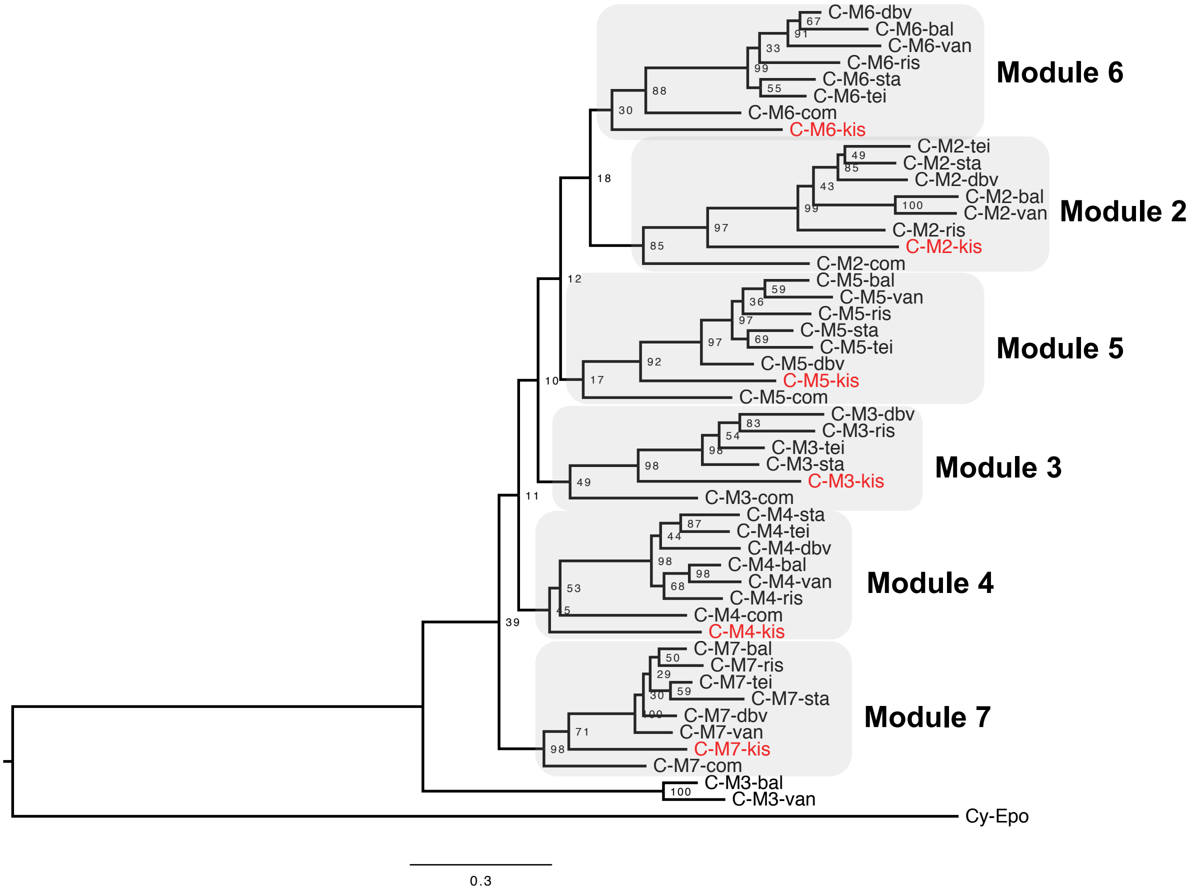

Supplementary Figure 3. Phylogenetic tree of the condensation domains from GPA biosynthesis
The protein sequences of the C-domains contained within the NRPS machinery of the glycopeptide antibiotics kistamicin (kis), complestatin (com), vancomycin (van), teicoplanin (tei), balhimycin (bal), A47934 (sta), A40926 (dbv), ristomycin (ris) and chloroeremomycin (cep) were aligned using Muscle[1] before RaxML[2] was used to generate the maximum likelihood phylogeny. Results show that the C-domains cluster within the modules of the NRPS in which they are found (with the exception of modules 3 from the vancomycin and balhimycin clusters), with the complestatin and kistamicin (red) C-domains branching the earliest within each clade.

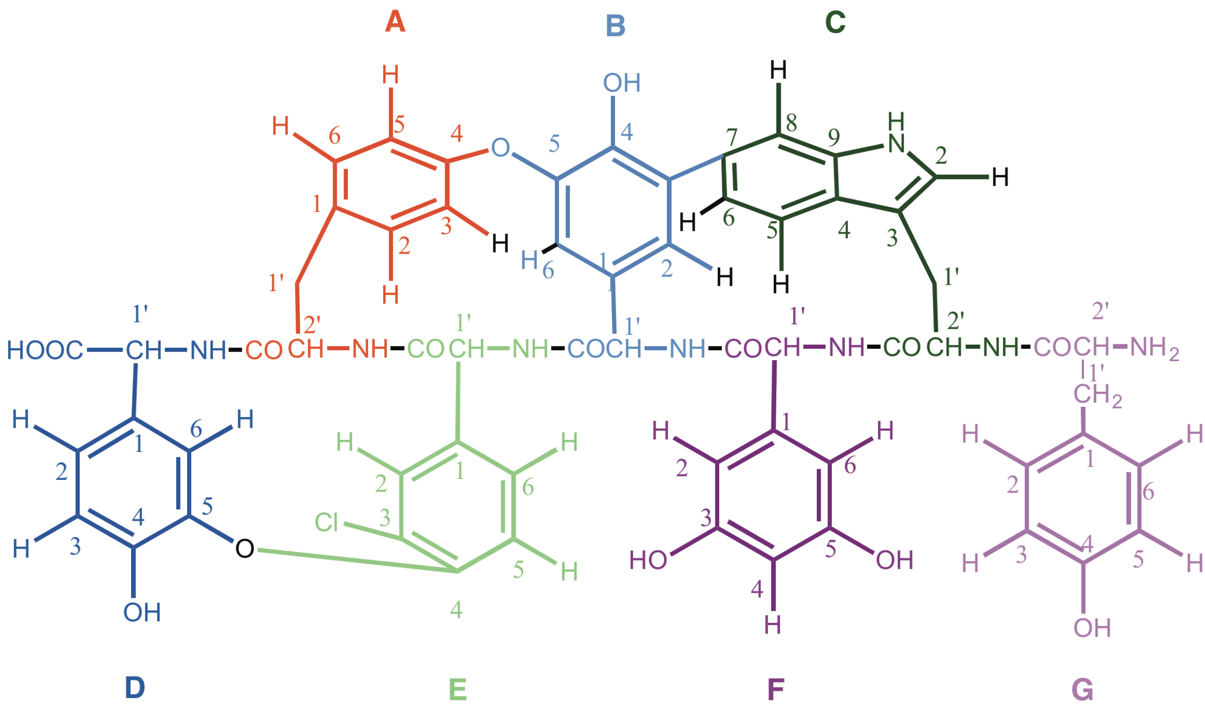

Supplementary Figure 4. Numbering of atoms within kistamicin. Shows the structure of kistamicin with numbering that is used to identify the signals within the NMR data

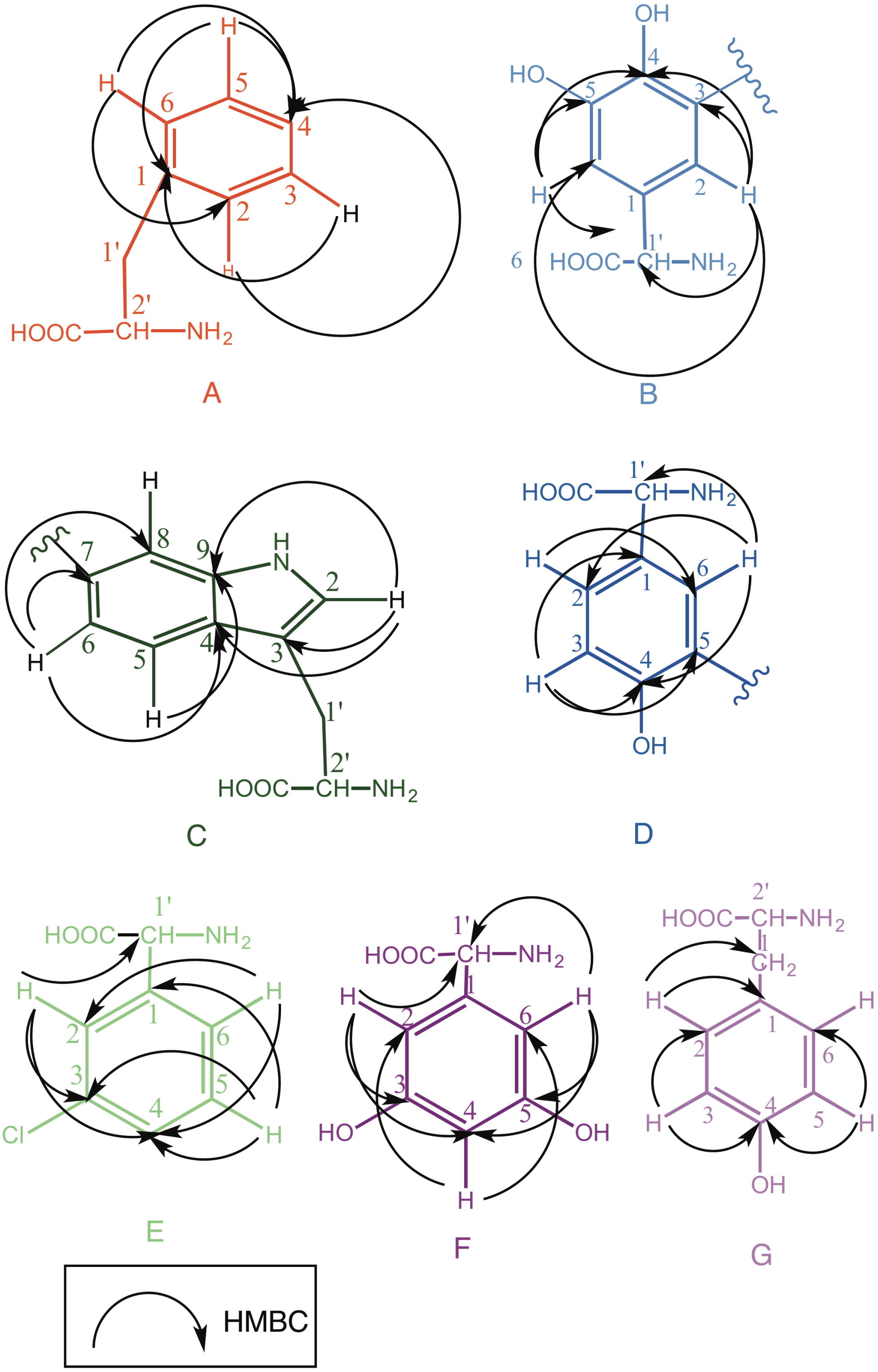

Supplementary Figure 5. HMBC correlations observed in the structure of kistamicin. Each ring (A-G) within the kistamicin structure showing the HMBC correlations identified.

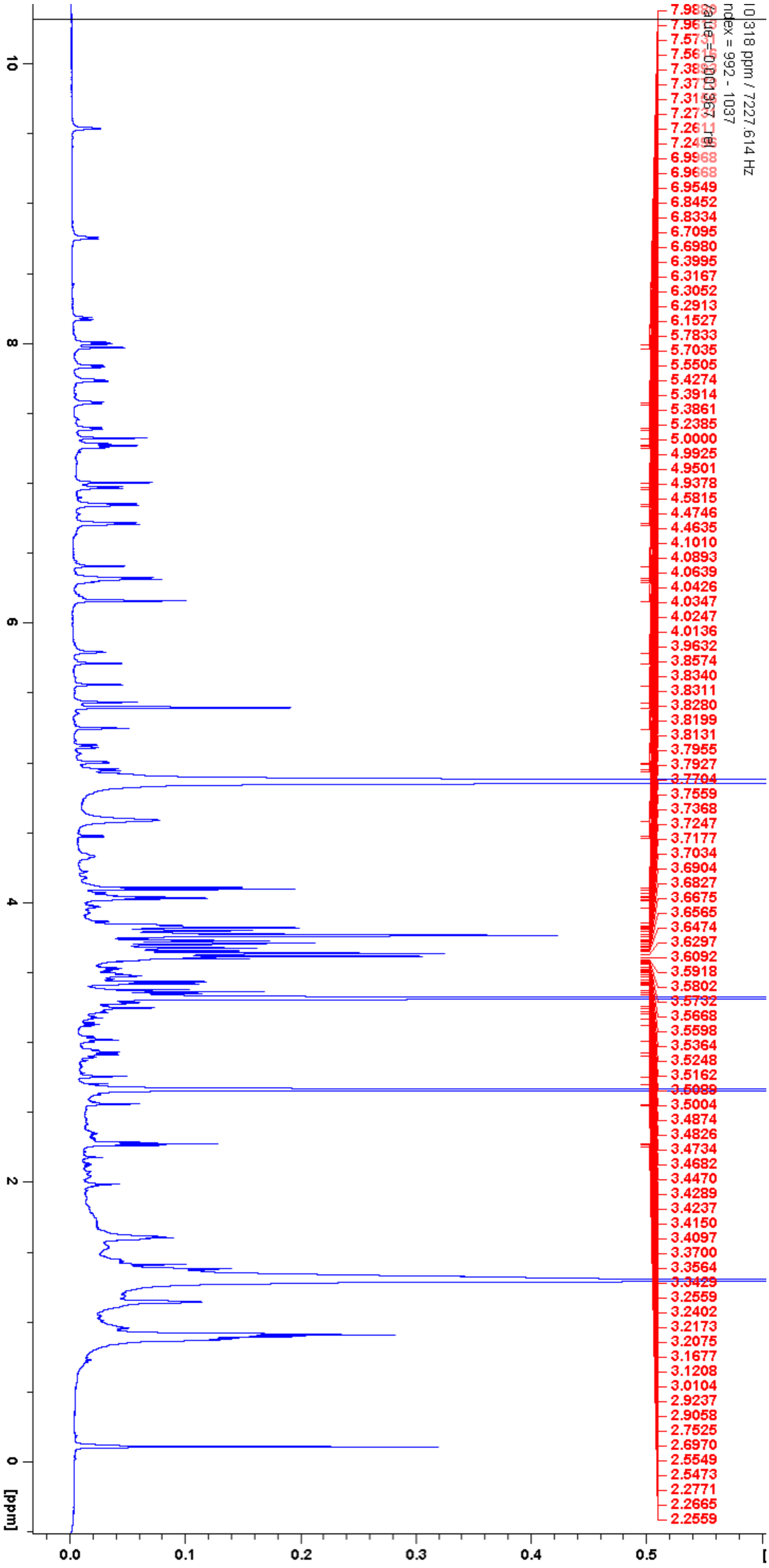

Supplementary Figure 6. ^{1}H NMR spectra for kistamicin

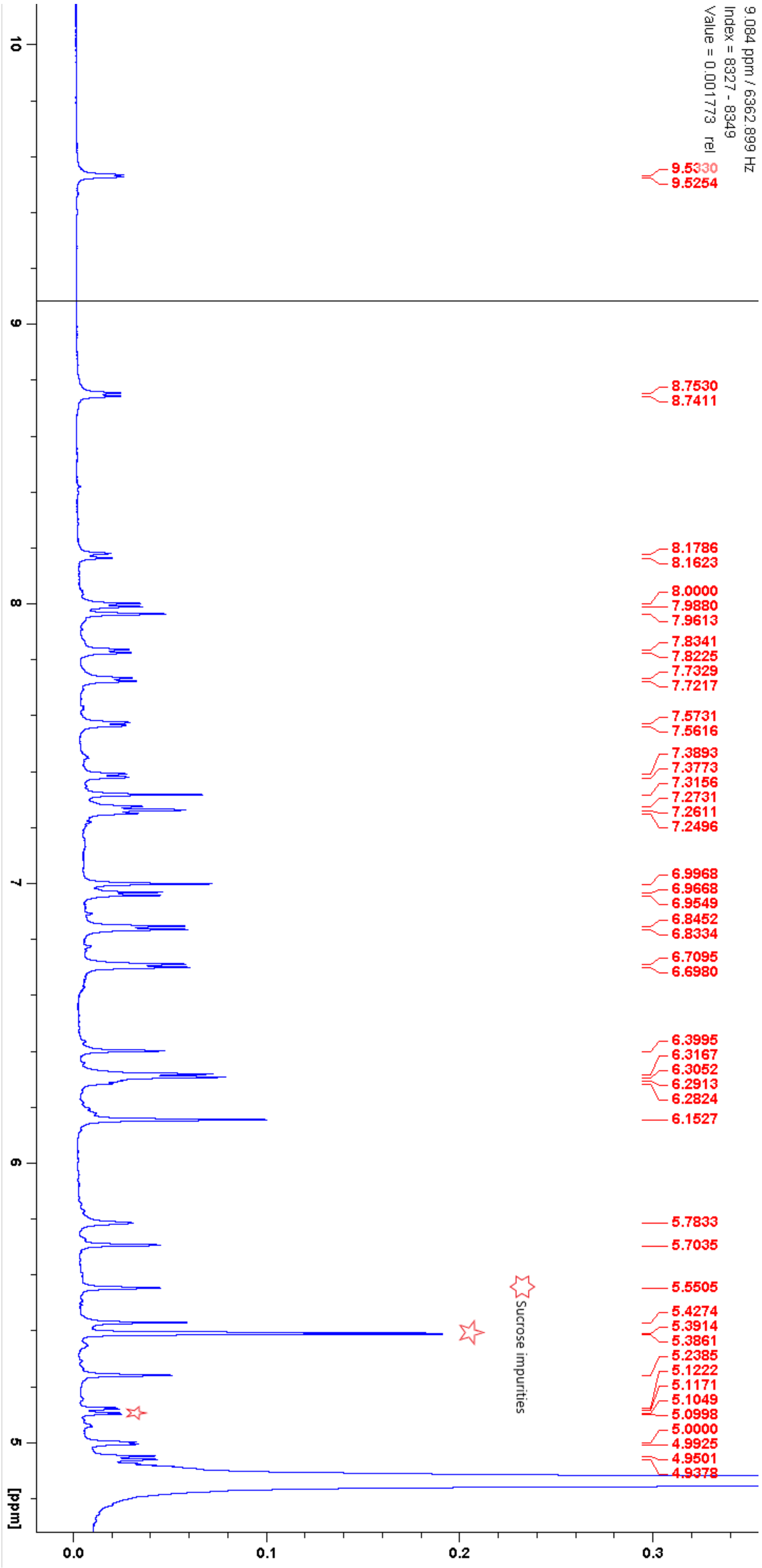

Supplementary Figure 7. Expanded ^{1}H NMR trace for kistamicin (4.7 – 10.2 ppm)

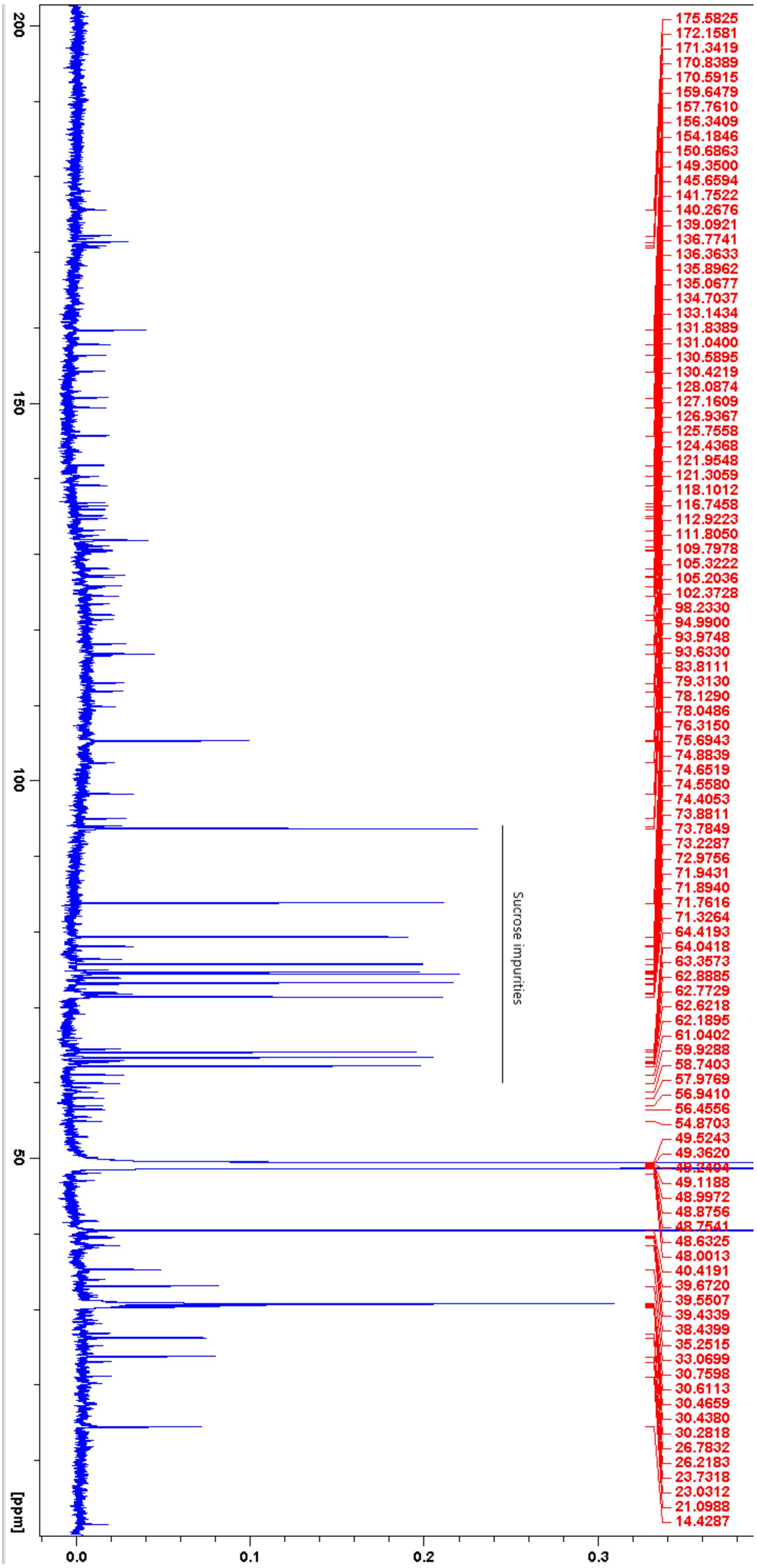

Supplementary Figure 8. ^{13}C NMR trace for kistamicin

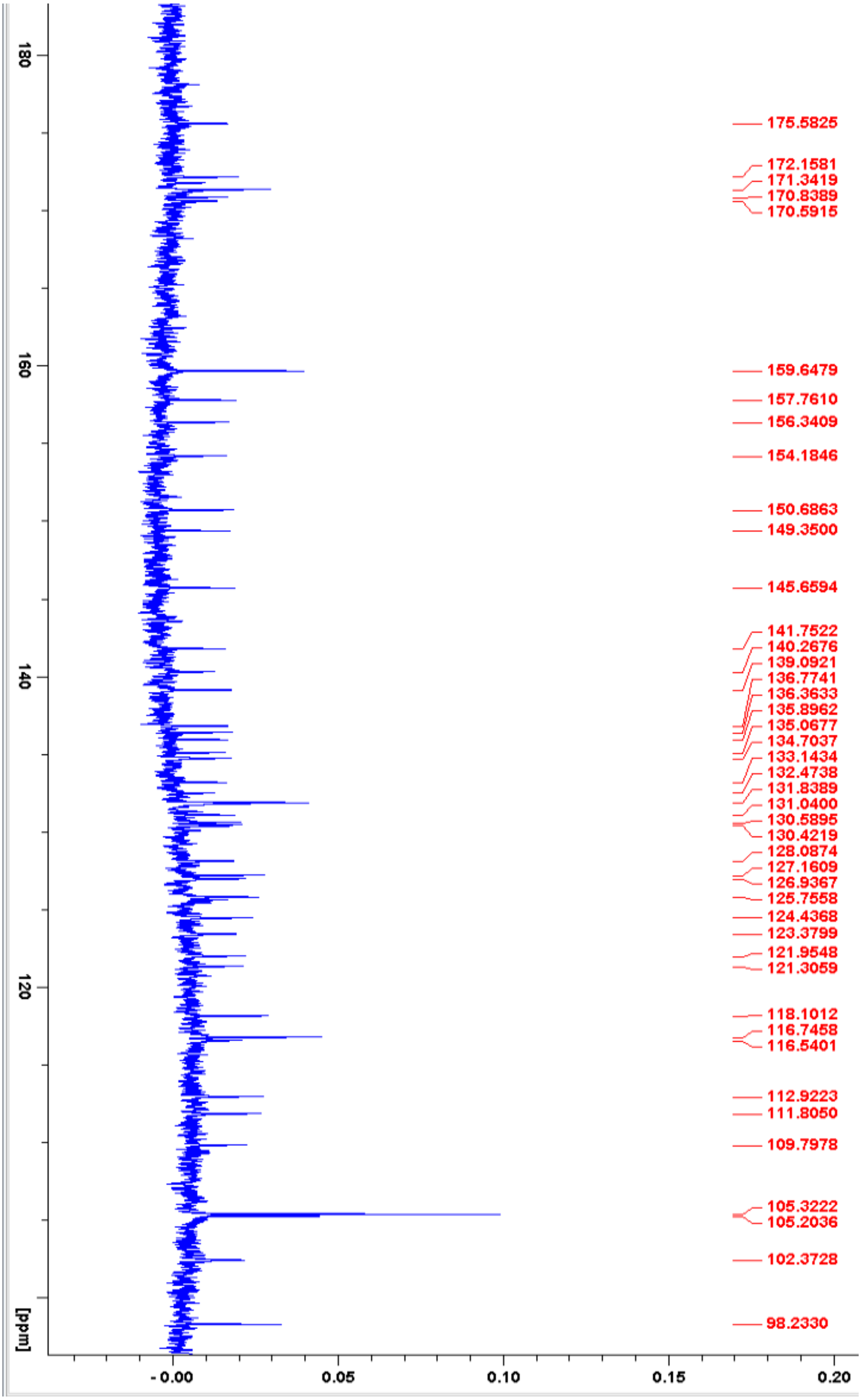

Supplementary Figure 9. Expanded ^{13}C NMR trace for kistamicin (90 – 190 ppm)

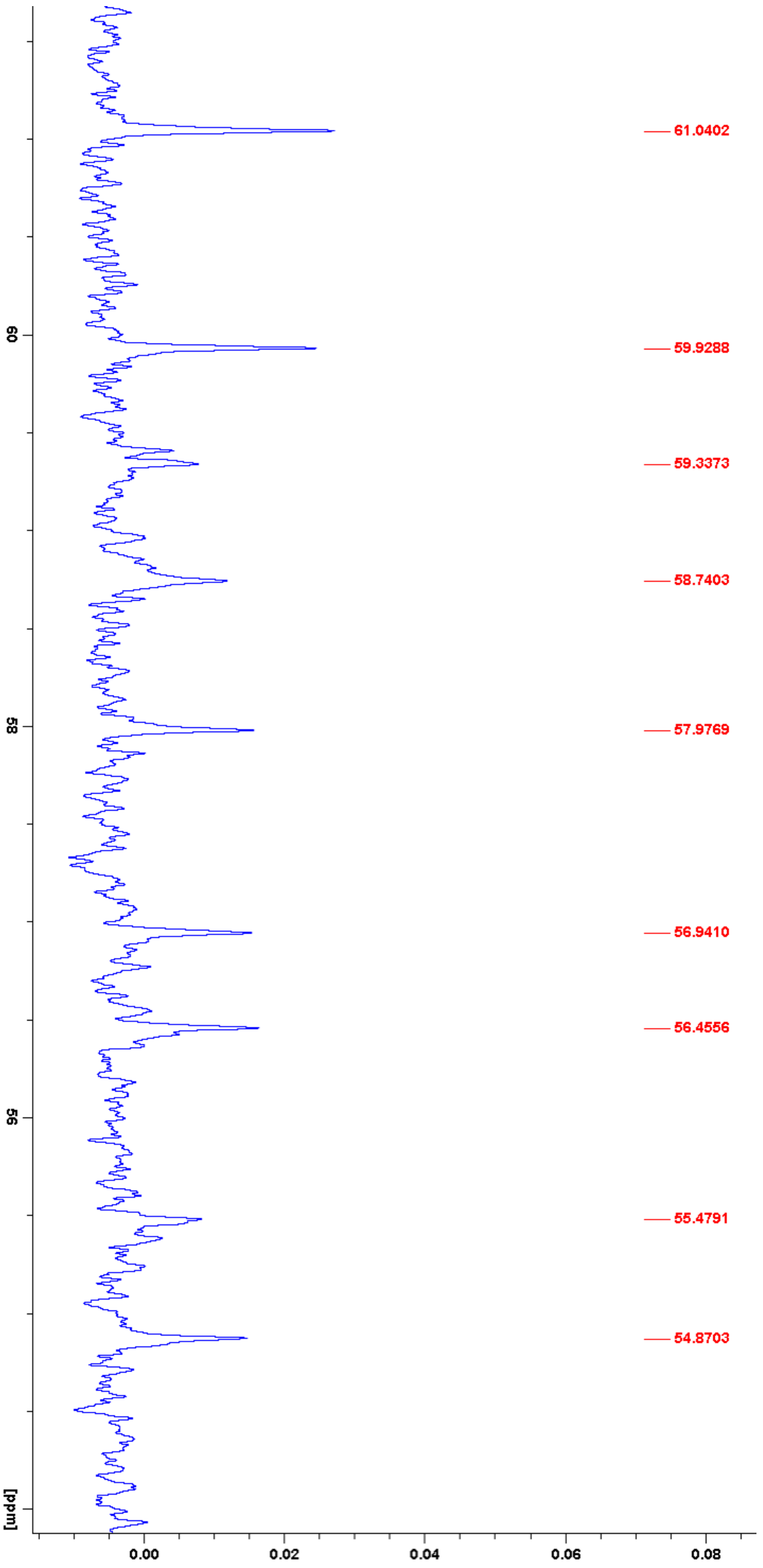

Supplementary Figure 10. Expanded ^{13}C NMR spectra for kistamicin (54 – 62 ppm)

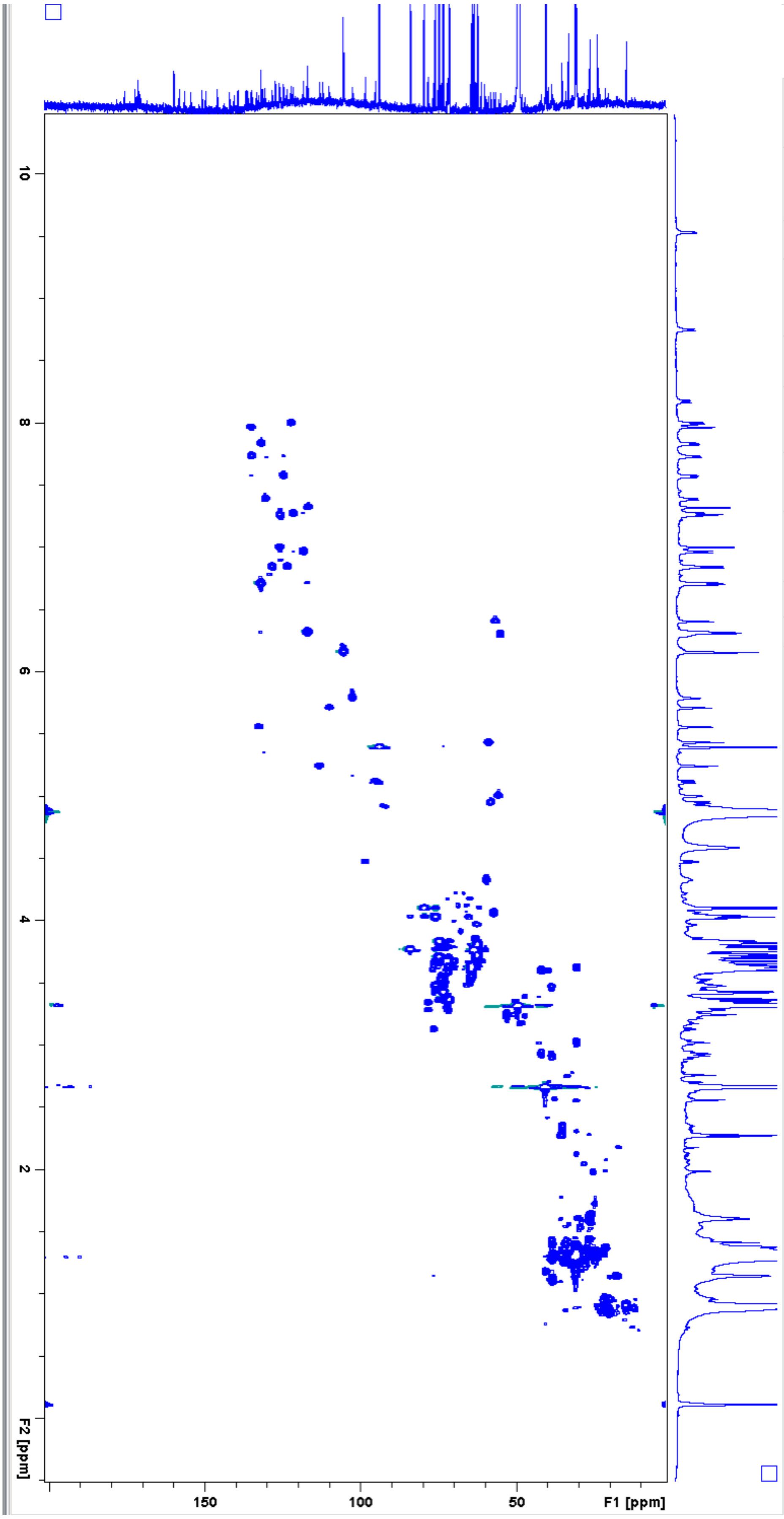

Supplementary Figure 11. HSQC spectra for kistamicin

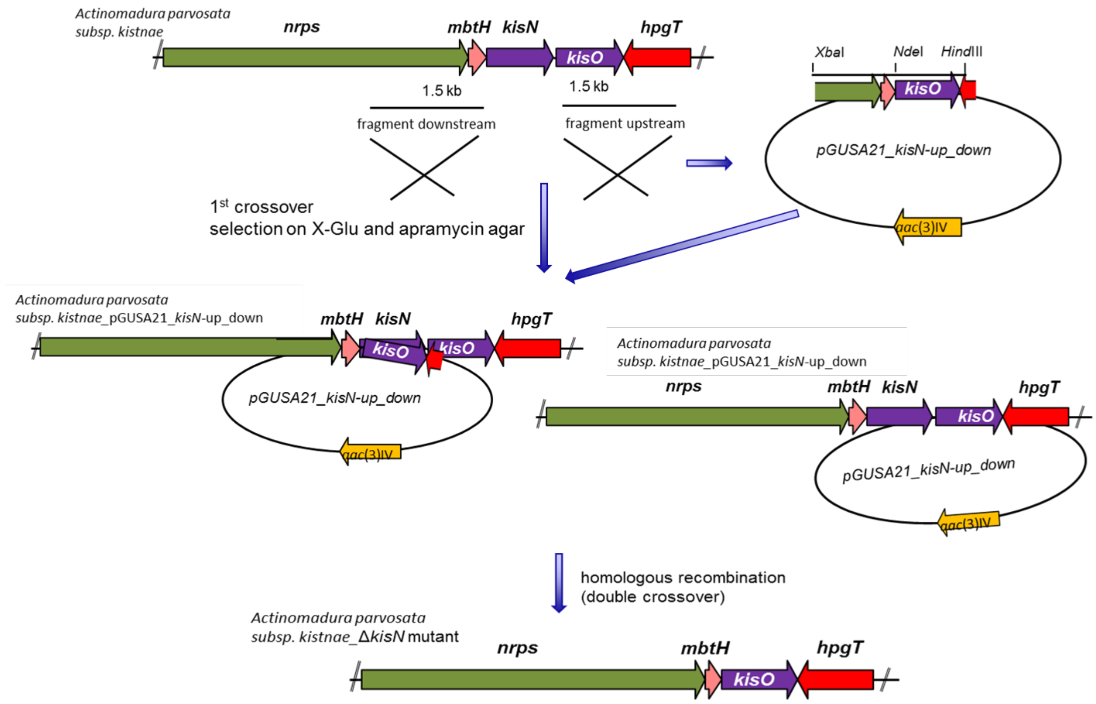

Supplementary Figure 12. Schematic representation of the *kisN* (*oxyA*) deletion strategy

The 1.5 kb upstream and downstream fragments of *kisN* (*oxyA*) gene were cloned into pGUSA21 vector. The 1st crossover occurrence relies on homologous recombination of the plasmid pGUSA21_*kisN*-up_down. The selection of mutants where the plasmid is integrated into the chromosome occurs on apramycin and X-gluc (5-bromo-4-chloro-1*H*-indol-3-yl β-D-glucopyranosid-uronic acid; blue colonies) containing plates. The 2nd crossover causes the disintegration of the plasmid and eventually the deletion of *kisN*. It can be provoked by exposing the cells to stress (e.g. formation of protoplasts, cultivation at 39 °C). The occurrence of the 2nd crossover is proven by X-gluc selection (red colonies) (Supplementary Figure 13A) and PCR analyses (Supplementary Figure 14). The same procedure was applied for deletion of *kisO* gene (Supplementary Figure 13B and Supplementary Figure 16).

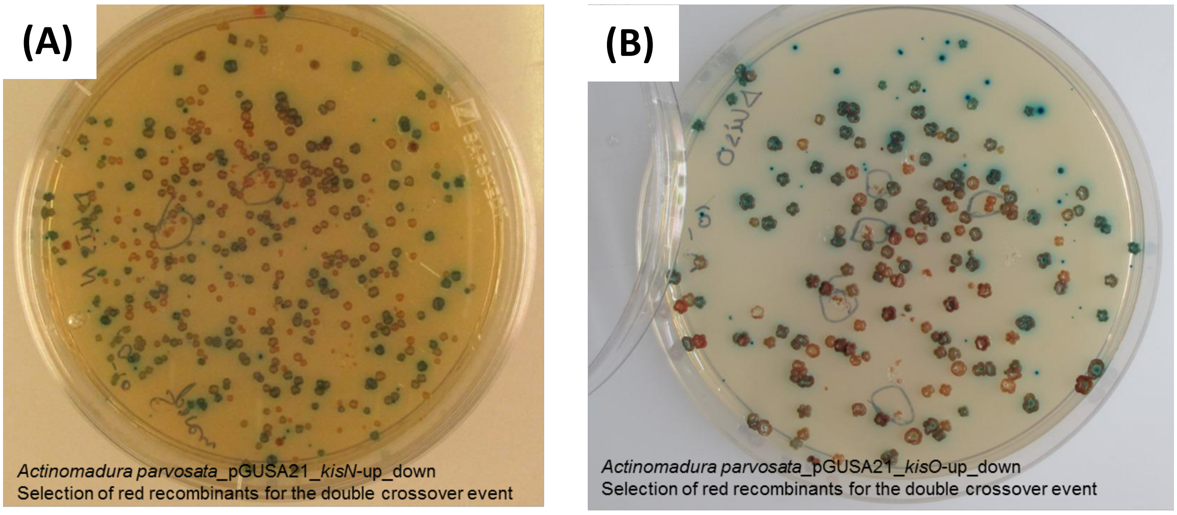

Supplementary Figure 13. Screening of *Actinomadura parvosata* mutants

Growth of *Actinomadura parvosata* subsp. *kistnae*_pGUSA21_*kisN*-up_down and *A. parvosata* subsp. *kistnae*_pGUSA21_*kisO*-up_down protoplasts and X-gluc (5-bromo-4-chloro-1H-indol-3-yl-Beta-D-glucuronic acid) screening for deletion mutants *A. parvosata* Δ*kisN* (A) and *A. parvosata* Δ*kisO* (B) on R5 agar plates (Ø10 cm) overlaid with 20 mM X-gluc.

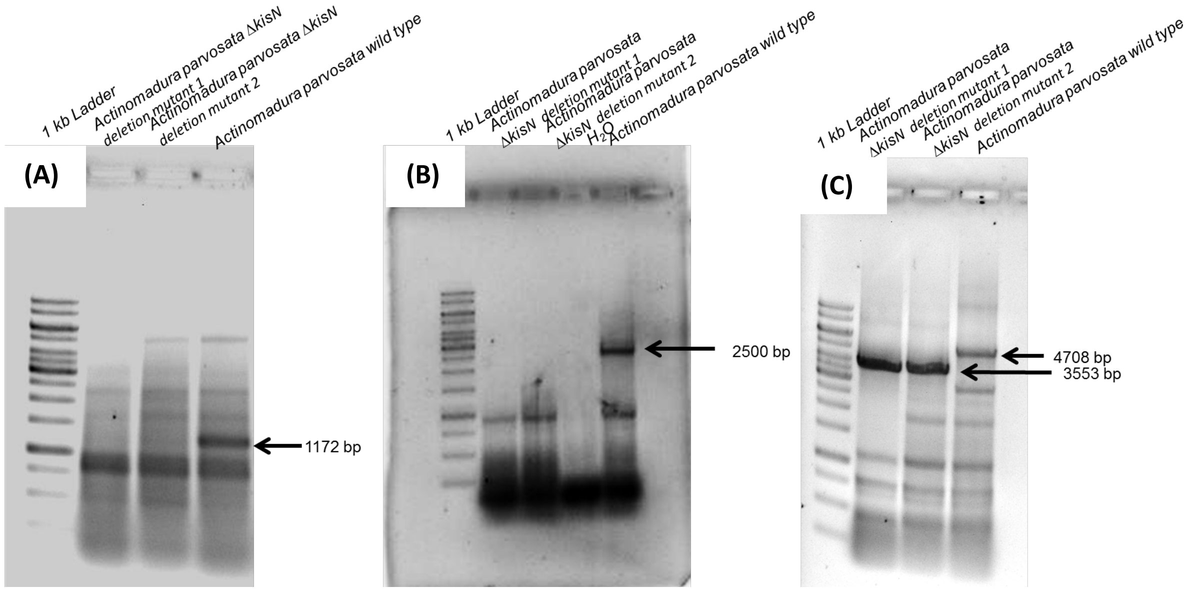

Supplementary Figure 14. PCR Analysis for verification of the in-frame deletion of *kisN*

The in-frame deletion was confirmed for two *Actinomadura parvosata ΔkisN* mutants. (A) lane 1: Marker, 1 kb ladder; lane 2 and 3: *A. parvosata*_ΔkisN DNA: no amplicon for *kisN* gene; lane 4: wild type DNA: amplification of a 1172 bp fragment. The primer pair kisN-NdeI-Fw/kisN-HindIII-Rv was used. (B) lane 1: Marker, 1 kb ladder; lane 2 and 3: *A. parvosata*_ΔkisN DNA: no amplicon; lane 4: H_2O; lane 5: wild type DNA: amplification of a 2500 bp fragment. In all cases, the primer pair Delta kisN Fw/ Delta kisN-Rv was used. (C) lane 1: Marker, 1 kb ladder; lane 2 and 3: *A. parvosata_ΔkisN* DNA amplification of a 3553 bp fragment; lane 4: wild type DNA: amplification of a 4708 bp fragment. The primer pair Delta kisN Fw/kisO-XbaI-Rv was used. Primers are listed in Supplementary Table 3**.**

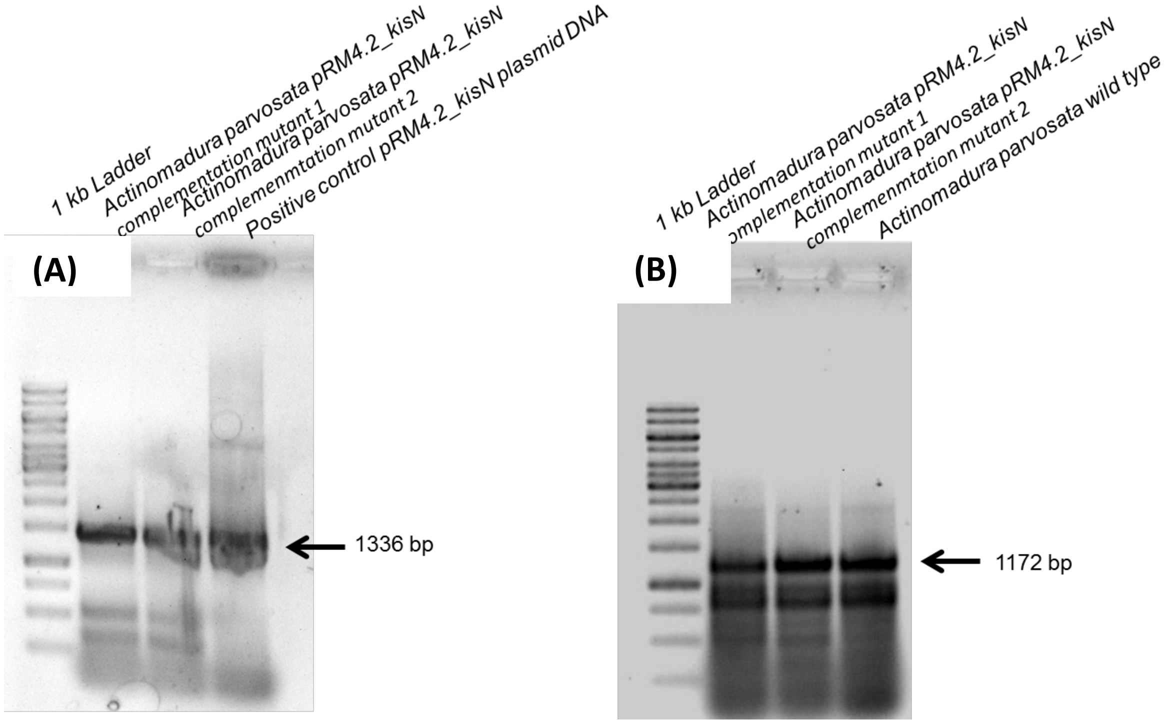

Supplementary Figure 15. PCR-Verification of *kisN*-complementation

The integration of pRM4.2_*kisN*-plasmid was confirmed for two *A. parvosata* pRM4.2_kisN complemented mutants. (A) lane 1 Marker, 1 kb ladder; lane 2 and 3: DNA of *A. parvosata* pRM4.2_*kisN* complemented mutants 1 and 2: amplification of a 1336 bp fragment, lane 4: pRM4.2_*kisN* plasmid DNA: amplification of a 1336 bp fragment: In all cases, the primer pair pRM4SeqFw/pRM4SeqRv was used. (B) lane 1: Marker, 1 kb ladder; lane 2 and 3: DNA of *A. parvosata* pRM4.2_*kisN* complemented mutants 1 and 2: amplification of a 1172 bp fragment; lane 4: *A. parvosata* wild type DNA: amplification of a 1172 bp fragment. The primer pair kisN-NdeI-Fw/kisN-HindIII-Rv was used. Primers are listed in Supplementary Table 3**.**

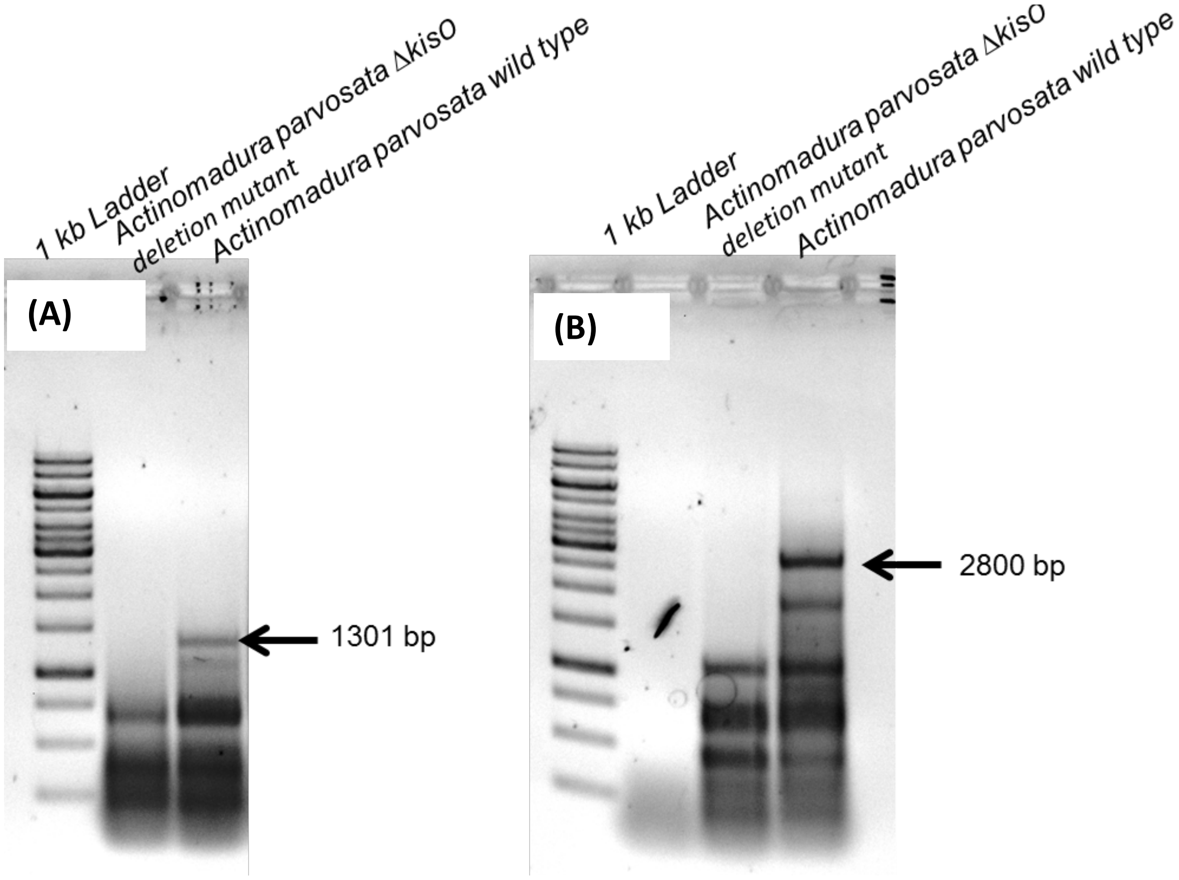

Supplementary Figure 16. PCR Analysis for verification of the in-frame deletion of *kisO*

The in-frame deletion was confirmed for one *Actinomadura parvosata_ΔkisO* mutant. (A) lane 1: Marker, 1 kb ladder; lane 2: *A._ΔkisO* DNA: no amplicon for *kisO* gene; lane 3: wild type DNA: amplification of a 1301 bp fragment. In all cases, the primer pair kisO-HindIII-Fw / kisO-XbaI-Rv was used. (B) lane 1: Marker, 1 kb ladder; lane 2: *A. parvosata_ΔkisO* DNA: no amplicon; lane 3: wild type DNA: amplification of a 2800 bp fragment. The primer pair Delta kisO Fw/ Delta kisO-Rv was used. Primers are listed in Supplementary Table 3**.**

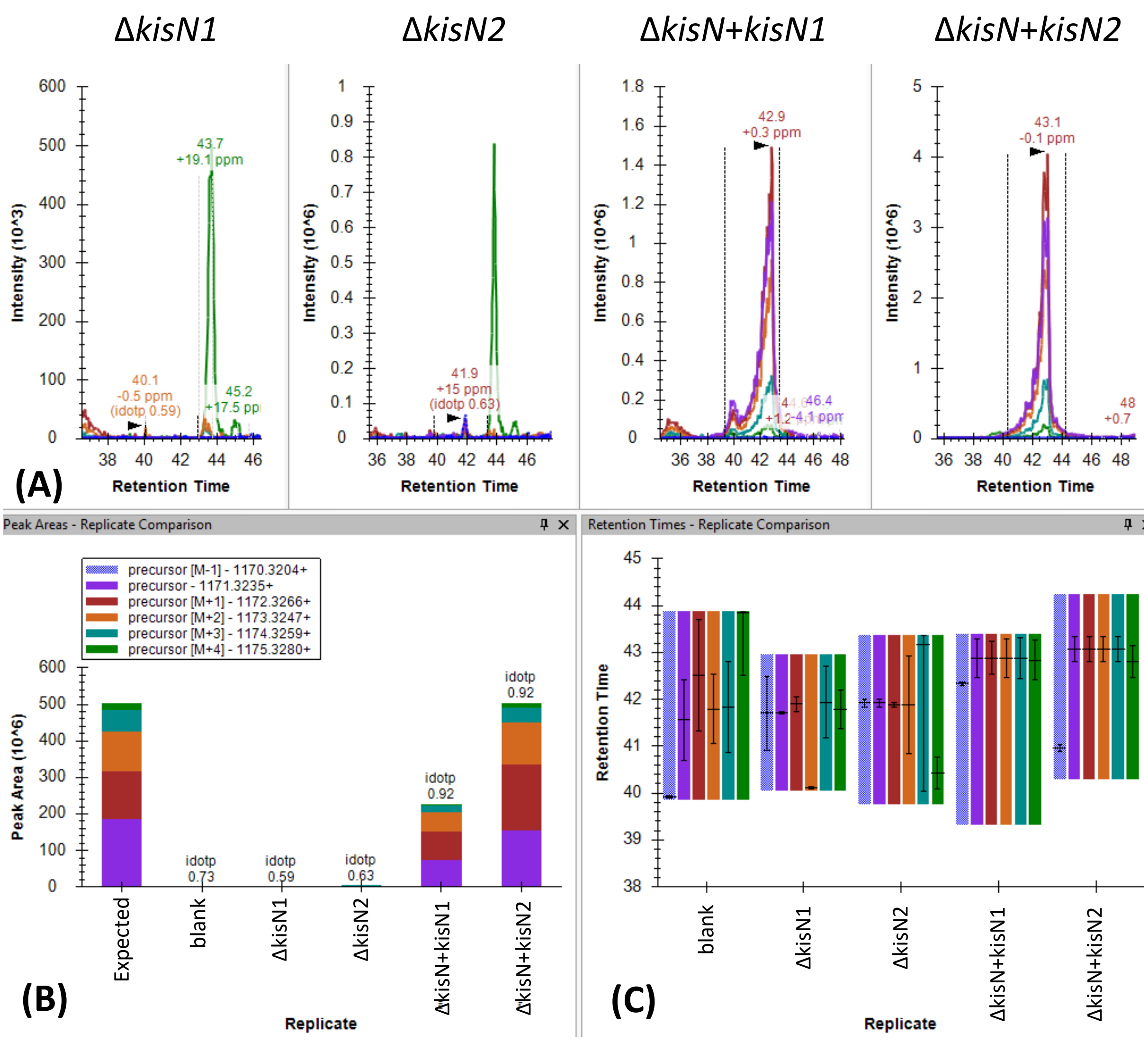

Supplementary Figure 17. Production of kistamicin in the *Actinomadura parvosata* mutants

Two different mutants (1 + 2) of *Actinomadura parvosata* Δ*kisN* and the complemented mutant Δ*kisN* + *kisN* (1+2) were extracted and analysed by HRMS. The specific kistamicin A (1171.3225 $[M+H]^+$) metabolite and its isotopic peaks from different producer strains were analysed. Isotopic patterns are important to identify the correct kistamicin species. The purple colour indicates the $[M+H]^+$ isotope, with other colours represent different isotopes (blue -1, red +1, orange +2, cyan +3, green +4), respectively. (A) Extracted ion chromatogram of the respective compound in the different mutants. (B) Integrated peak areas of the respective compound peak in comparison to the expected (calculated) isotope contribution between 36 – 48 min; this is normalised to the intensity of the highest intensity peak in the different strains for comparison. (C) Retention times of the respective kistamicin intermediates and the peak width of these species; black lines indicate indicate retention times where these ions were at their maximum intensity within the total peak width. The deletion of *kisN* lead to the loss of kistamicin in the *Actinomadura parvosata* Δ*kisN* mutants, which is then restored through complementation of these mutants with a plasmid containing the *kisN* gene.

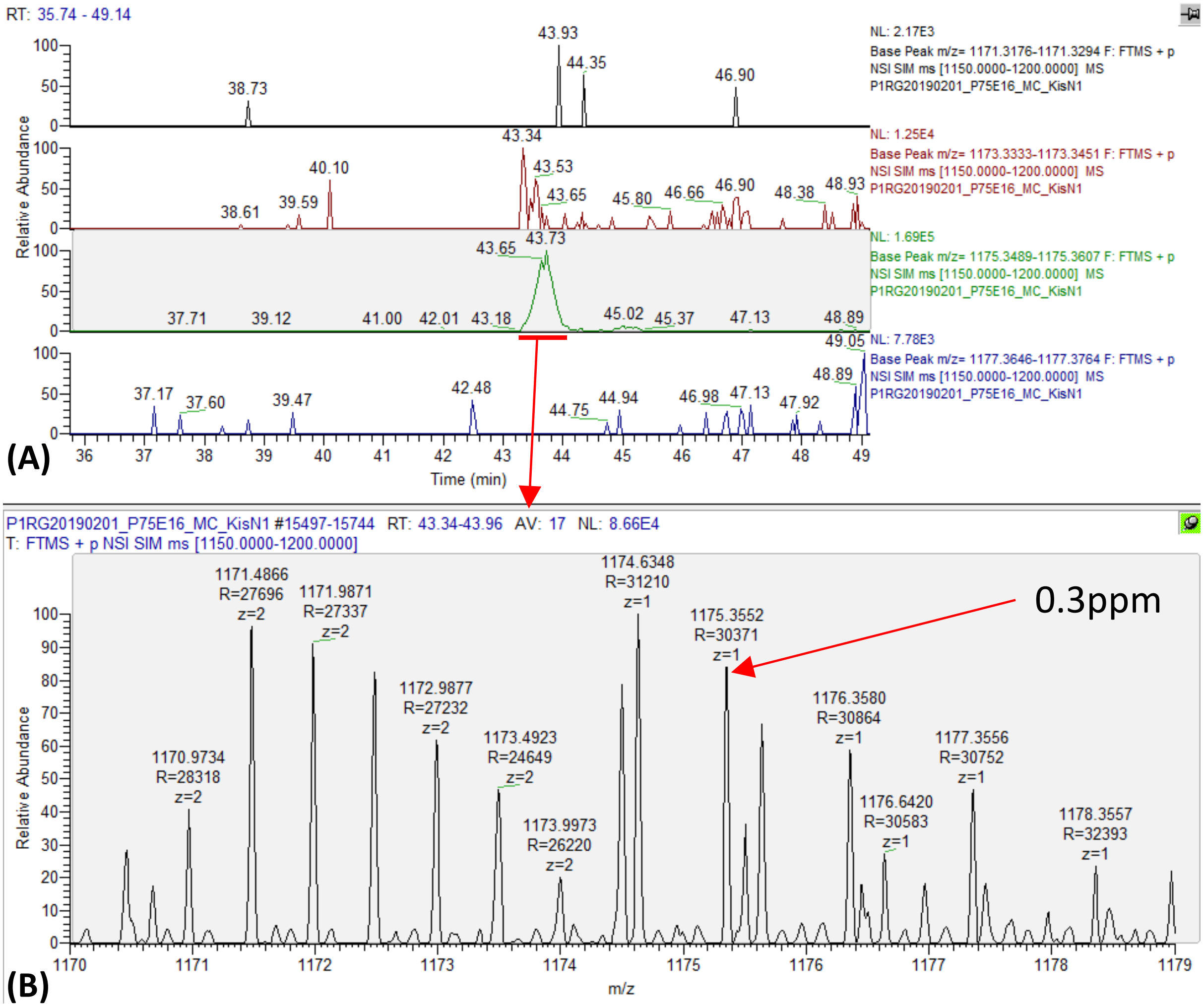

Supplementary Figure 18. Production of kistamicin intermediates in *A. parvosata ΔkisN*

(A) Extracted ion chromatograms for kistamicin A, 1171.3235 $[M+H]^+$ (black); bicyclic heptapeptide, 1173.3392 $[M+H]^+$ (red); monocyclic heptapeptide, 1175.3548 $[M+H]^+$ (green); linear heptapeptide, 1177.3705 $[M+H]^+$ (blue) with 5 ppm tolerance; (B) HRMS spectrum of of detected monocyclic heptapeptide 1175.3548 $[M+H]^+$ with 0.3 ppm mass error.

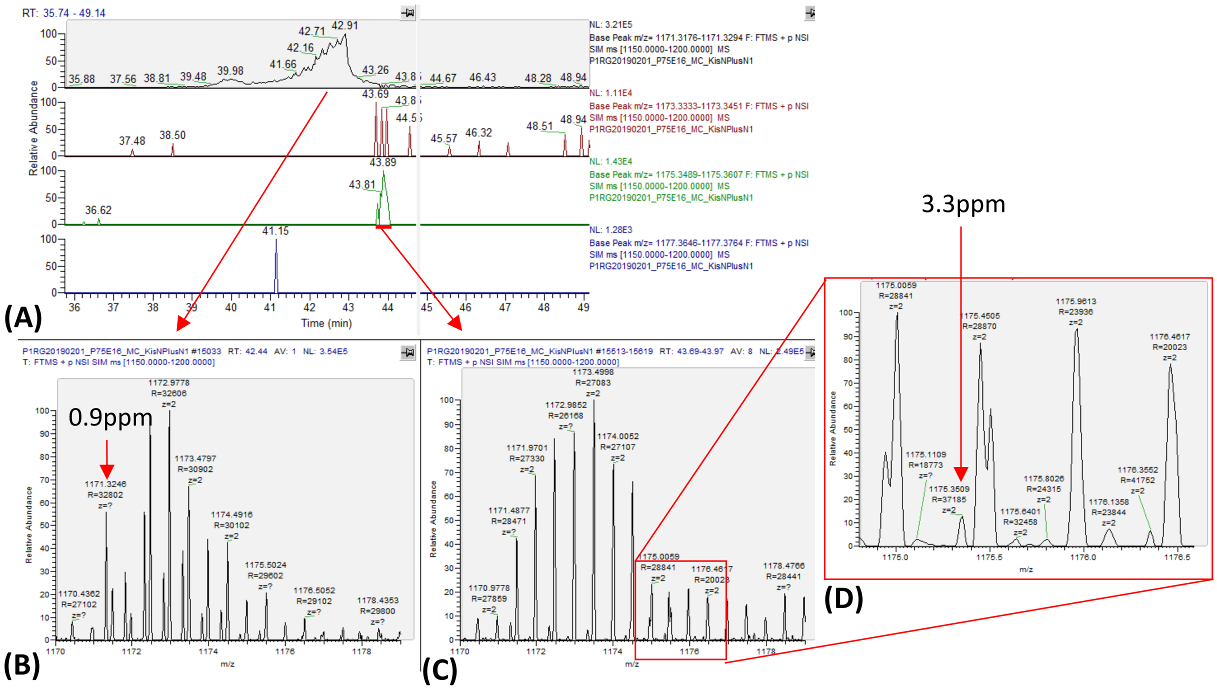

Supplementary Figure 19. Production of kistamicin intermediates in *A. parvosata ΔkisN* + *kisN*

(A) Extracted ion chromatograms for kistamicin A, 1171.3235 $[M+H]^+$ (black); bicyclic heptapeptide, 1173.3392 $[M+H]^+$ (red); monocyclic heptapeptide, 1175.3548 $[M+H]^+$ (green); linear heptapeptide, 1177.3705 $[M+H]^+$ (blue) with 5 ppm tolerance. (B) HRMS spectrum of detected fully cyclised kistamicin with a mass of 1171.3235 $[M+H]^+$ with 0.9 ppm mass error. (C) Close up and (D) highly zoomed views of the HRMS spectrum of monocyclic kistamicin: this has a mass of 1175.3548 $[M+H]^+$ with mass error of 3.3 ppm, indicating that the complemented mutant fully restored kistamicin cyclisation.

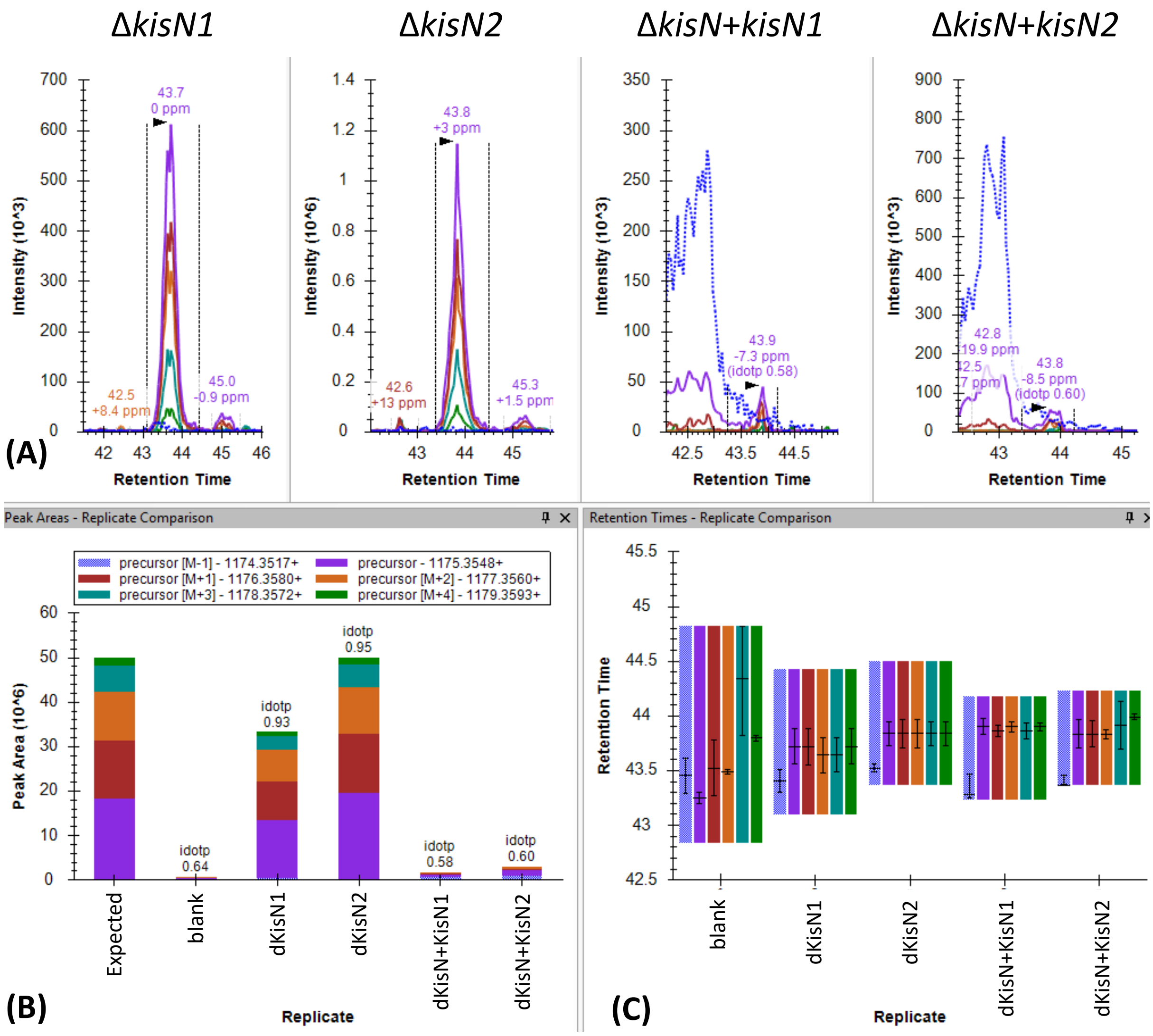

Supplementary Figure 20. Production of kistamicin monocyclic (C-*O*-D) heptapeptide

Two different mutants (1 + 2) of *Actinomadura parvosata* Δ*kisN* and the complemented mutant Δ*kisN* + *kisN* (1+2) were extracted and analysed by HRMS. The kistamicin monocyclic heptapeptide 1175.3548 $[M+H]^+$ and its isotopic peaks from different producer strains were analysed. Isotopic patterns are important to identify the correct kistamicin species. The purple colour indicates the $[M+H]^+$ isotope, with other colours represent different isotopes (blue -1, red +1, orange +2, cyan +3, green +4), respectively. (A) Extracted ion chromatogram of the respective compound in the different mutants. (B) Integrated peak areas of the respective compound peak in comparison to the expected (calculated) isotope contribution between 42 – 46 min; this is normalised to the intensity of the highest intensity peak in the different strains for comparison. (C) Retention times of the respective kistamicin intermediates and the peak width of these species; black lines indicate indicate retention times where these ions were at their maximum intensity within the total peak width. The monocyclic heptapeptide is present in significant amounts in the *Actinomadura parvosata* Δ*kisN* mutants. Complementation of these mutants with a plasmid containing the *kisN* gene then leads to a large reduction of this intermediate that is explained by the functional biosynthetic route in this case leading to production of kistamicin A.

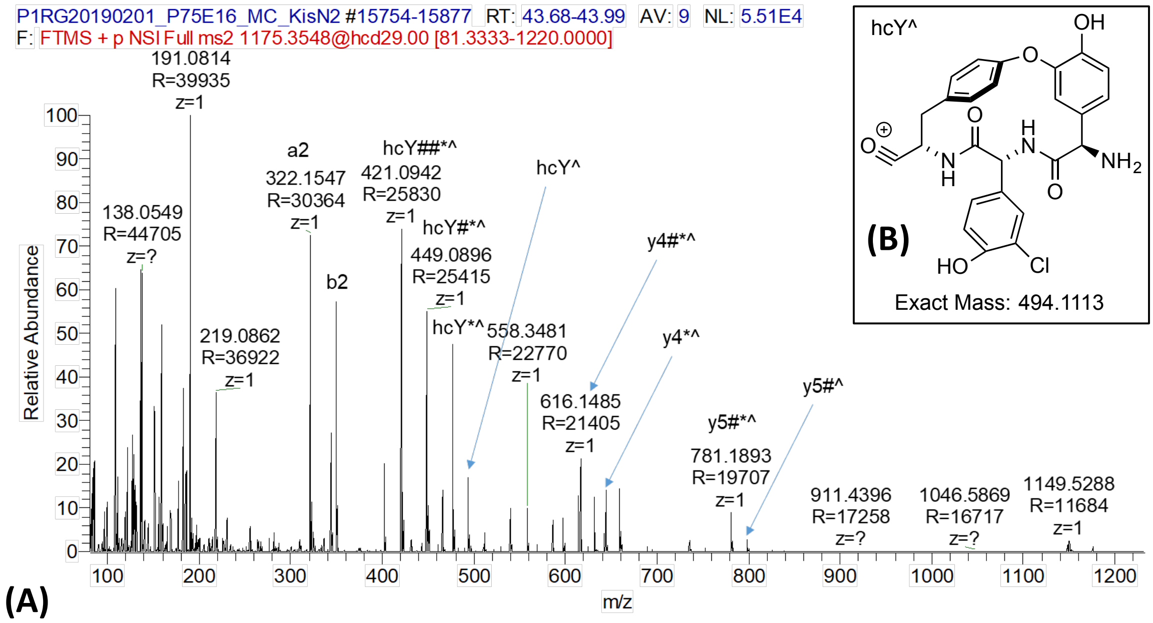

Supplementary Figure 21. MSMS spectrum of monocyclic heptapeptide of *A. parvosata ΔkisN*

The *Actinomadura parvosata ΔkisN* mutant produces a monocyclic heptapeptide with a Hpg4-Tyr6 crosslink, as observed by the presence of a crosslink on the internal ion, Hpg4-Tyr6 (hcY). (A) MSMS spectrum of monocyclic heptapeptide with a mass of 1175.3548 [M+H]+. (B) Structure of fragment hcY^ (mass 494.1113 Da) identified in the spectrum that indicates the crosslink is found between Hpg4-Tyr6 in the monocyclic heptapeptide of *Actinomadura parvosata ΔkisN* mutant. Symbols: Y tyrosine; W tryptophan; h Hpg; d Dpg; c chloroHpg; # CO loss; * NH_3 loss; ^ crosslink (H_2 loss); The b ions extend from the N-terminus, and y ions extend from C-terminus. Fragmentation ions follow standard nomenclature for such experiments, see [3,4].

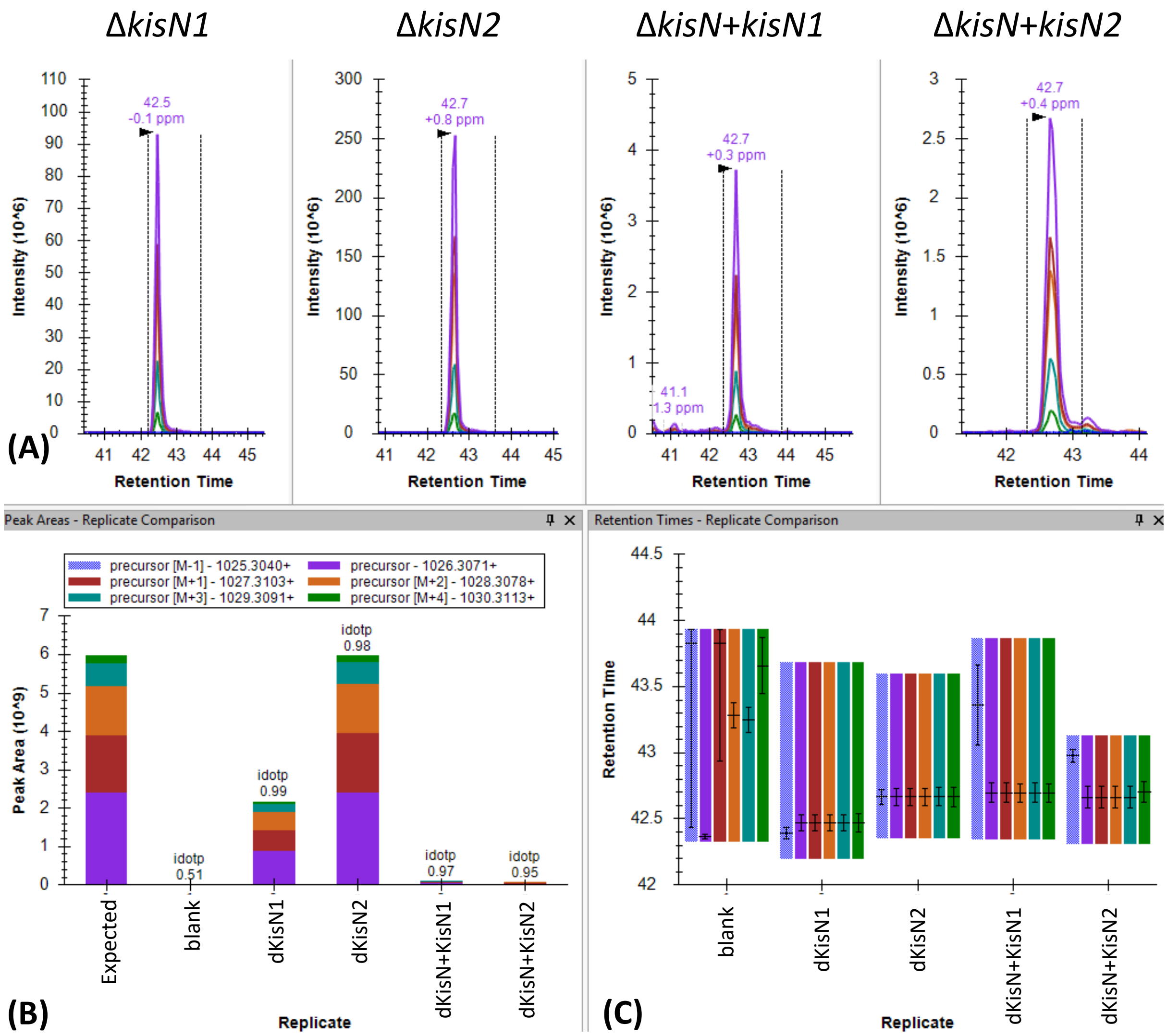

Supplementary Figure 22. Production of kistamicin monocyclic 1-6 hexapeptide (C-*O*-D)
Two different mutants (1 + 2) of *Actinomadura parvosata* Δ*kisN* and the complemented mutant Δ*kisN* + *kisN* (1+2) were extracted and analysed by HRMS. The kistamicin monocyclic hexapeptide (1026.3071 $[M+H]^+$) and its isotopic peaks from different producer strains were analysed. Isotopic patterns are important to identify the correct kistamicin species. The purple colour indicates the $[M+H]^+$ isotope, with other colours represent different isotopes (blue -1, red +1, orange +2, cyan +3, green +4), respectively. (A) Extracted ion chromatogram of the respective compound in the different mutants. (B) Integrated peak areas of the respective compound peak in comparison to the expected (calculated) isotope contribution between 40 – 45 min; this is normalised to the intensity of the highest intensity peak in the different strains for comparison. (C) Retention times of the respective kistamicin intermediates and the peak width of these species; black lines indicate indicate retention times where these ions were at their maximum intensity within the total peak width. The monocyclic (C-*O*-D) hexapeptide is present in significant amounts in the *Actinomadura parvosata* Δ*kisN* mutants. Complementation of these mutants with a plasmid containing the *kisN* gene then leads to a large reduction of this intermediate that is explained by the functional biosynthetic route in this case leading to production of kistamicin A.

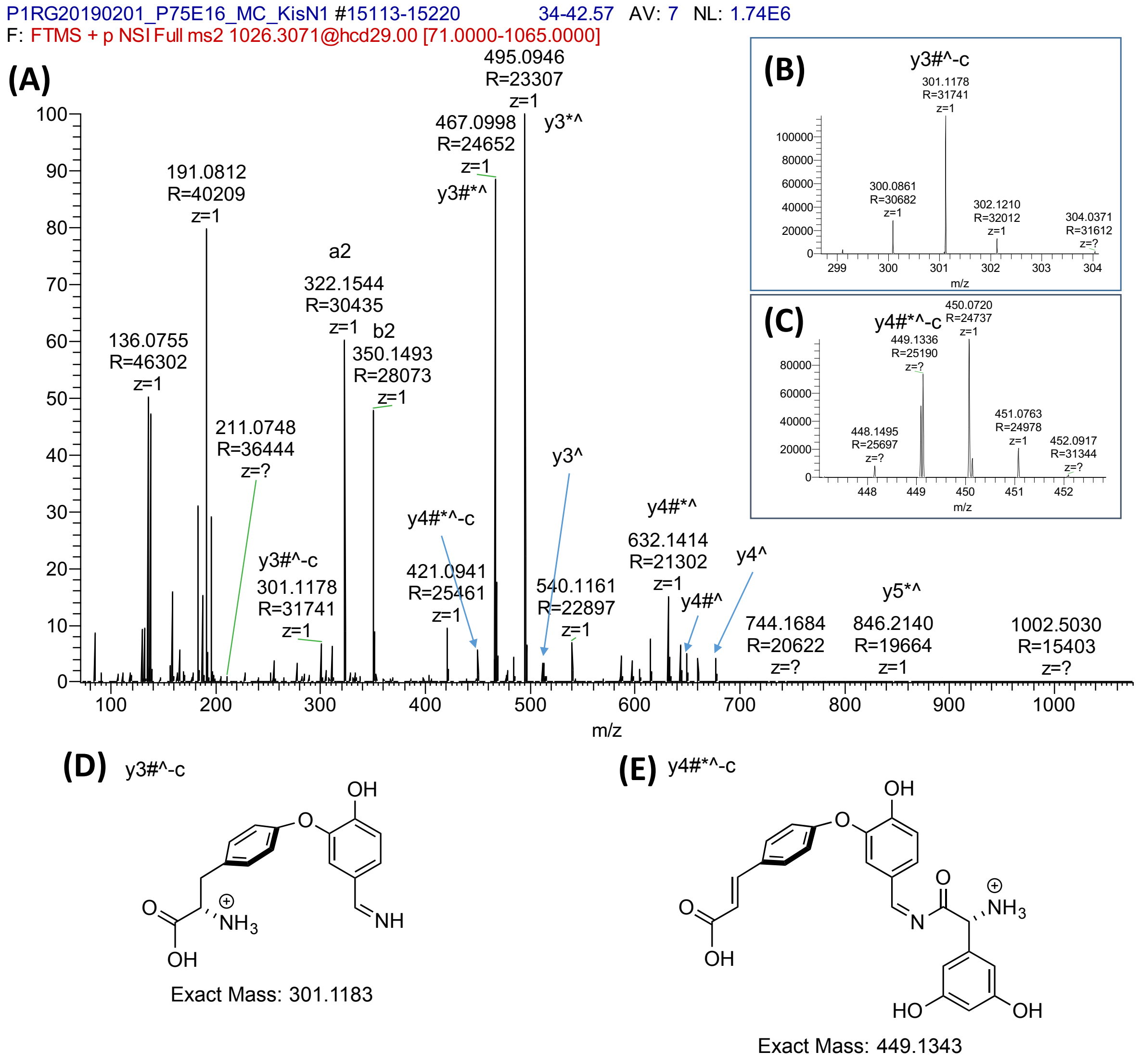

Supplementary Figure 23. MSMS spectrum of monocyclic hexapeptide 1-6 of *A.parvosata ΔkisN*

The *Actinomadura parvosata ΔkisN* mutant produces a monocyclic hexapeptide 1-6 with an Hpg4-Tyr6 crosslink, characterised by the presence of intact y3 to y5 and the absence of y2. Additionally, y3 can lose the chloroHpg at position 5 together with a CO group. (A) MSMS spectrum of the monocyclic hexapeptide with a mass of 1026.3071 $[M+H]^+$. (B) HRMS and (D) chemical structure of fragment y3#^-c (mass 301.1183 $[M+H]^+$). (C) HRMS and (E) chemical structure of fragment y4#*^-c (mass 449.1343 Da). Both fragments indicate that the crosslink is between Hpg4-Tyr6 in the monocyclic hexapeptide of *Actinomadura parvosata ΔkisN* mutant. Symbols: Y tyrosine; W tryptophan; h Hpg; d Dpg; c chloroHpg; # CO loss; * NH_3 loss; ^ crosslink (H_2 loss); The b ions extend from the N-terminus, and y ions extend from C-terminus. Fragmentation ions follow standard nomenclature for such experiments, see [3,4].

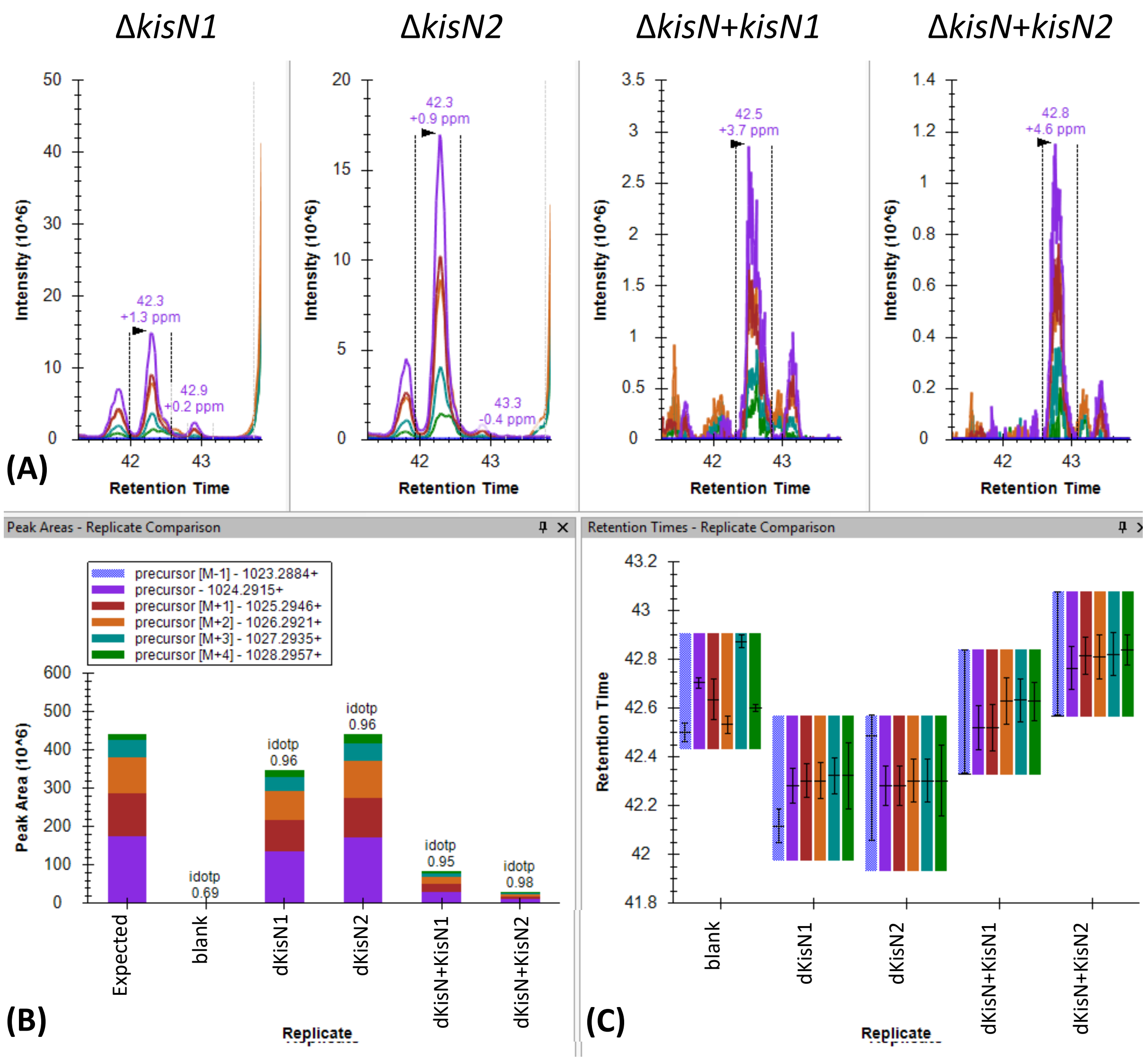

Supplementary Figure 24. Production of kistamicin bicyclic hexapeptide 1-6

Two different mutants (1 + 2) of *Actinomadura parvosata* Δ*kisN* and the complemented mutant Δ*kisN* + *kisN* (1+2) were extracted and analysed by HRMS. The kistamicin bicyclic hexapeptide (1024.2915 $[M+H]^+$) and its isotopic peaks from different producer strains were analysed. Isotopic patterns are important to identify the correct kistamicin species. The purple colour indicates the $[M+H]^+$ isotope, with other colours represent different isotopes (blue -1, red +1, orange +2, cyan +3, green +4), respectively. (A) Extracted ion chromatogram of the respective compound in the different mutants. (B) Integrated peak areas of the respective compound peak in comparison to the expected (calculated) isotope contribution between 39 – 45 min; this is normalised to the intensity of the highest intensity peak in the different strains for comparison. (C) Retention times of the respective kistamicin intermediates and the peak width of these species. The bicyclic hexapeptide is present in significant amounts in the *Actinomadura parvosata* Δ*kisN* mutants. Complementation of these mutants with a plasmid containing the *kisN* gene then leads to a large reduction of this intermediate that is explained by the functional biosynthetic route in this case leading to production of kistamicin A.

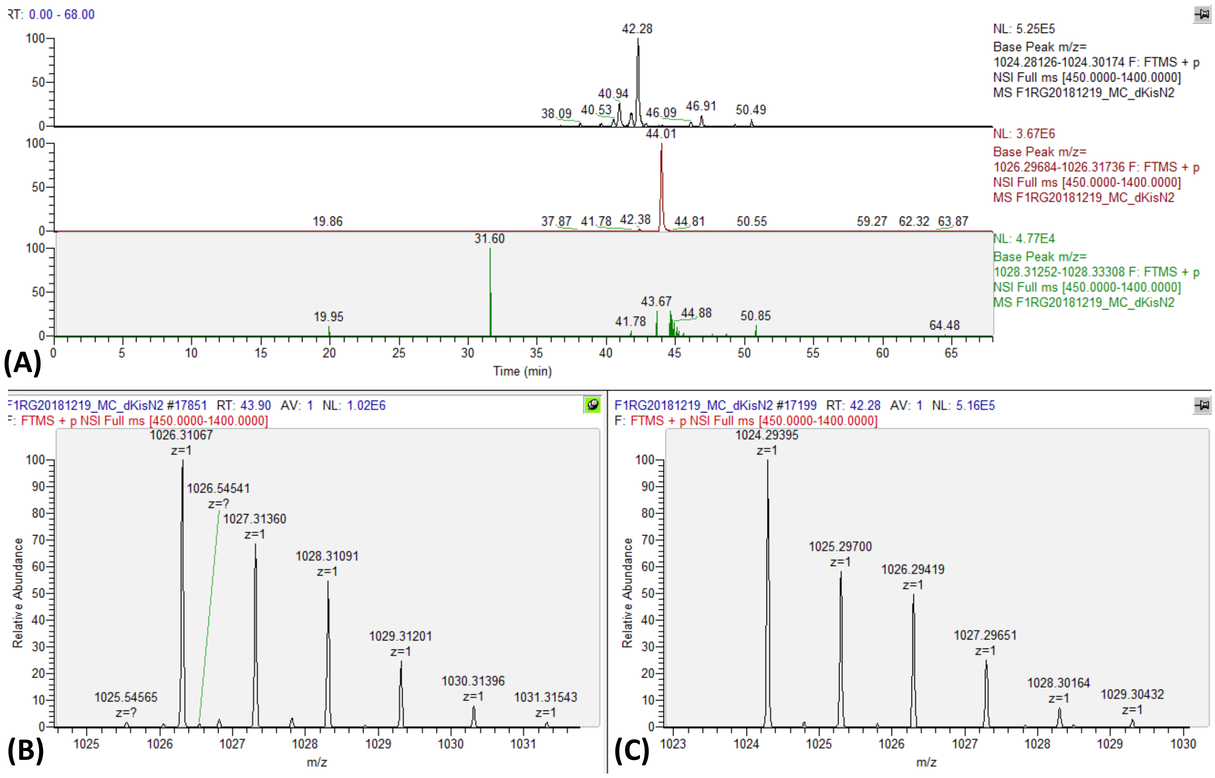

Supplementary Figure 25. Production of bicyclic hexapeptide in *Actinomadura parvosata ΔkisN*

(A) Extracted ion chromatograms for bicyclic kistamicin hexapeptide (1-6) 1024.2915 $[M+H]^+$ (black), monocyclic hexapeptide 1026.3071 $[M+H]^+$ (red) and linear hexapeptide 1028.3228 $[M+H]^+$ (green) with 5 ppm tolerance from the *Actinomadura parvosata ΔkisN* mutant. The mutant produces several bicyclic hexapeptides 1-6, indicated by several peaks with the mass of 1024.2915 $[M+H]^+$ shown in black. The HRMS spectrum of (B) monocyclic hexapeptide at 43.9 min and (C) bicyclic hexapeptide at 42.28 min.

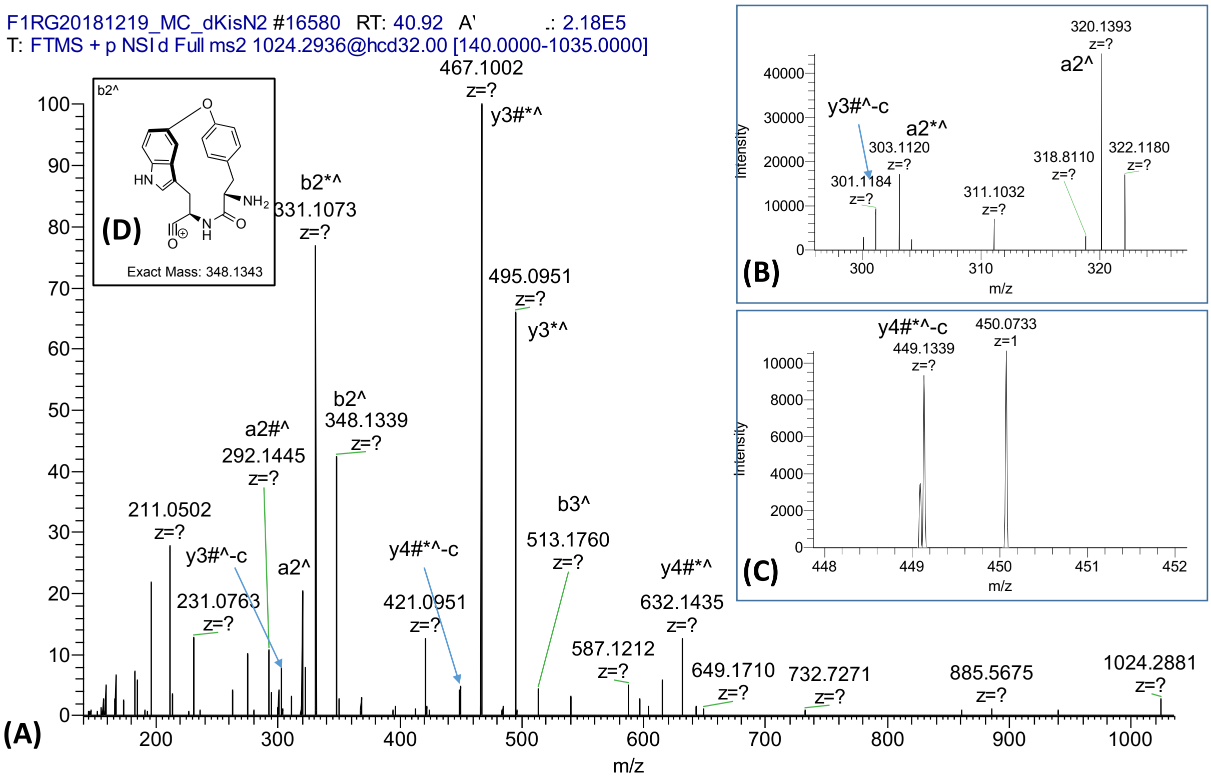

Supplementary Figure 26. MSMS spectrum of one bicyclic hexapeptide in *A. parvosata ΔkisN*

The *Actinomadura parvosata ΔkisN* mutant produces several bicyclic hexapeptides 1-6 as shown in Supplementary Figure 25. (A) MSMS spectrum of the bicyclic hexapeptide at 40.92 min. It demonstrates a Tyr1-Trp2 and Hpg4-Tyr6 crosslinks. The Hpg4-Tyr6 crosslink is characterised by the presence of intact y3 and y4 with crosslinks and the absence of y2. Additionally, y3 and y4 can lose the chloroHpg at position 5 together with a CO (and NH_3) group. The fragmentation pattern shows again the m/z: y3#^-c = 301.1183 (B) and y4#*^-c = 449.1343 (C) like in the monocyclic hexapepetide (see Supplementary Figure 23). The presence of a b2 ion with a crosslink (b2^) and related ions confirms the crosslink occurs within the N-terminal two residues (Tyr1-Trp2). Fragment b2^ indicates the presence of a crosslink between Tyr1-Trp2 in the one of the bicyclic forms of the hexapeptide produced by the *Actinomadura parvosata ΔkisN* mutant; the exact position of the crosslink is not clear. MSMS of other bicyclic species indicate the common presence of Tyr1 in these different links, including Tyr1-Dpg3 (data not shown). (D) Chemical structure of b2^. Symbols: Y tyrosine; W tryptophan; h Hpg; d Dpg; c chloroHpg; # CO loss; * NH_3 loss; ^ crosslink (H_2 loss); The b ions extend from the N-terminus, and y ions extend from C-terminus. Fragmentation ions follow standard nomenclature for such experiments, see [3,4].

4: Kistamicin native 15-membered A-*O*-B Ring

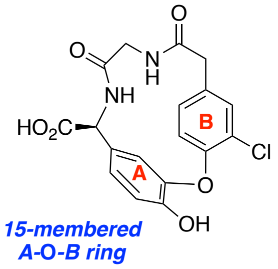

E_{rel} = 0 kcal/mol
G_{rel} = 0 kcal/mol

5: Kistamicin 13-membered AB ring analogue

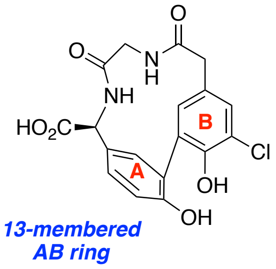

E_{rel} = -11.3 kcal/mol
G_{rel} = -9.7 kcal/mol

6: Kistamicin 12-membered AB ring analogue

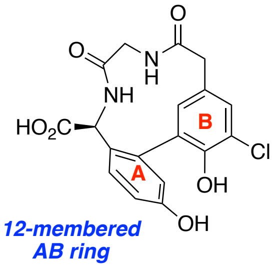

E_{rel} = -10.1 kcal/mol
G_{rel} = -9.3 kcal/mol

Supplementary Figure 27. Kistamicin with different A-B ring crosslinking containing Hpg-7
Comparison of the potential energies and free energies of the kistamicin 15-membered A-*O*-B ring relative to those of 13- and 12-membered AB ring analogues. Source data are provided as a Source Data file.

7: Kistamicin native 16-membered C-*O*-D Ring

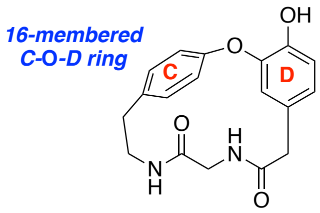

E_{rel} = 0 kcal/mol
G_{rel} = 0 kcal/mol

8: Kistamicin 14-membered CD ring analogue

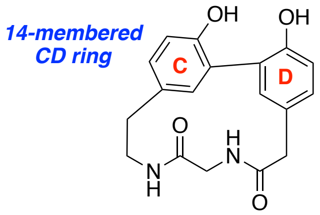

E_{rel} = -4.2 kcal/mol
G_{rel} = -3.0 kcal/mol

Supplementary Figure 28. Kistamicin with different C-D ring crosslinking
Comparison of the potential energies and free energies of the kistamicin 16-membered C-*O*-D ring relative to those of a 14-membered CD ring analogue. Source data are provided as a Source Data file.

9: Model pekiskomycin aglycone with native 12-membered AB ring

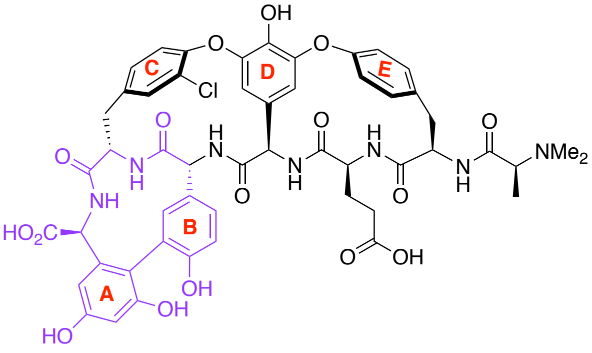

12-membered A-B ring

E_{rel} = 0 kcal/mol

10: Pekiskomycin analogue with 14-membered A-*O*-B ring

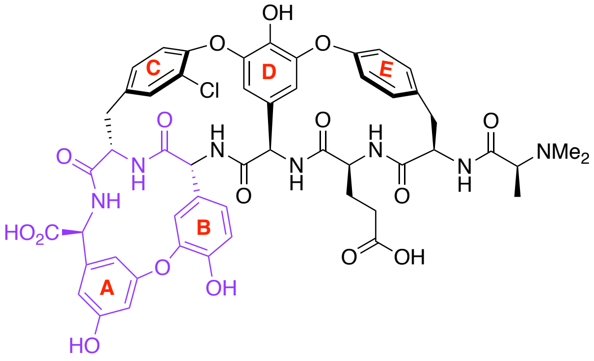

14-membered A-O-B ring

E_{rel} = 2.7 kcal/mol

Supplementary Figure 29. Kistamicin with different A-B ring crosslinking containing Dpg-7

Comparison of the potential energies of pekiskomycin (modelled as **9**), containing the native 12-membered AB ring, relative to the pekiskomycin analogue **10** containing a 14-membered A-*O*-B ring. Source data are provided as a Source Data file.

11: Diphenyl ether

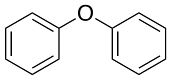

E_{rel} = 0 kcal/mol
G_{rel} = 0 kcal/mol

12: 2-Hydroxybiphenyl

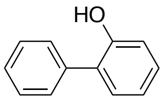

E_{rel} = -7.6 kcal/mol
G_{rel} = -6.3 kcal/mol

Supplementary Figure 30. Diphenyl ether vs. 2-hydroxybiphenyl

Relative potential energies and free energies of diphenyl ether **11** and 2-hydroxybiphenyl **12**. Source data are provided as a Source Data file.

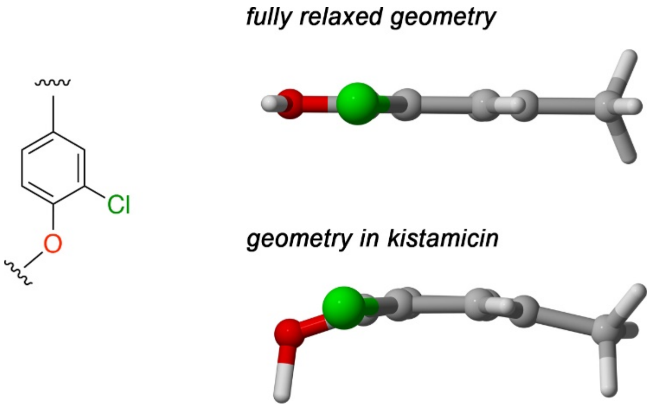

Supplementary Figure 31. Geometry of the A-*O*-B ring in kistamicin

Comparison of the geometry of the A-*O*-B ring in kistamicin relative to the fully relaxed geometry. The substituents on the ether oxygen and α-carbon have been modelled as hydrogens.

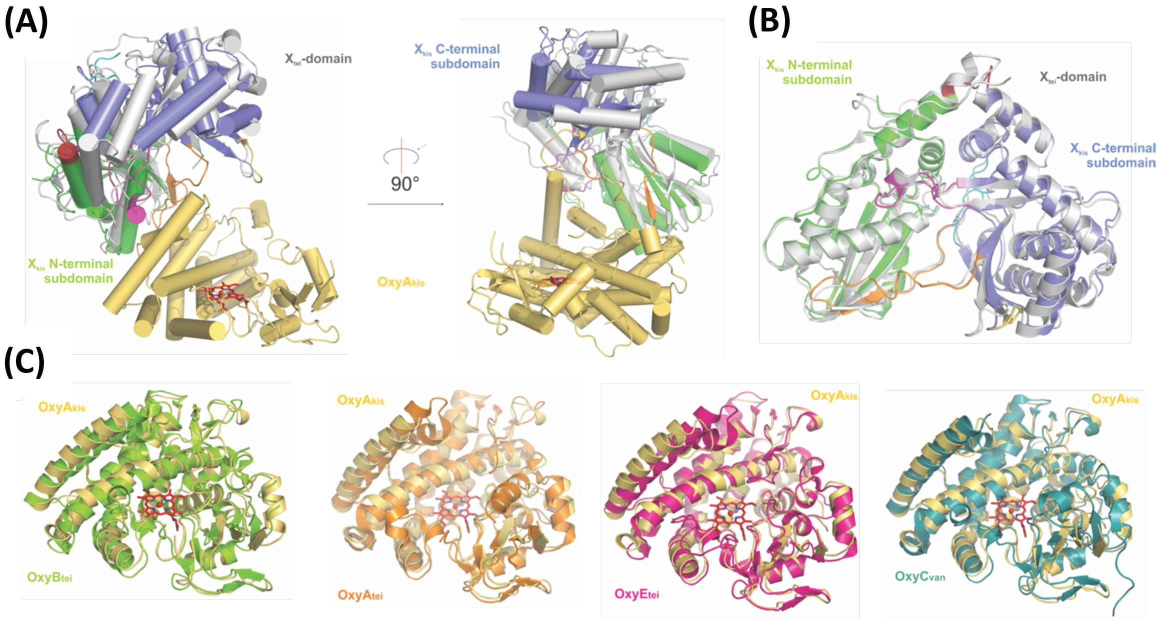

Supplementary Figure 32. Structural comparisons of the OxyA$_{kis}$/X$_{kis}$ complex

(A) Overlay of the Oxy enzyme in the OxyA$_{kis}$/X$_{kis}$ complex with the OxyB$_{tei}$/X$_{tei}$ complex showing only the position of X$_{tei}$ relative to the OxyA$_{kis}$/X$_{kis}$ complex (X$_{tei}$ shown in grey, OxyA$_{kis}$/X$_{kis}$ complex coloured as in Figure 3, helices shown as cylinders). (B) Overlay of the X domains from the OxyA$_{kis}$/X$_{kis}$ complex and OxyB$_{tei}$/X$_{tei}$ complexes (coloured as in panel (A)). (C) Overlay of Oxy homologues on OxyA$_{kis}$ structure (OxyA$_{kis}$ shown in yellow; from left OxyA$_{tei}$ (coloured green), OxyE$_{tei}$ (coloured orange), OxyB$_{tei}$ (coloured magenta), OxyC$_{van}$ (coloured aquamarine)).

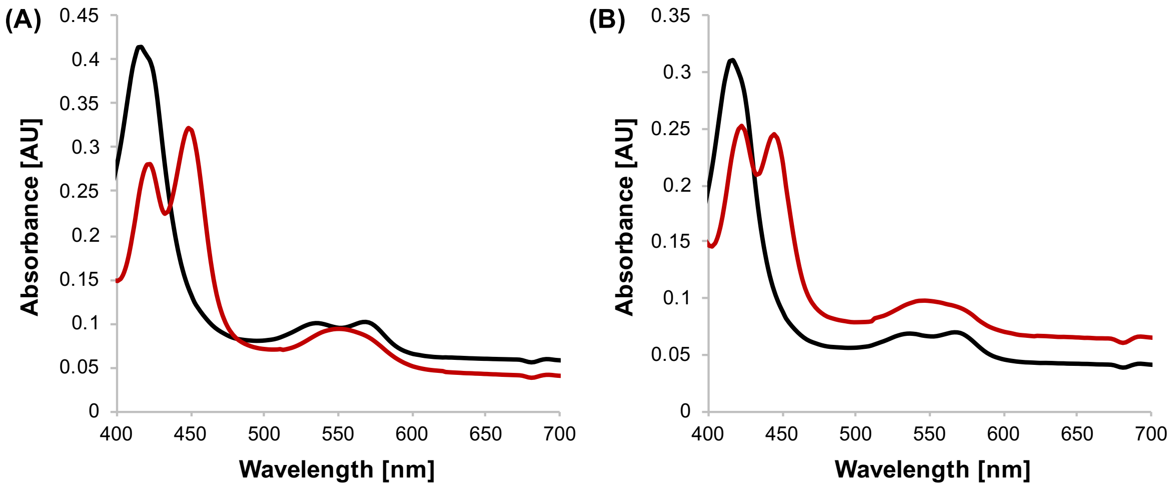

Supplementary Figure 33. CO spectra of OxyA and OxyC

UV/vis absorption spectra (black) and reduced, CO-complex (red) shown for purified $OxyA_{kis}$ (A) and $OxyC_{kis}$ (B). Both proteins are able to be reduced by sodium dithionite treatment that is then subsequently used to generate the reduced CO complex, as is shown by the shift of the absorption maximum from 418 nm to 450 nm.

(A)

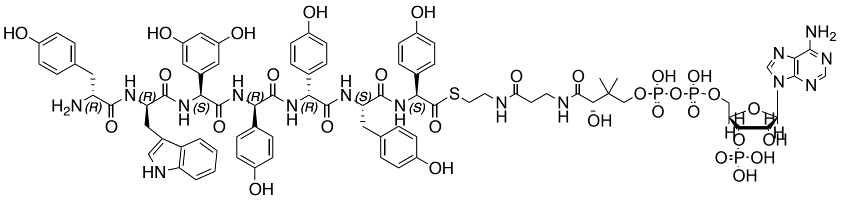

Chemical Formula: $C_{82}H_{92}N_{15}O_{30}P_3S$
Exact Mass: 1891.51
Molecular Weight: 1892.68

(B)

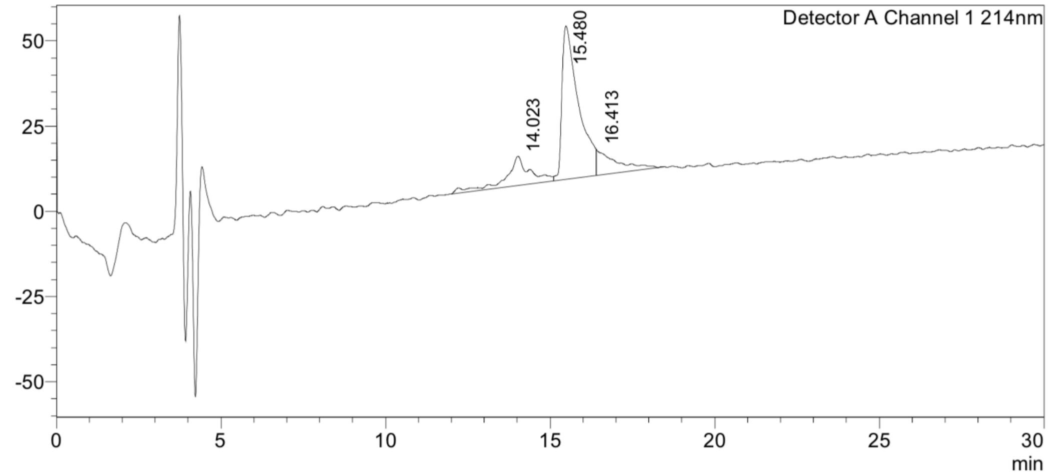

RT: 15.480 min in a gradient of 15-45% in ACN (0.1% FA)

(C)

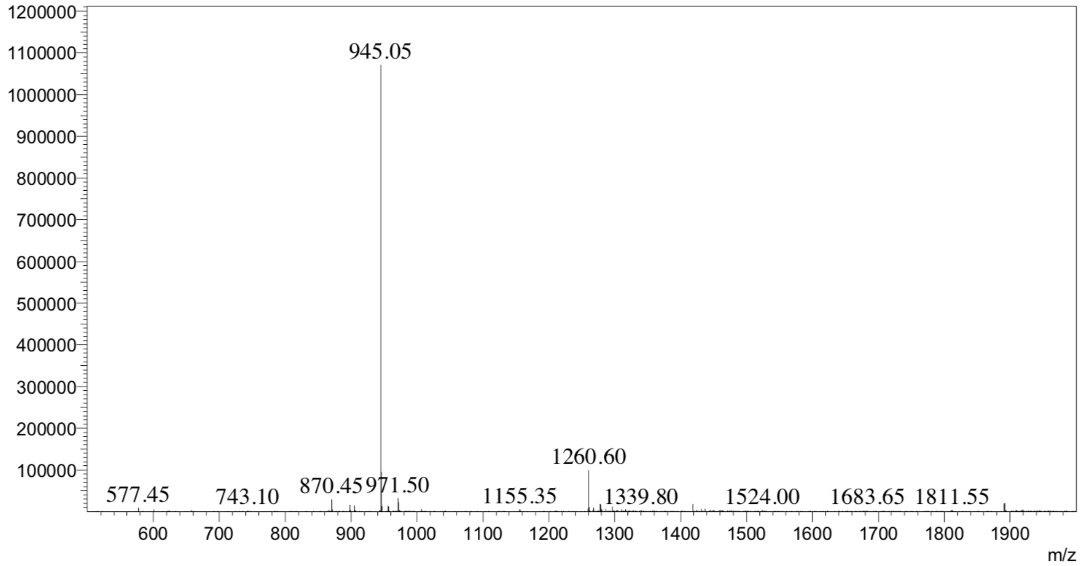

$[M-2H]^{2-}$ / 2 = 945.05 Da; $MW_{calculated}$ = 1892.10 Da

Supplementary Figure 34. Synthesised peptide K1-7-CoA

(A) Structure, (B) HPLC chromatogram and (C) MS spectrum of synthesised peptide K1-7-CoA.

(A)

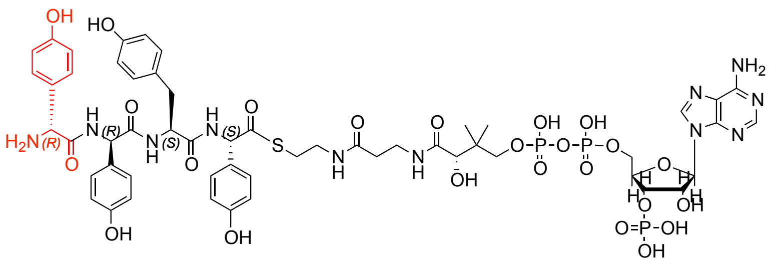

Chemical Formula: $C_{54}H_{66}N_{11}O_{24}P_3S$
Exact Mass: 1377.32
Molecular Weight: 1378.16

(B)

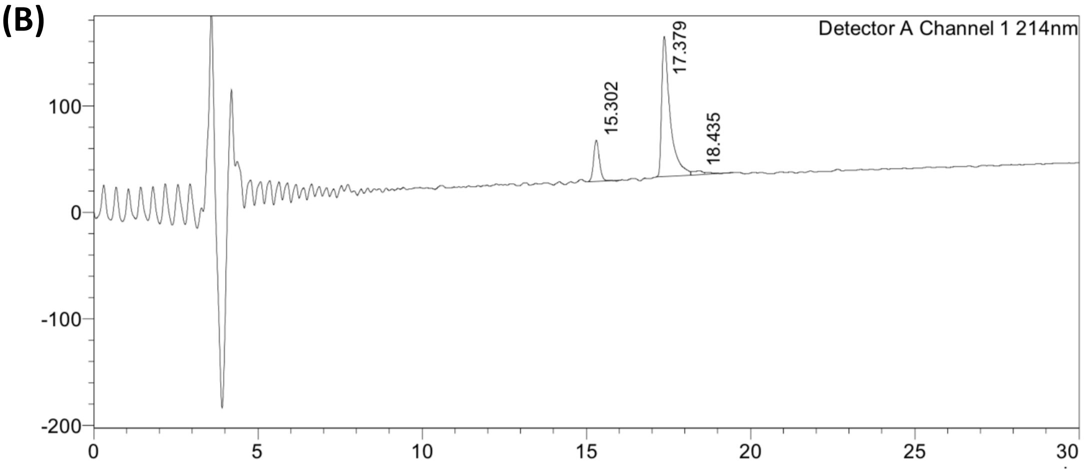

RT: 17.379 min in a gradient of 5-35% in ACN (0.1% FA)

(C)

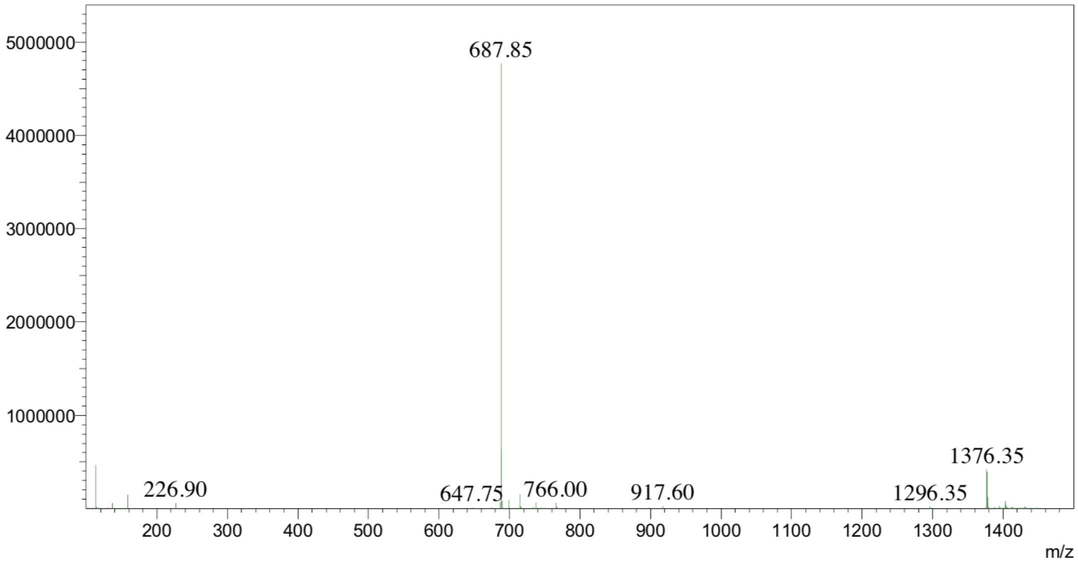

$[M-H]^-$ = 1376.35 Da; $MW_{calculated}$ = 1377.35 Da
$[M-2H]^{2-}$ / 2 = 687.85 Da; $MW_{calculated}$ = 1377.70 Da

Supplementary Figure 35. Synthesised peptide K4-7D-CoA

(A) Structure, (B) HPLC chromatogram and (C) MS spectrum of synthesised peptide K4-7D-CoA. In red, the central Hpg residue, which is synthesised in D- and L-form.

(A)

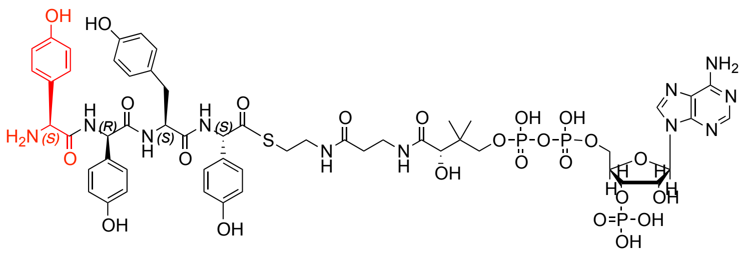

Chemical Formula: $C_{54}H_{66}N_{11}O_{24}P_3S$
Exact Mass: 1377.32
Molecular Weight: 1378.16

(B)

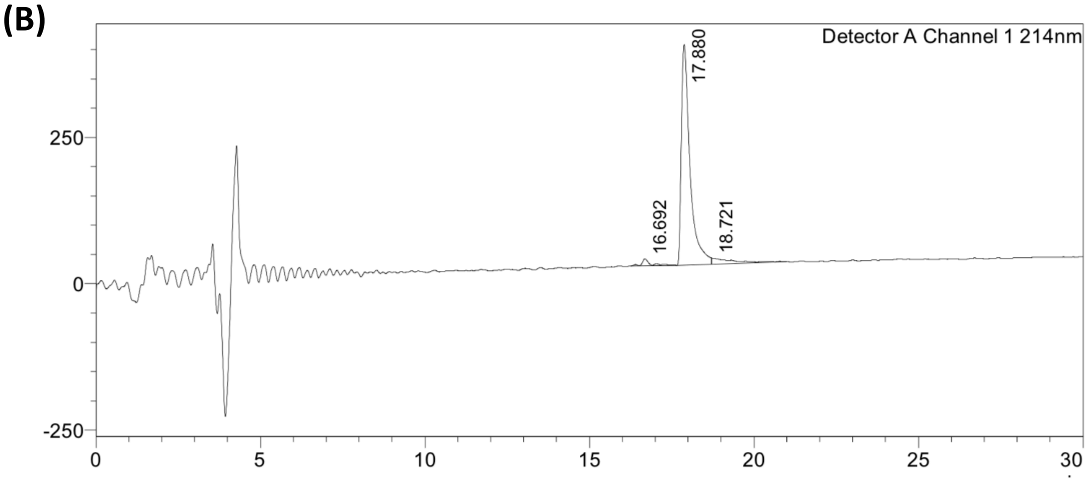

RT: 17.880 min in a gradient of 5-35% in ACN (0.1% FA)

(C)

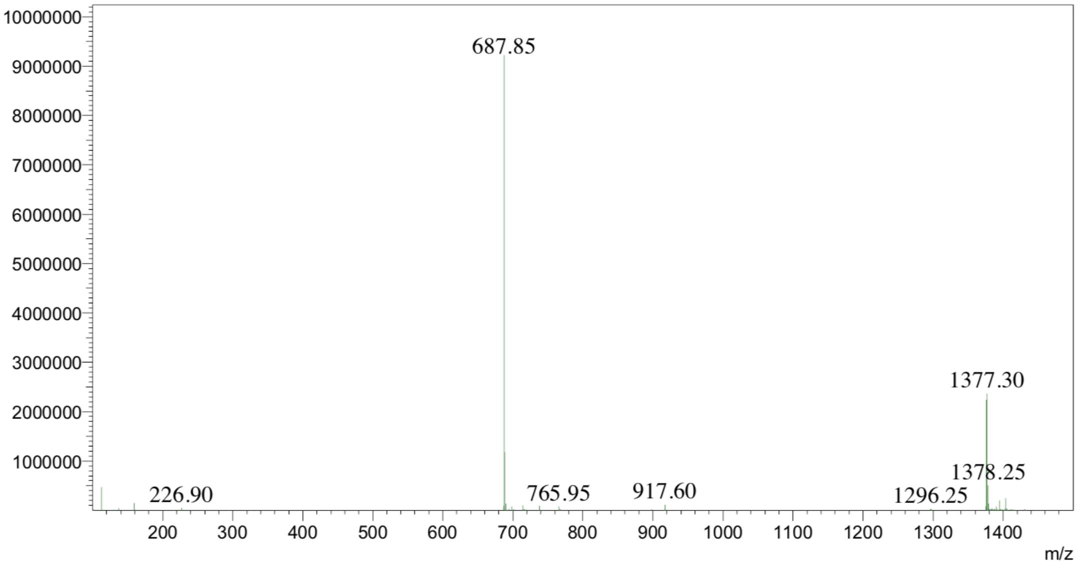

$[M-H]^-$ = 1377.30 Da; $MW_{calculated}$ = 1378.30 Da
$[M-2H]^{2-}$ / 2 = 687.85 Da; $MW_{calculated}$ = 1377.70 Da

Supplementary Figure 36. Synthesised peptide K4-7L-CoA

(A) Structure, (B) HPLC chromatogram and (C) MS spectrum of synthesised peptide K4-7L-CoA. In red, the central Hpg residue, which is synthesised in D- and L-form.

(A)

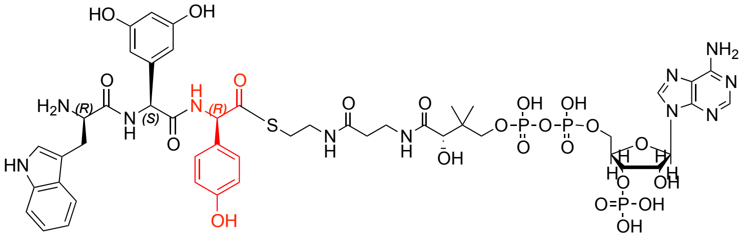

Chemical Formula: $C_{48}H_{60}N_{11}O_{22}P_3S$
Exact Mass: 1267.28
Molecular Weight: 1268.04

(B)

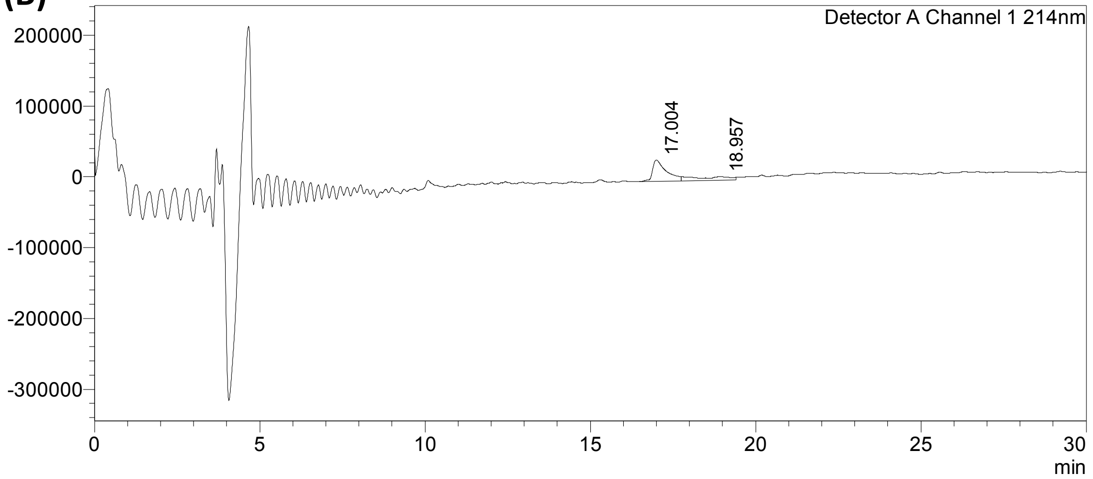

RT: 17.004 min in a gradient of 5-35% in ACN (0.1% FA)

(C)

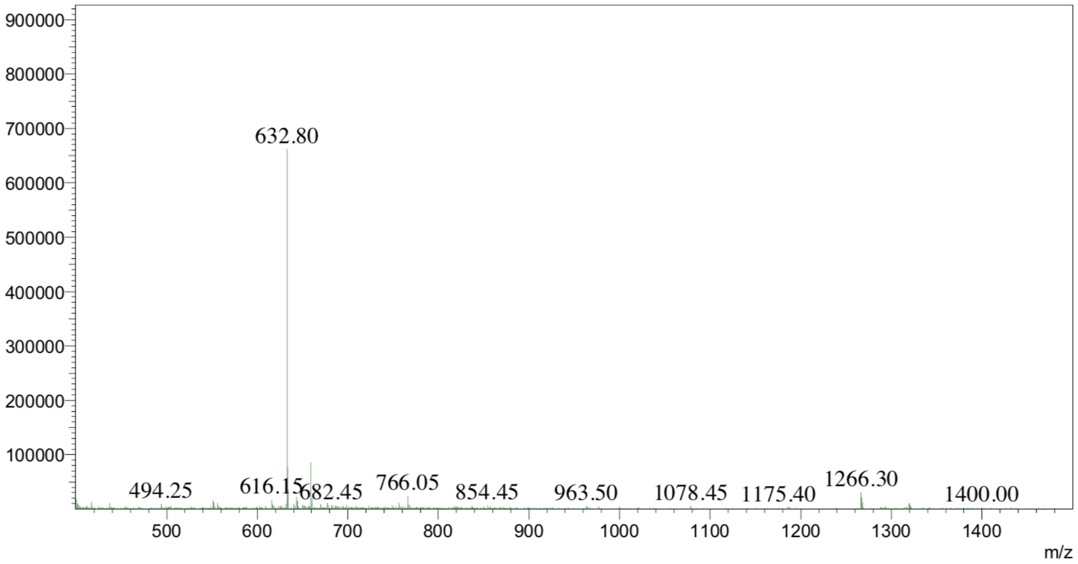

$[M-H]^-$ = 1266.30 Da; $MW_{calculated}$ = 1267.30 Da
$[M-2H]^{2-}$ / 2 = 632.80 Da; $MW_{calculated}$ = 1267.60 Da

Supplementary Figure 37. Synthesised peptide K2-4D-CoA

(A) Structure, (B) HPLC chromatogram and (C) MS spectrum (bottom) of synthesised peptide K2-4D-CoA. In red, the central Hpg residue, which is synthesised in D- and L-form.

(A)

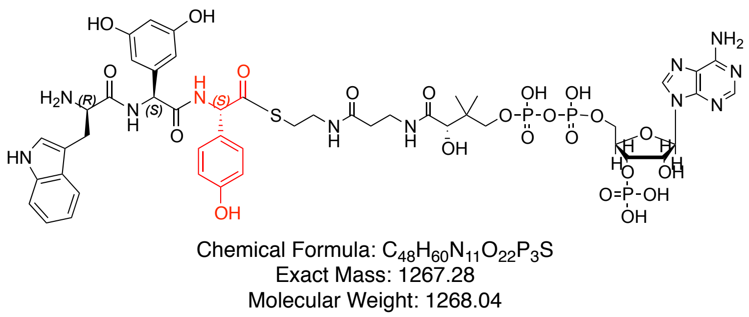

(B)

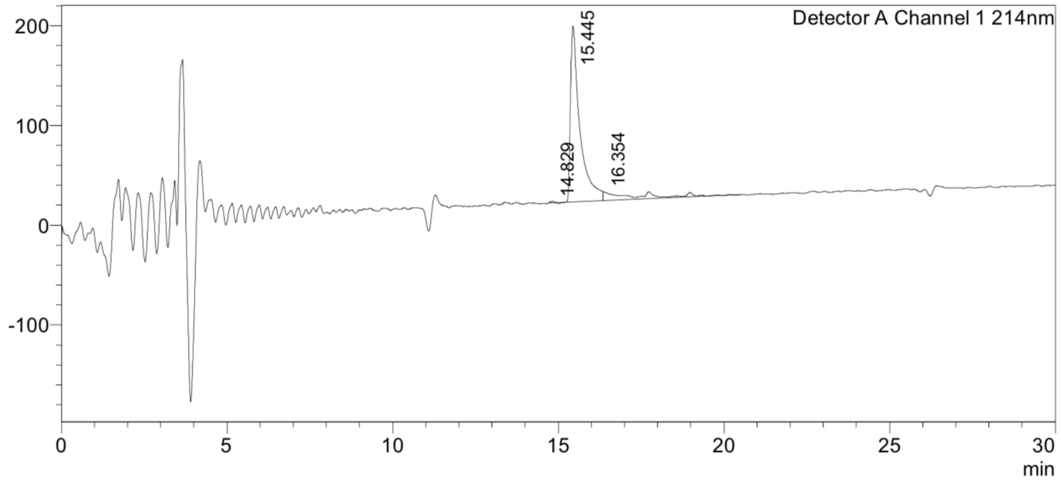

RT: 15.445 min in a gradient of 5-35% ACN (0.1% FA)

(C)

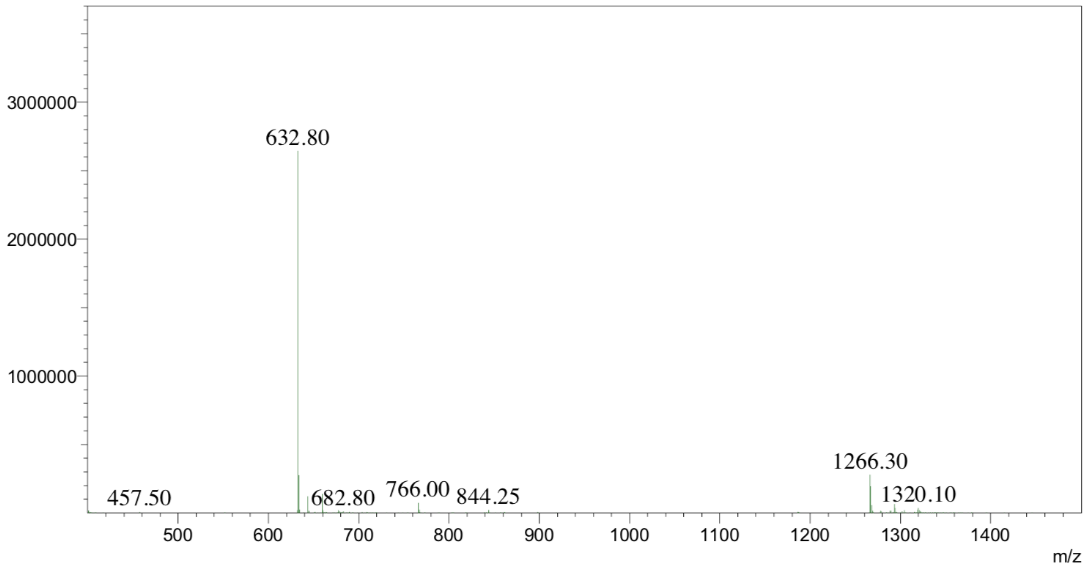

$[M-H]^{-}$ = 1266.30 Da; $MW_{calculated}$ = 1267.30 Da

$[M-2H]^{2-}$ / 2 = 632.80 Da; $MW_{calculated}$ = 1267.60 Da

Supplementary Figure 38. Synthesised peptide K2-4L-CoA

(A) Structure, (B) HPLC chromatogram and (C) MS spectrum (bottom) of synthesised peptide K2-4L-CoA. In red, the central Hpg residue, which is synthesised in D- and L-form.

(A)

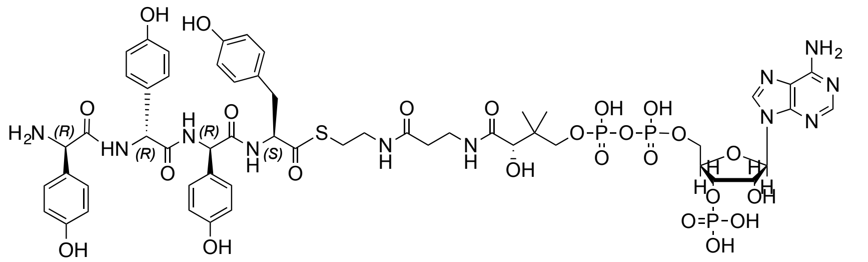

Chemical Formula: $C_{54}H_{66}N_{11}O_{24}P_3S$
Exact Mass: 1377.3216
Molecular Weight: 1378.1563

(B)

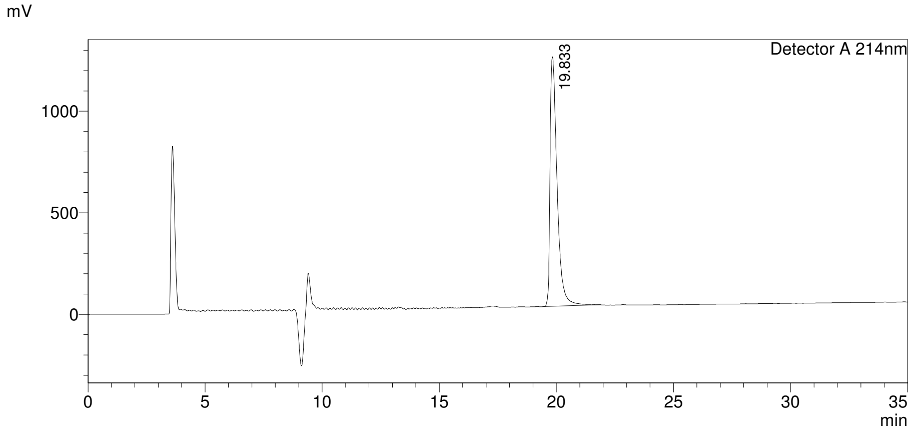

RT: 19.833 min in a gradient 5-5-35% in ACN (0.1% FA)

(C)

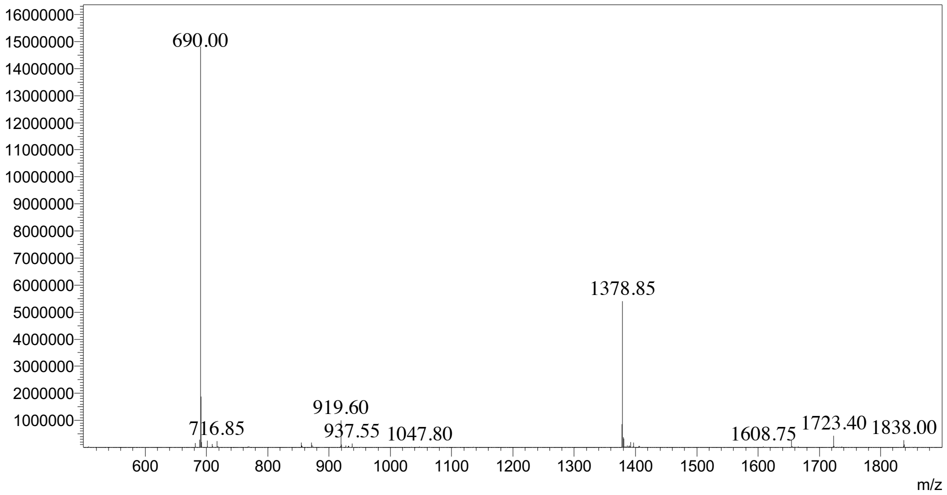

$[M+H]^+$ = 1378.85 Da; $MW_{calculated}$ = 1378.33 Da
$[M+H]^{2+}/2$ = 690.00 Da; $MW_{calculated}$ = 689.67 Da

Supplementary Figure 39. Synthesised peptide K3-6-CoA

(A) Structure, (B) HPLC chromatogram and (C) MS spectrum of synthesised peptide K3-6-CoA.

(A)

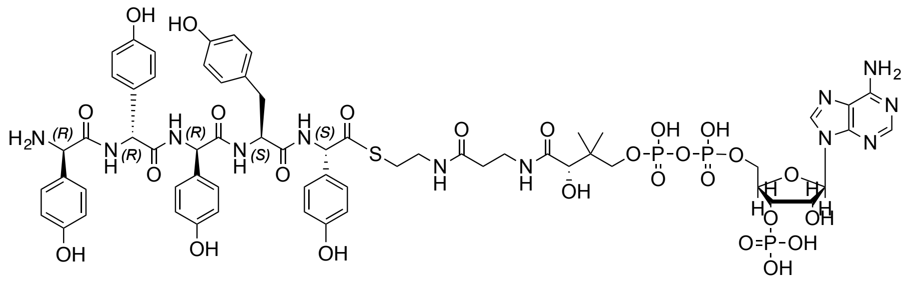

Chemical Formula: $C_{62}H_{73}N_{12}O_{26}P_3S$
Exact Mass: 1526.3693
Molecular Weight: 1527.3053

(B)

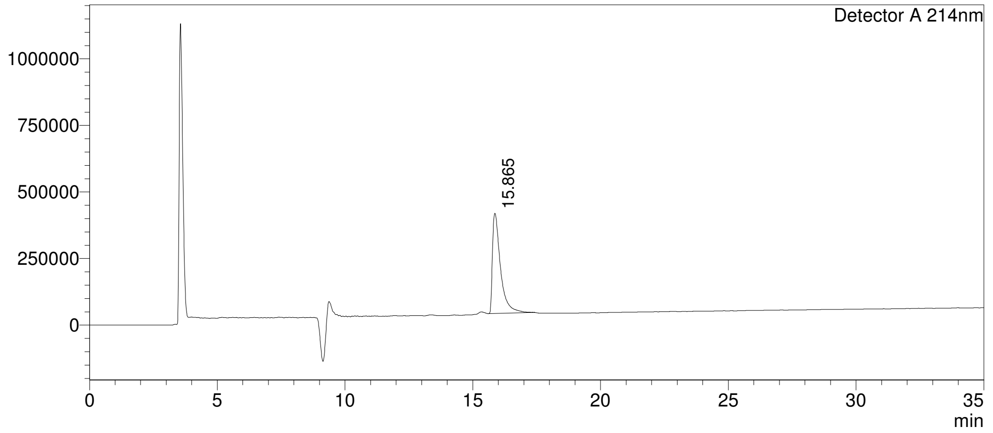

RT: 15.865 min in a gradient 15-15-45% in ACN (0.1% FA)

(C)

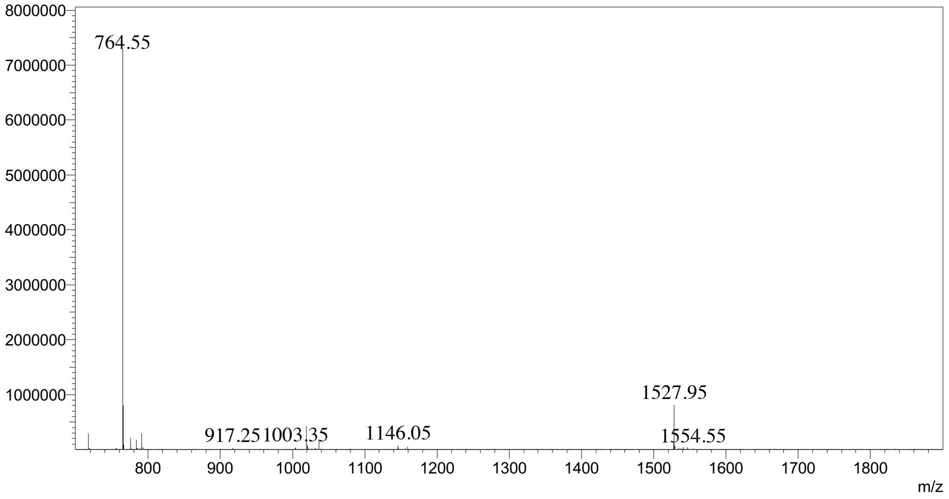

$[M+H]^+$ = 1527.95 Da; $MW_{calculated}$ = 1527.38 Da
$[M+H]^{2+}/2$ = 764.55 Da; $MW_{calculated}$ = 764.19 Da

Supplementary Figure 40. Synthesised peptide K3-7-CoA

(A) Structure, (B) HPLC chromatogram and (C) MS spectrum of synthesised peptide K3-7-CoA.

(A)

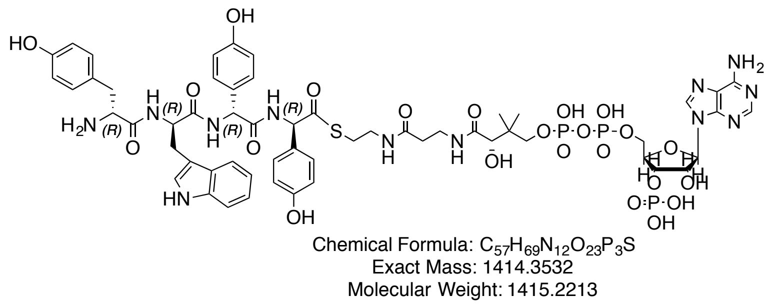

(B)

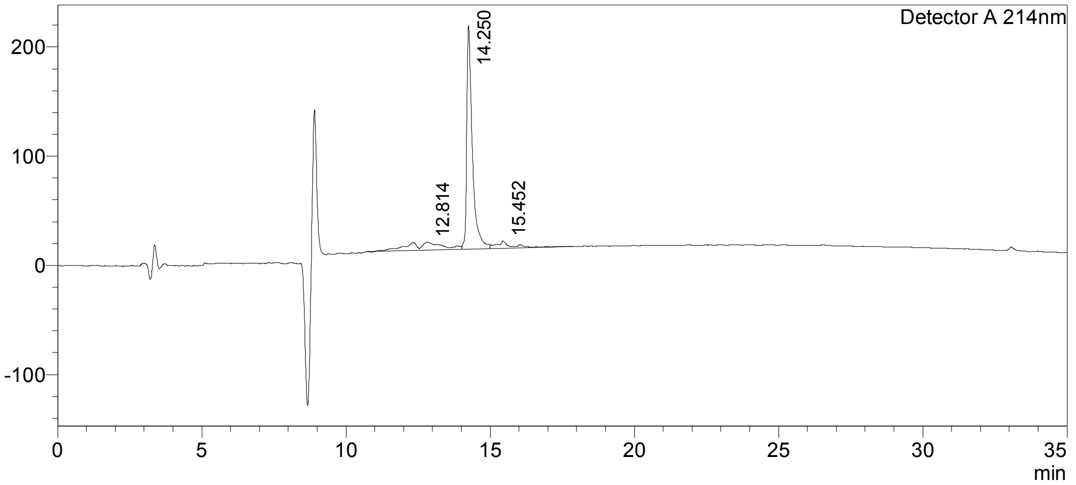

RT: 14.250 min in a gradient of 10-10-40% in ACN (0.1% FA)

(C)

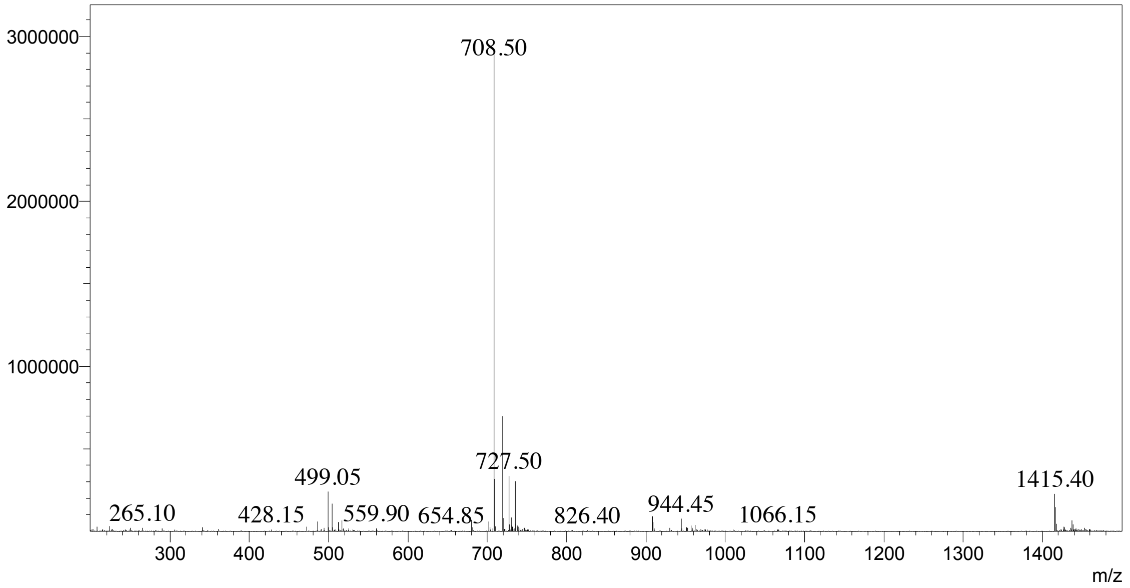

$[M-2H]^{2-}$ / 2 = 708.18 Da; $MW_{calculated}$ = 1414.35 Da

Supplementary Figure 41. Synthesised peptide K1-4-CoA

(A) Structure, (B) HPLC chromatogram and (C) MS spectrum of synthesised peptide K1-4-CoA.

(A)

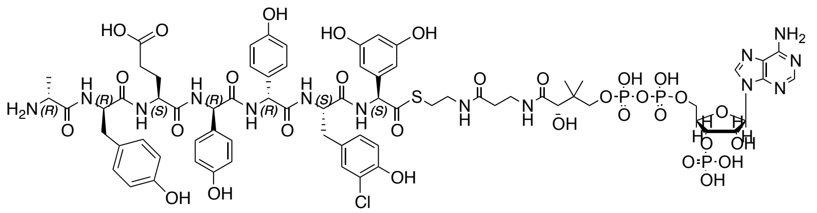

Chemical Formula: $C_{71}H_{86}ClN_{14}O_{31}P_3S$
Exact Mass: 1790.42
Molecular Weight: 1791.96

(B)

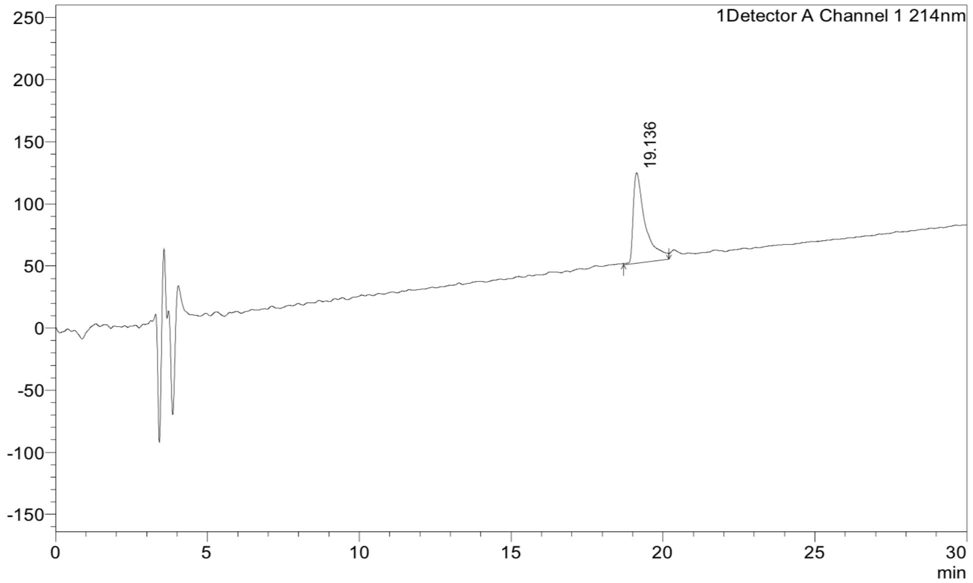

RT: 19.136 min in a gradient of 10-40% in ACN (0.1% FA)

(C)

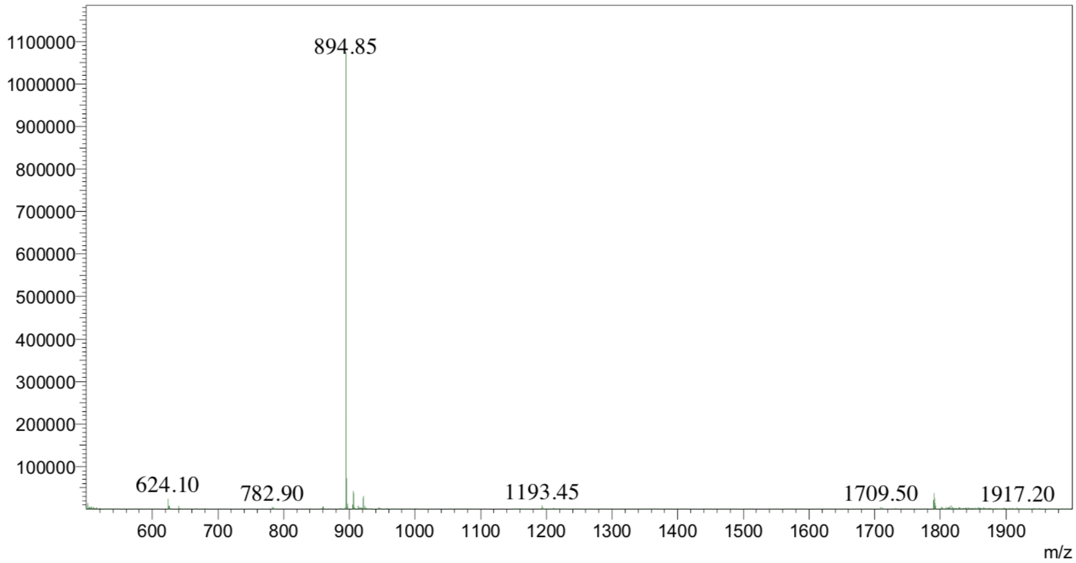

$[M-2H]^{2-}$ / 2 = 894.85 Da; $MW_{calculated}$ = 1791.70 Da

Supplementary Figure 42. Synthesised peptide P1-7-CoA

(A) Structure, (B) HPLC chromatogram and (C) MS spectrum of synthesised peptide P1-7-CoA.

(A)

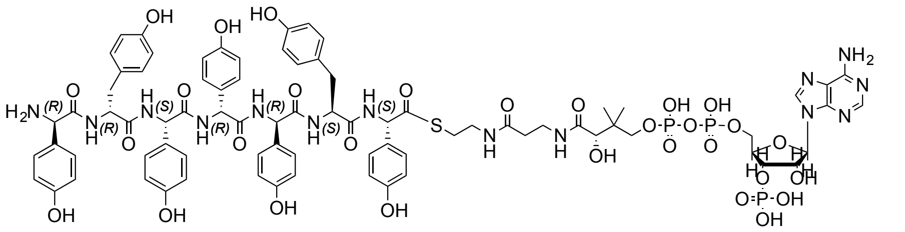

Chemical Formula: $C_{79}H_{89}N_{14}O_{30}P_3S$
Exact Mass: 1838.4803
Molecular Weight: 1839.6303

(B)

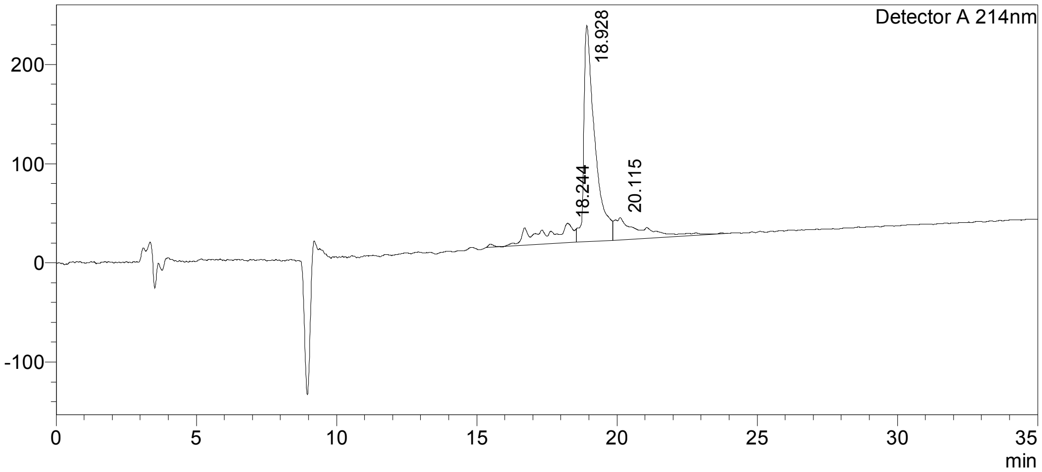

RT: 19.133 min in a gradient 15-15-45% in ACN (0.1% FA)

(C)

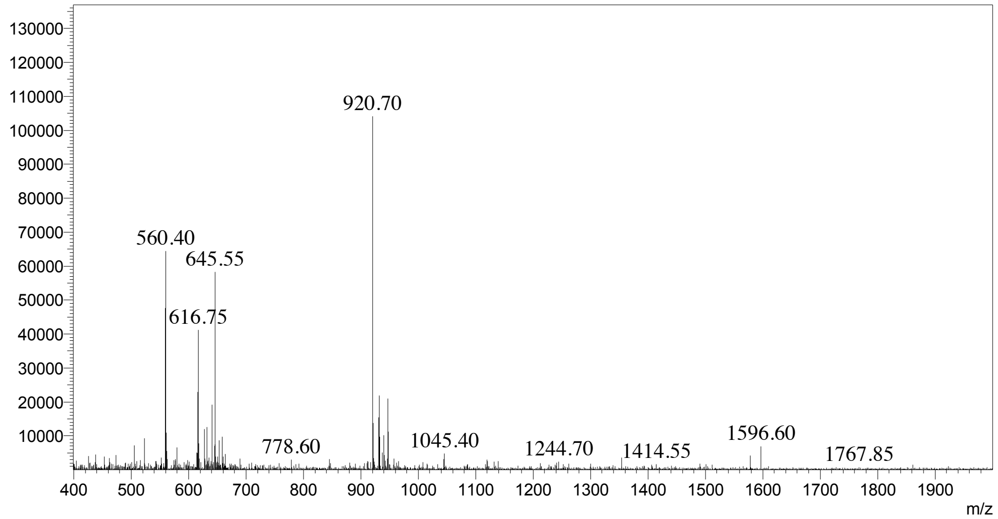

$[M+H]^+$ = 1839.42 Da; $MW_{calculated}$ = 1839.48 Da
$[M+H]^{2+}/2$ = 920.7 Da; $MW_{calculated}$ = 920.25 Da

Supplementary Figure 43. Synthesised peptide T1-7-CoA

(A) Structure, (B) HPLC chromatogram and (C) MS spectrum of synthesised peptide T1-7-CoA.

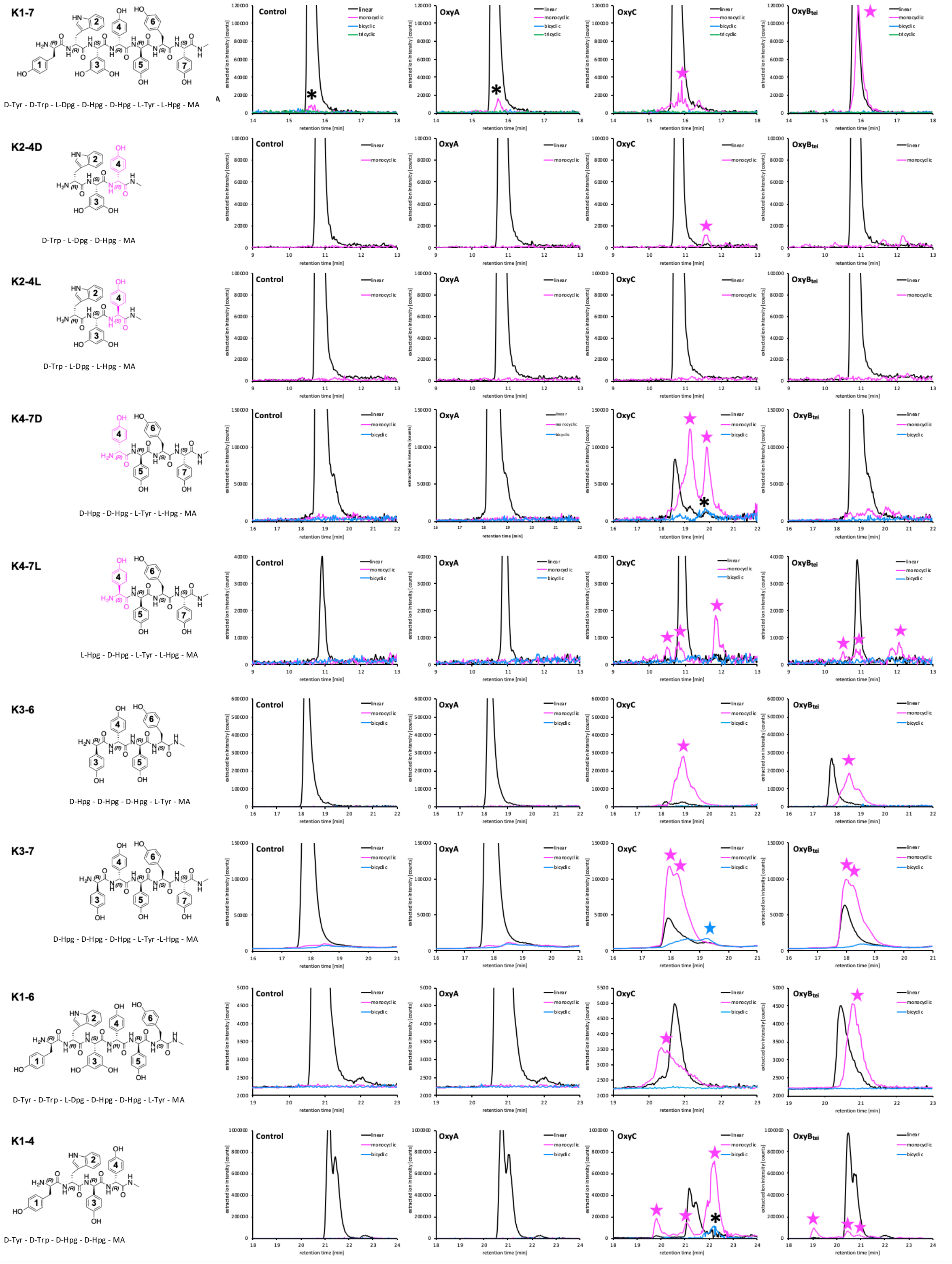

Supplementary Figure 44. Overview of extracted ion chromatograms of the Oxy-catalysed turnover Extracted ions from analysis in positive mode $[M+H]^+$ of different kistamicin peptides shown with high magnification. Peaks with monocyclic mass are indicated by a pink star, peaks with bicyclic mass by a blue star. *peaks were subtracted from the calculation because they lie under the main peaks. MA, methylamine, linear mass (black), monocyclic mass (pink), bicyclic mass (blue) and tricyclic mass (green). Enzyme components present in each reaction are indicated adjacent to the Y-axis in each trace within the figure.

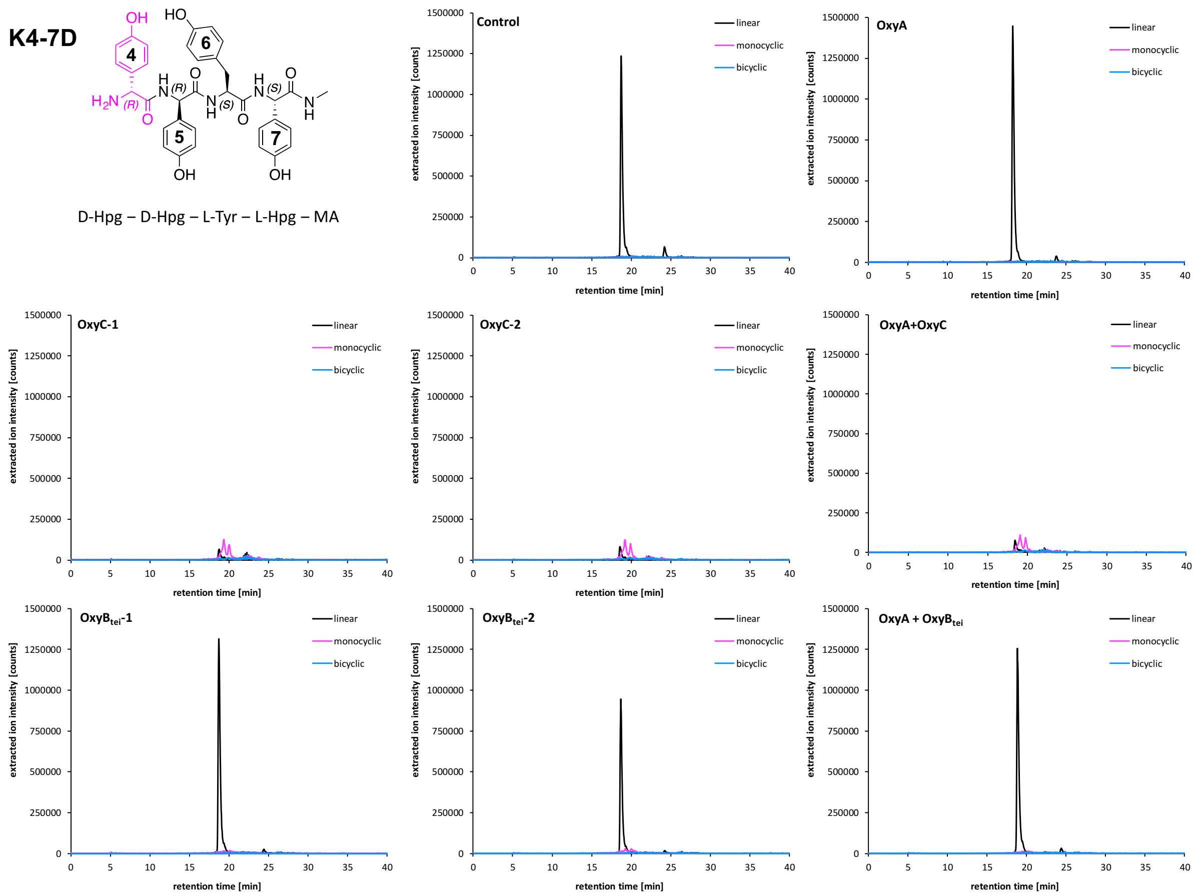

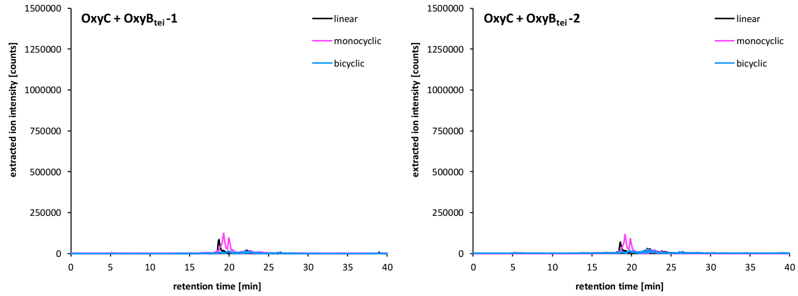

Peptide	$[M+H^+]^+$
linear	642.26
monocyclic	640.24
bicyclic	638.22

Supplementary Figure 45. XIC of the Oxy-catalysed turnover of kistamicin tetrapeptides K4-7D
Extracted ions from analysis in positive mode $[M+H]^+$ of linear tetrapeptide K4-7D methylamide (MA) with a mass of 642.2 Da (black), monocyclic methylamide product with the mass of 640.2 Da (pink) and bicyclic methylamide product with the mass of 638.2 Da (blue) following methylamine cleavage. Enzyme components present in each reaction are indicated adjacent to the Y-axis in each trace within the figure. Source data are provided as a Source Data file.

K4-7L

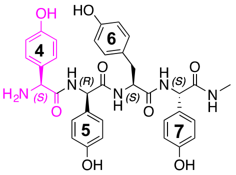

L-Hpg – D-Hpg – L-Tyr – L-Hpg – MA

Peptide	[M+H⁺]⁺
linear	642.26
monocyclic	640.24
bicyclic	638.22

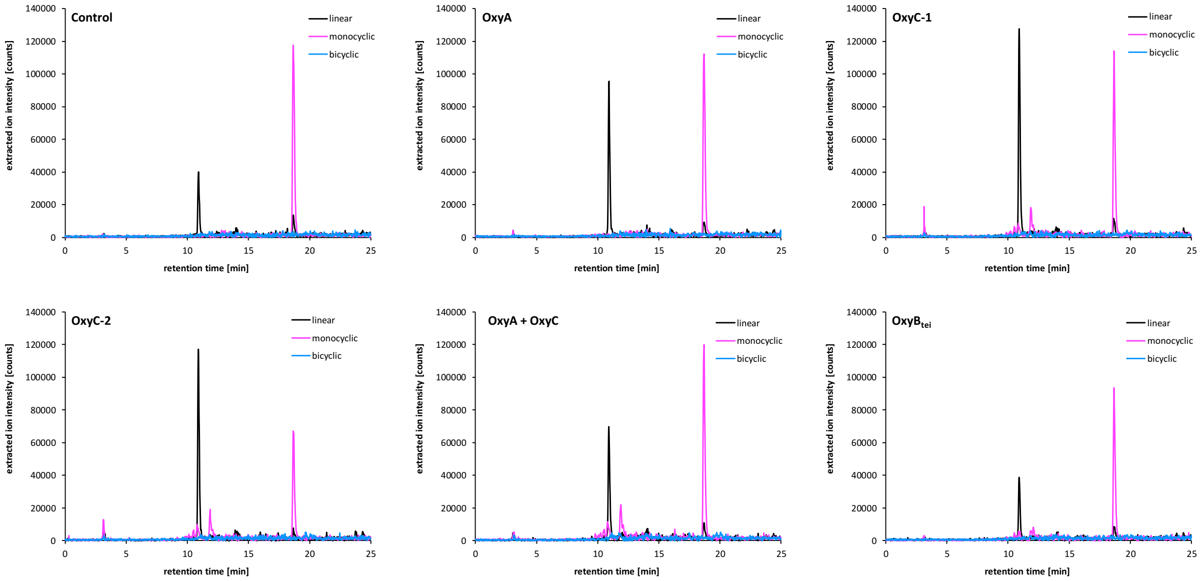

Supplementary Figure 42. XIC of the Oxy-catalysed turnover of kistamicin tetrapeptides K4-7L
Extracted ions from analysis in positive mode $[M+H]^+$ of linear tetrapeptide K4-7L methylamide (MA) with a mass of 642.2 Da (black), monocyclic methylamide product with the mass of 640.2 Da (pink) and bicyclic methylamide product with the mass of 638.2 Da (blue) following methylamine cleavage. Enzyme components present in each reaction are indicated adjacent to the Y-axis in each trace within the figure. Source data are provided as a Source Data file.

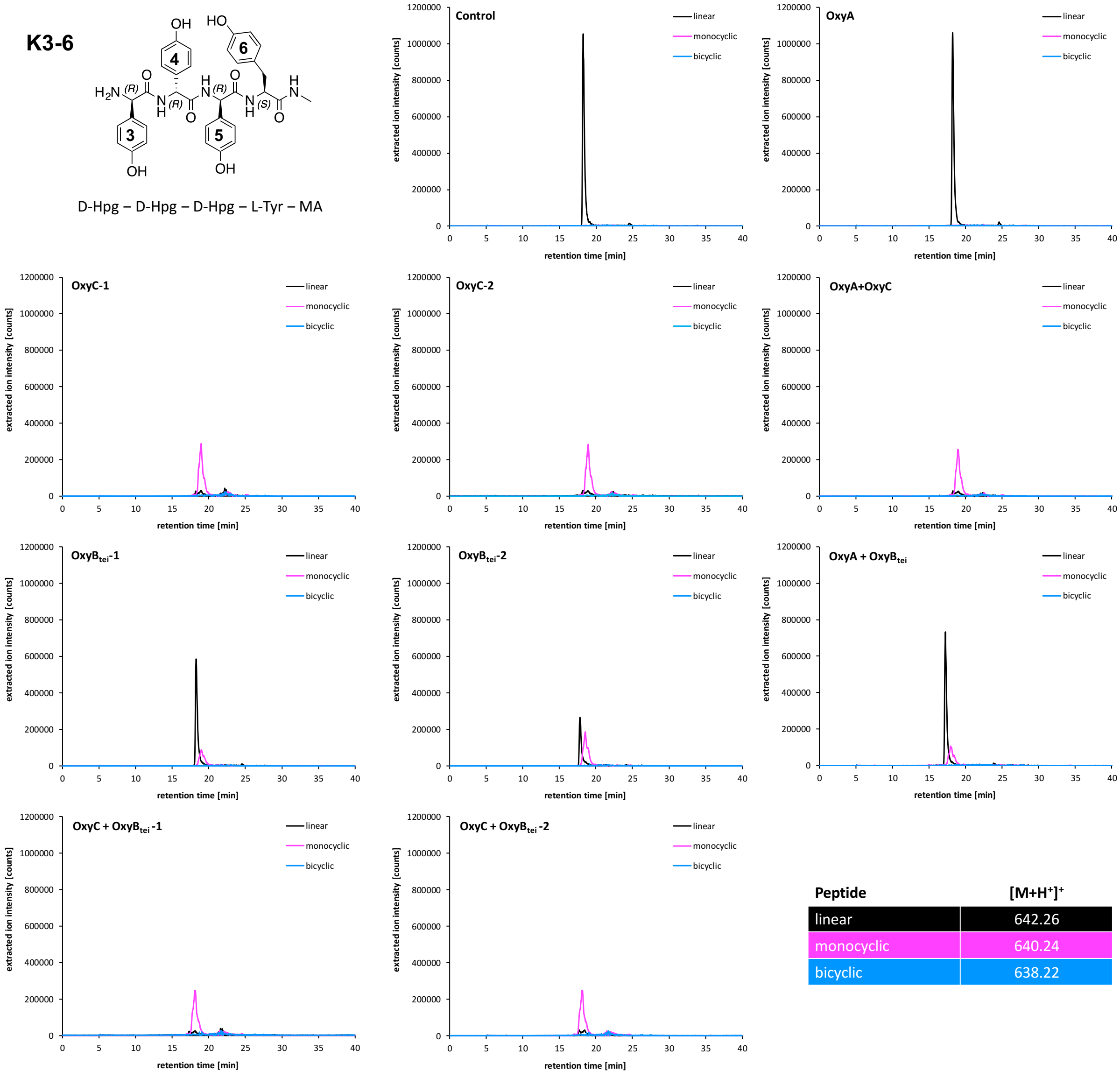

Peptide	$[M+H^{+}]^{+}$
linear	642.26
monocyclic	640.24
bicyclic	638.22

Supplementary Figure 47. XIC of the Oxy-catalysed turnover of kistamicin tetrapeptide K3-6
Extracted ions from analysis in positive mode $[M+H]^{+}$ of linear tetrapeptide K3-6 methylamide with a mass of 642.2 Da (black), monocyclic methylamide (MA) product with the mass of 640.2 Da (pink) and bicyclic methylamide product with the mass of 638.2 Da (blue) following methylamine cleavage. Enzyme components present in each reaction are indicated adjacent to the Y-axis in each trace within the figure. Source data are provided as a Source Data file.

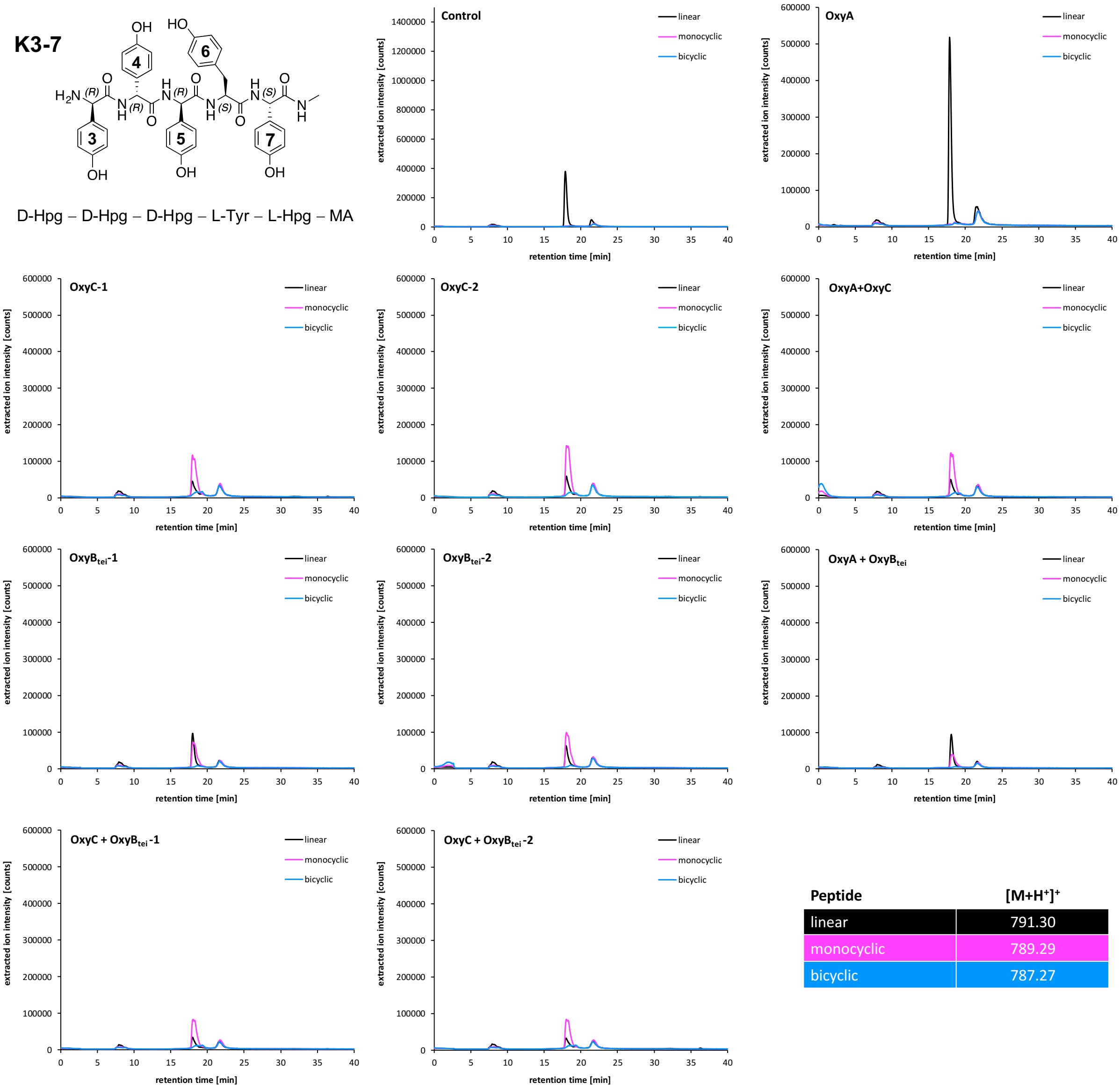

Peptide	$[M+H^+]^+$
linear	791.30
monocyclic	789.29
bicyclic	787.27

Supplementary Figure 48. XIC of the Oxy-catalysed turnover of kistamicin pentapeptide K3-7

Extracted ions from analysis in positive mode $[M+H]^+$ of linear pentapeptide K3-7 methylamide with a mass of 791.3 Da (black), monocyclic methylamide (MA) product with the mass of 789.3 Da (pink) and bicyclic methylamide product with the mass of 787.3 Da (blue) following methylamine cleavage. Enzyme components present in each reaction are indicated adjacent to the Y-axis in each trace within the figure. Source data are provided as a Source Data file.

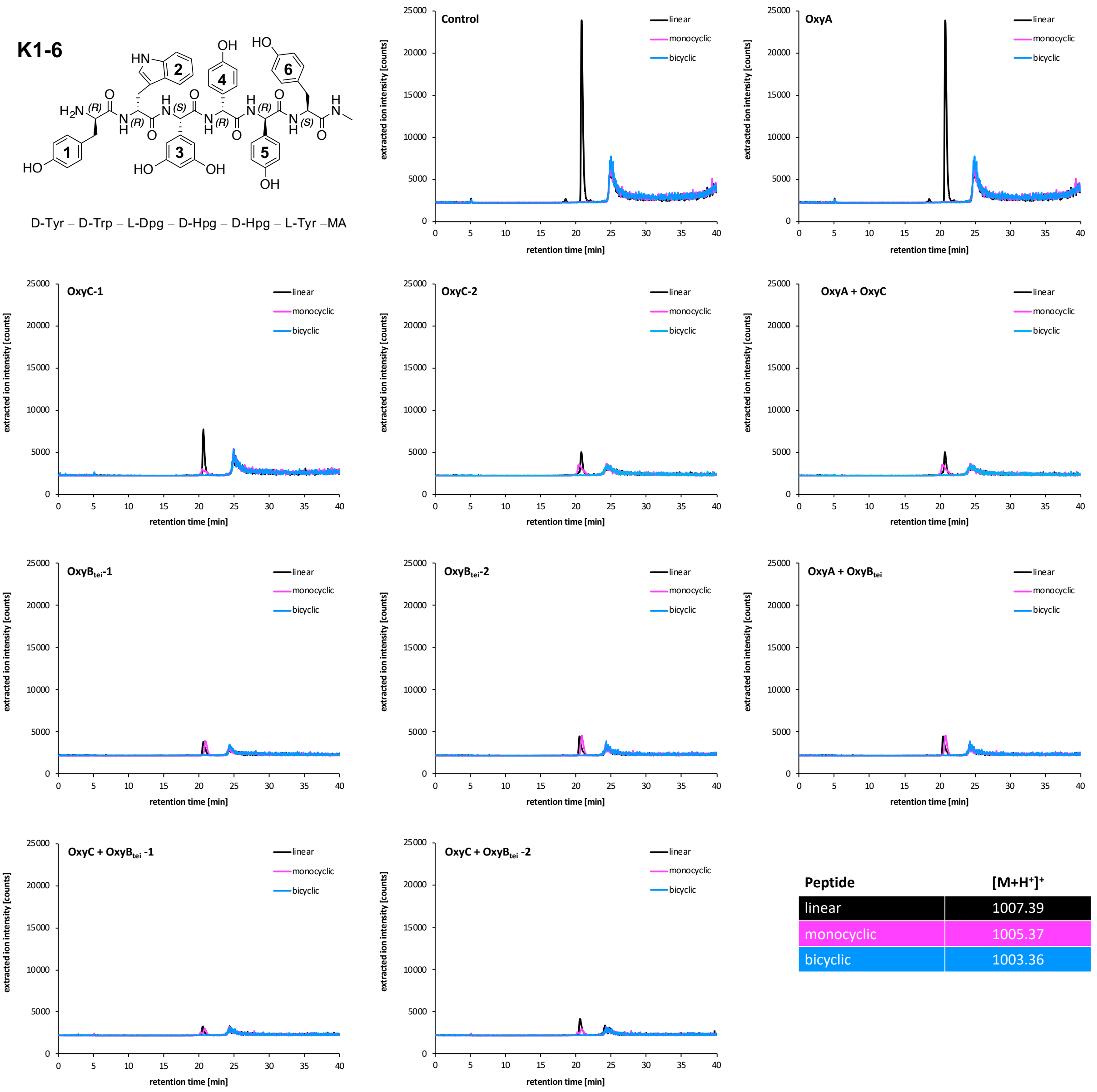

Peptide	$[M+H^+]^+$
linear	1007.39
monocyclic	1005.37
bicyclic	1003.36

Supplementary Figure 49. XIC of the Oxy-catalysed turnover of kistamicin hexapeptide K1-6

Extracted ions from analysis in positive mode $[M+H]^+$ of linear hexapeptide K1-6 methylamide with a mass of 1007.4 Da (blue), monocyclic methylamide (MA) product with the mass of 1005.4 Da (cyan) and bicyclic methylamide product with the mass of 1003.4 Da (green) following methylamine cleavage. Enzyme components present in each reaction are indicated adjacent to the Y-axis in each trace within the figure. Source data are provided as a Source Data file.

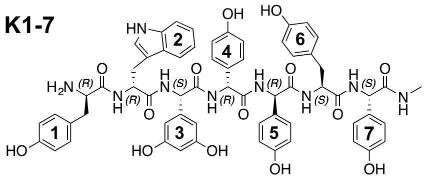

D-Tyr - D-Trp - L-Dpg - D-Hpg - D-Hpg - L-Tyr - L-Hpg - MA

Peptide	$[M+H^+]^+$
linear	1156.43
monocyclic	1154.42
bicyclic	1152.40
tricyclic	1150.39

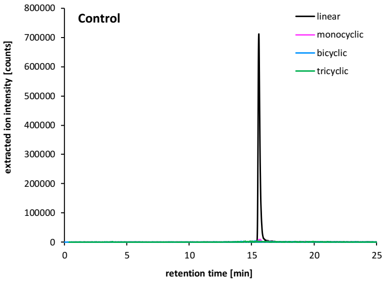

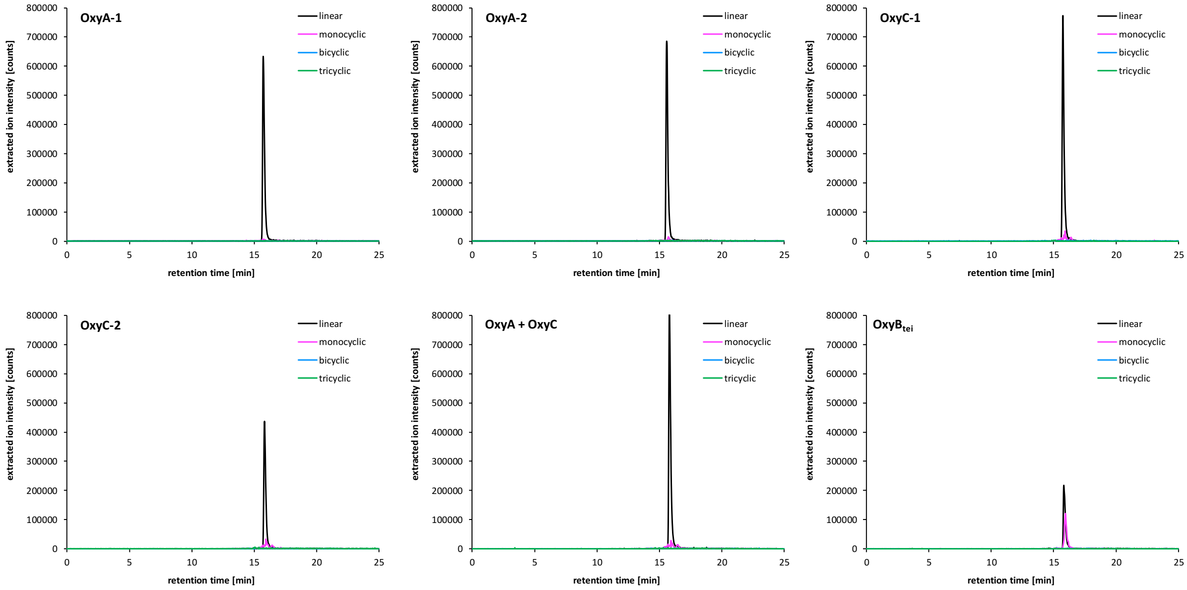

Supplementary Figure 50. XIC of the Oxy-catalysed turnover of kistamicin heptapeptide K1-7
Extracted ions from analysis in positive mode $[M+H]^+$ of the linear heptapeptide K1-7 methylamide (MA) with a mass of 1156.43 Da (black), monocyclic methylamide product with the mass of 1154.42 Da (pink) following methylamine cleavage, bicyclic product with the mass of 1152.40 Da (blue) and tricyclic product with the mass of 1150.39 Da (green). Enzyme components present in each reaction are indicated adjacent to the Y-axis in each trace within the figure. Source data are provided as a Source Data file.

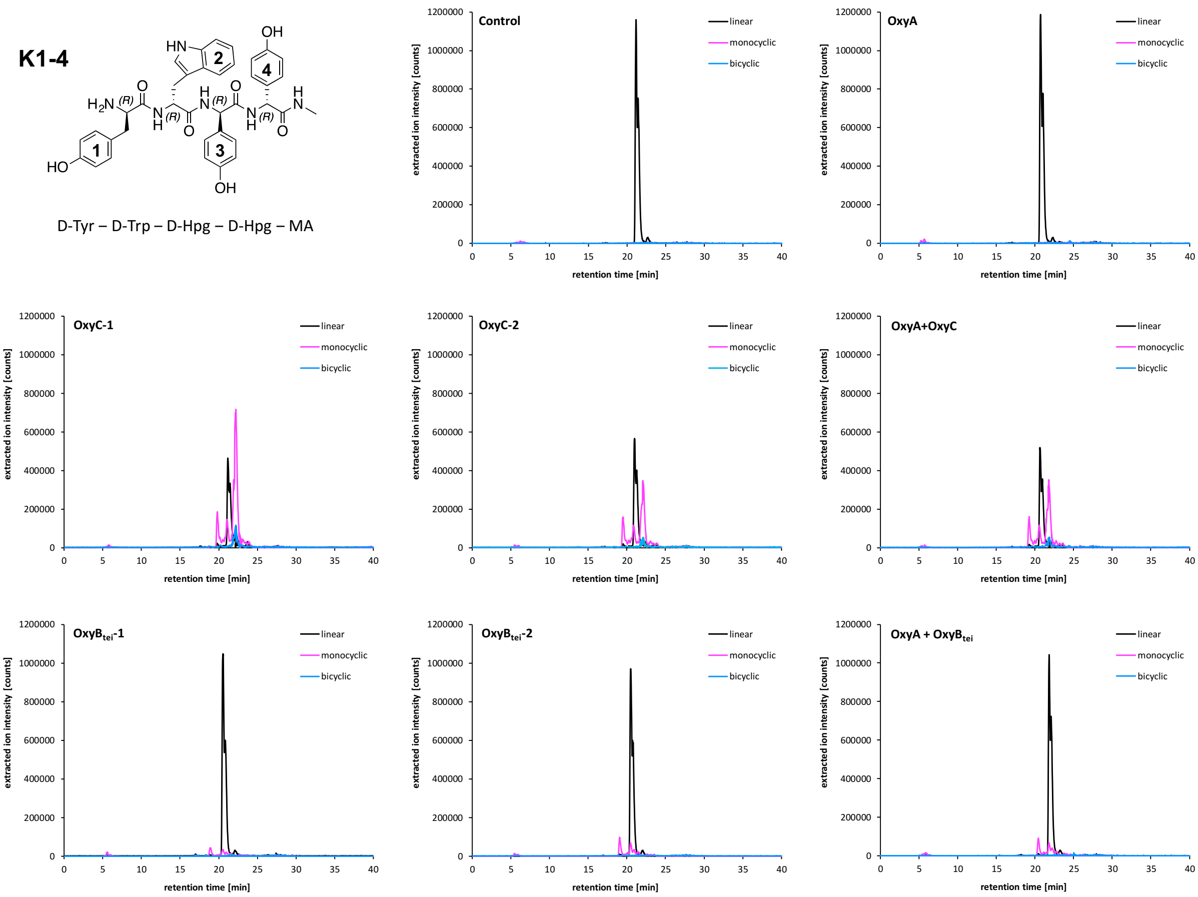

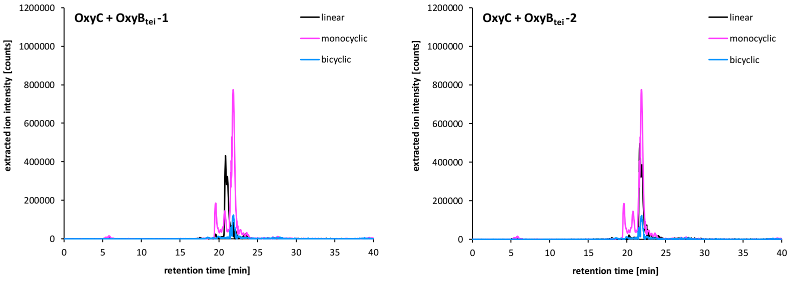

Peptide	$[M+H^+]^+$
linear	679.29
monocyclic	677.27
bicyclic	675.26

Supplementary Figure 51. XIC of the Oxy-catalysed turnover of kistamicin tetrapeptide K1-4

Extracted ions from analysis in positive mode $[M+H]^+$ of linear tetrapeptide K1-4 methylamide with a mass of 679.29 Da (black), monocyclic methylamide (MA) product with the mass of 677.27 (pink) and bicyclic methylamide product with the mass of 675.26 Da (blue) following methylamine cleavage. Enzyme components present in each reaction are indicated adjacent to the Y-axis in each trace within the figure. Source data are provided as a Source Data file.

K2-4D

(A)

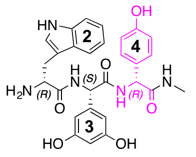

D-Trp – L-Dpg – D-Hpg – MA

Peptide	$[M+H^+]^+$
linear	532.21
monocyclic	530.20

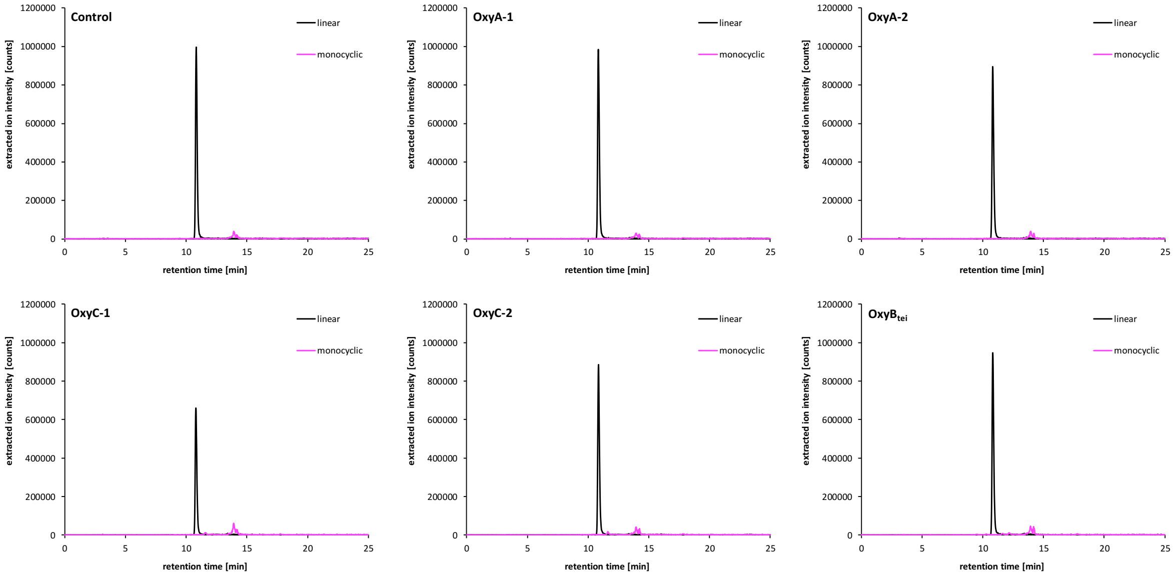

K2-4L

(B)

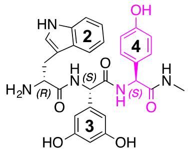

D-Trp – L-Dpg – L-Hpg – MA

Peptide	$[M+H^+]^+$
linear	532.21
monocyclic	530.20

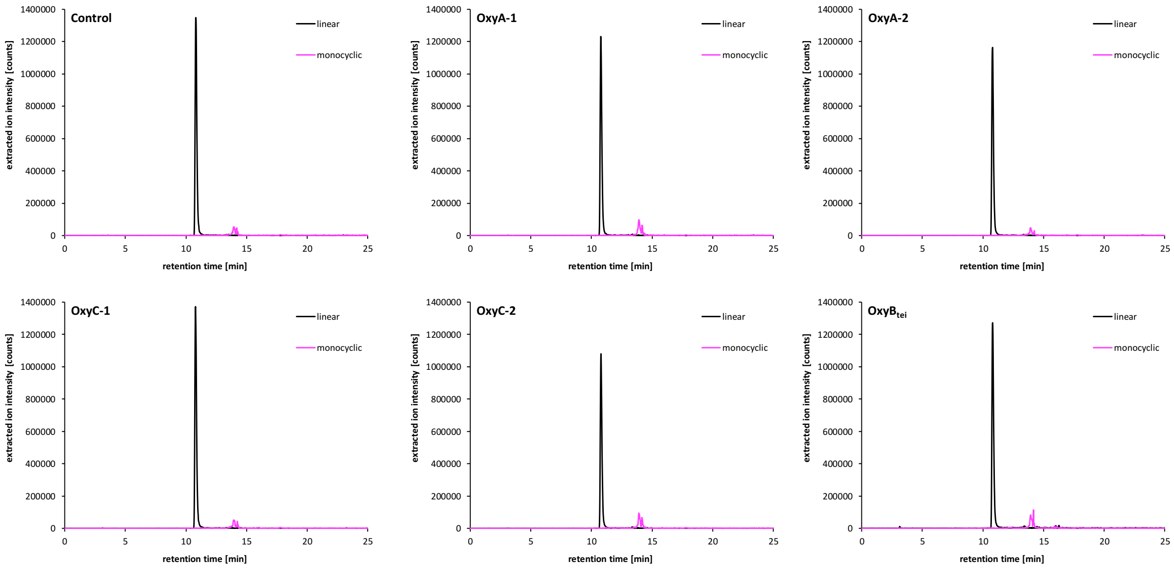

Supplementary Figure 52. XIC of the Oxy-catalysed turnover of kistamicin tripeptides K2-4
Extracted ions from analysis in positive mode $[M+H]^+$ of linear tripeptide methylamide with a mass of 532.21 Da (black) and monocyclic methylamide (MA) product with the mass of 530.2 Da (pink). (A) K2-4D, (B) K2-4L. Enzyme components present in each reaction are indicated adjacent to the Y-axis in each trace within the figure. Source data are provided as a Source Data file.

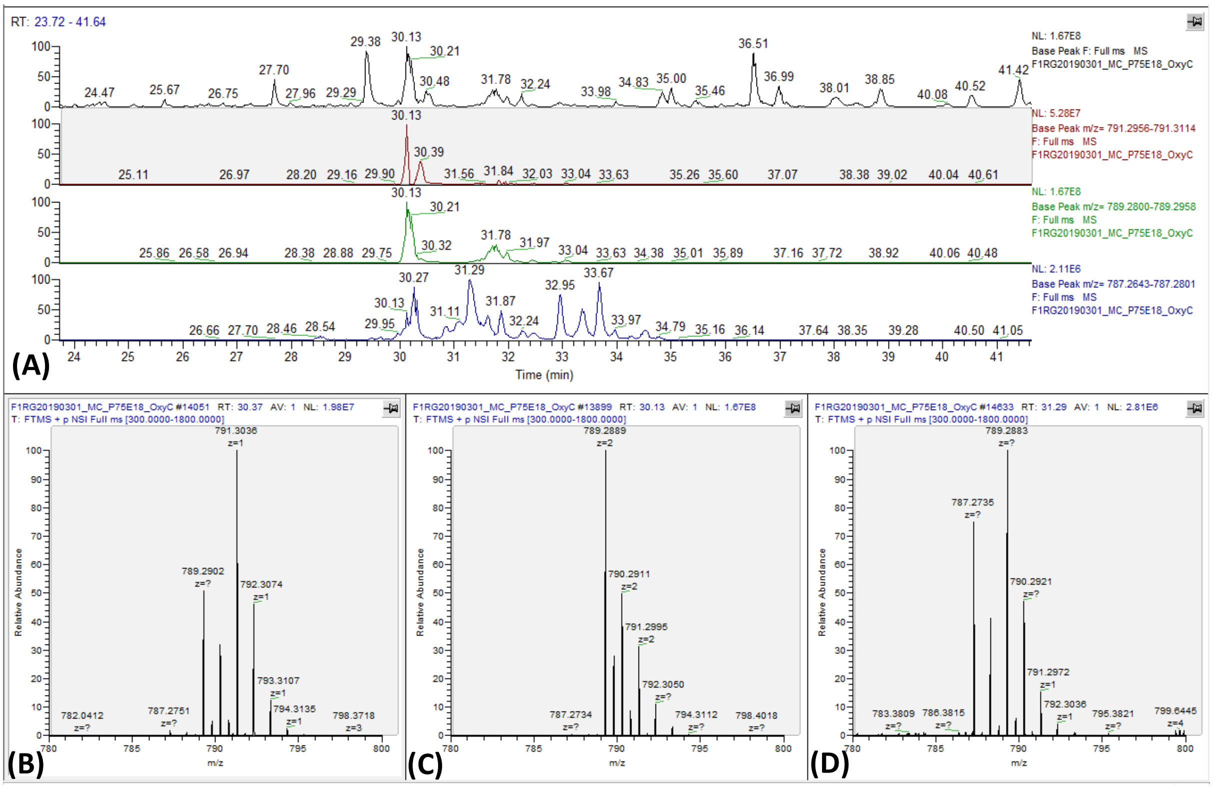

Supplementary Figure 53. HRMS analysis of OxyC turnover product K3-7

In the *in vitro* K3-7 assay with OxyC, there is a monocyclic pentapeptide and numerous bicyclic peptides present. (A) Base peak chromatogram of all ions (black), and extracted ion chromatograms of linear 791.3035 $[M+H]^+$ (red), monocyclic 789.2879 $[M+H]^+$ (green) and bicyclic 787.2722 $[M+H]^+$ (blue) at 10 ppm. HRMS highlighting the linear (B), monocyclic (C) and bicyclic (D) pentapeptide. The abundant monocyclic co-elutes with both other versions, and forms low level gas-phase dimers.

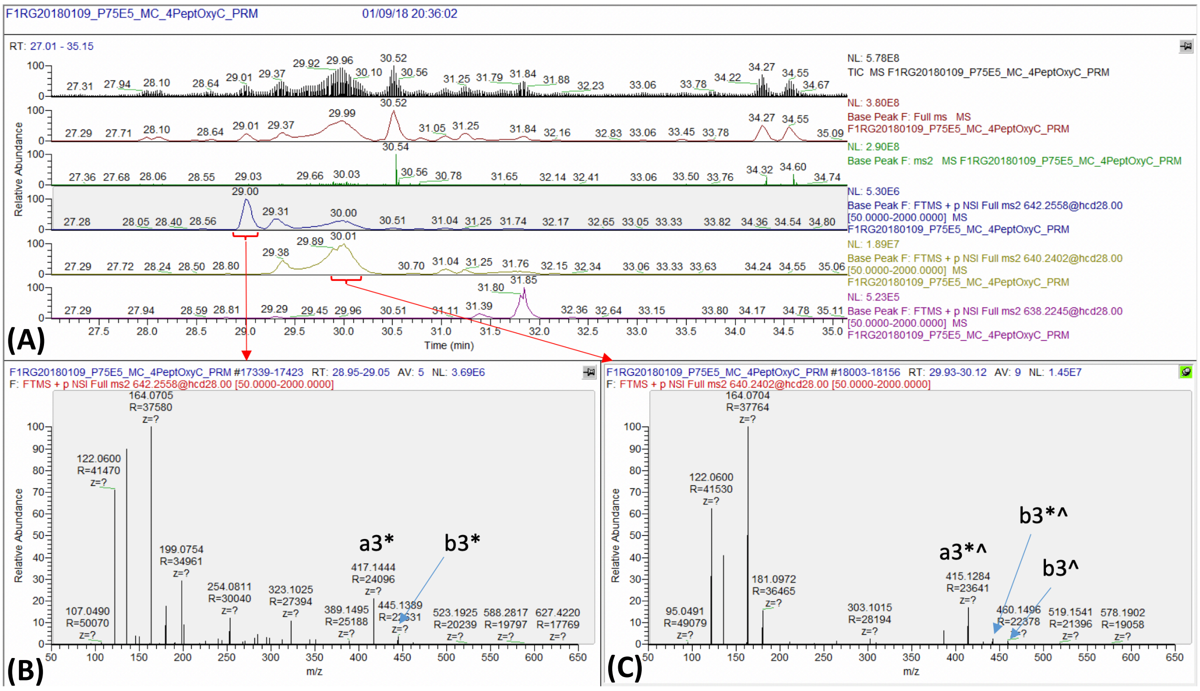

Supplementary Figure 54. Analysis of OxyC turnover product K4-7

In the *in vitro* K4-7 assay with OxyC, there are the linear tetrapeptide 642.2558 $[M+H]^+$ and one monocyclic tetrapeptide 640.2402 $[M+H]^+$. (A) HPLC chromatograms, from top to bottom: TIC, Base peak full scan, Base peak MS2 scans, XIC linear tetrapeptide 642.2558 $[M+H]^+$, XIC monocyclic tetrapeptide 640.2402 $[M+H]^+$, XIC bicyclic tetrapeptide 638.22 $[M+H]^+$. MSMS spectra of linear K4-7 (B) and monocyclic tetrapeptide (C). The monocyclic tetrapeptide appears to has a Hpg4-Tyr6 crosslink, as the Hpg7-MA is cleaved off. Fragmentation ions follow standard nomenclature for such experiments, see [3,4].

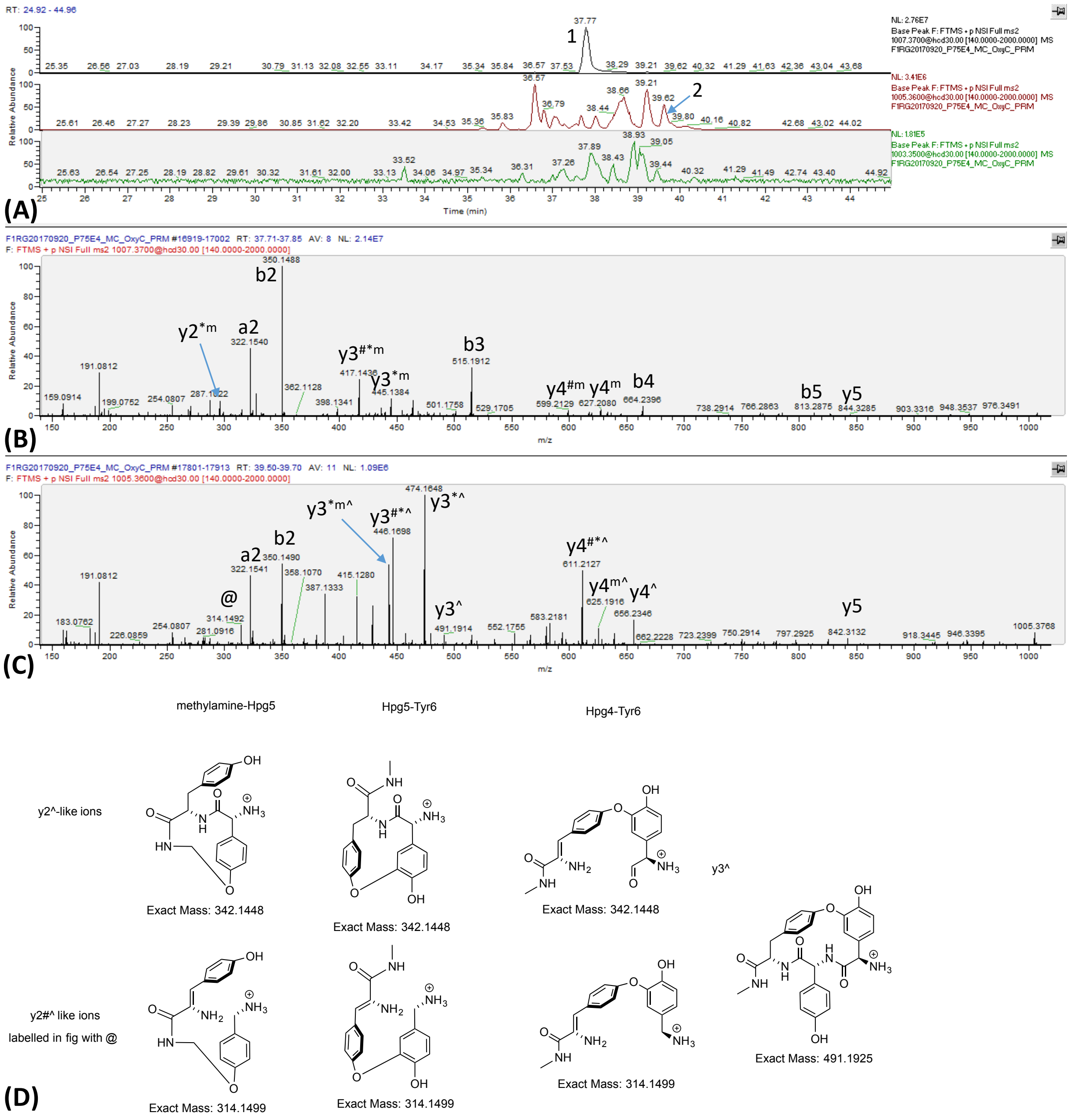

Supplementary Figure 55. Analysis of OxyC turnover product K1-6

In the *in vitro* K1-6 assay with OxyC, there is the linear hexapeptide 1007.3934 $[M+H]^+$, several monocyclic hexapeptides 1005.3777 $[M+H]^+$ and low levels of bicyclic hexapeptide 1003.3621 $[M+H]^+$. (A) Base peak chromatogram of linear (black), monocyclic (red) and bicyclic (green) hexapeptide targeted PRM, with selected peaks labelled. Representative MSMS spectra of labelled peaks of linear (B, peak 1 (37.7 min)) and monocyclic peptides (C, peak 2 (39.5 min)). The monocyclic spectrum shown supports a Hpg4-Tyr6 crosslink. The peak at 314.1492m/z, labelled with @, represents a loss of Hpg and CO from the y3^ ion, which due to the sequence is somewhat ambiguous; however, it is more likely to result from a Hpg4-Tyr6 crosslink through loss of the Hpg5 residue and CO, rather than a loss of CO from a y2^ (representing a Hpg5-Tyr6 crosslink) as there is no loss of methylamine. Possible fragments are shown in (D), indicating different fragments with similar masses that makes analysis difficult. Several other linkages were present in the other monocyclic and bicyclic peaks. Fragmentation ions follow standard nomenclature for such experiments, see [3,4].

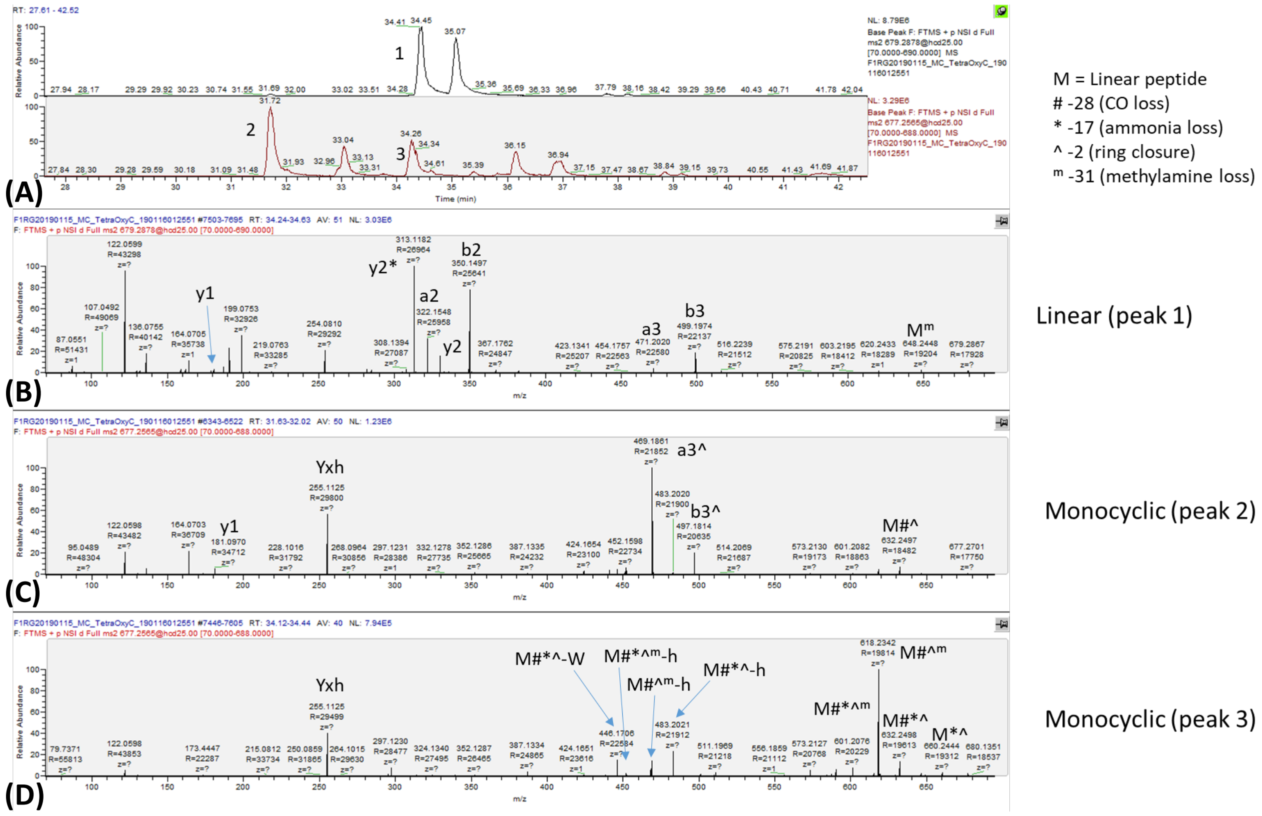

Supplementary Figure 56. Analysis of OxyC turnover product K1-4

In the *in vitro* K1-4 assay with OxyC, there are the linear tetrapeptide 679.2875 $[M+H]^+$ and two different kind of monocyclic tetrapeptides 677.2718 $[M+H]^+$. (A) Base peak chromatogram of linear (black) and monocyclic tetrapeptide (red). (B-D) MSMS of linear (B, peak 1 (34.2 min)) and monocyclic tetrapeptide (C, peak 2 (31.6 min), and D, peak 3 (34.3 min). Peak 2 appears to have a Tyr1-Hpg3 crosslink. Peak 3 represents a Tyr1-Hpg4 linkage, due to 255 ion abundance (linked Tyr and Hpg) and losses of Hpg and Trp from the parent with CO loss and virtual absence of both y1 and b3. Fragmentation ions follow standard nomenclature for such experiments, see [3,4].

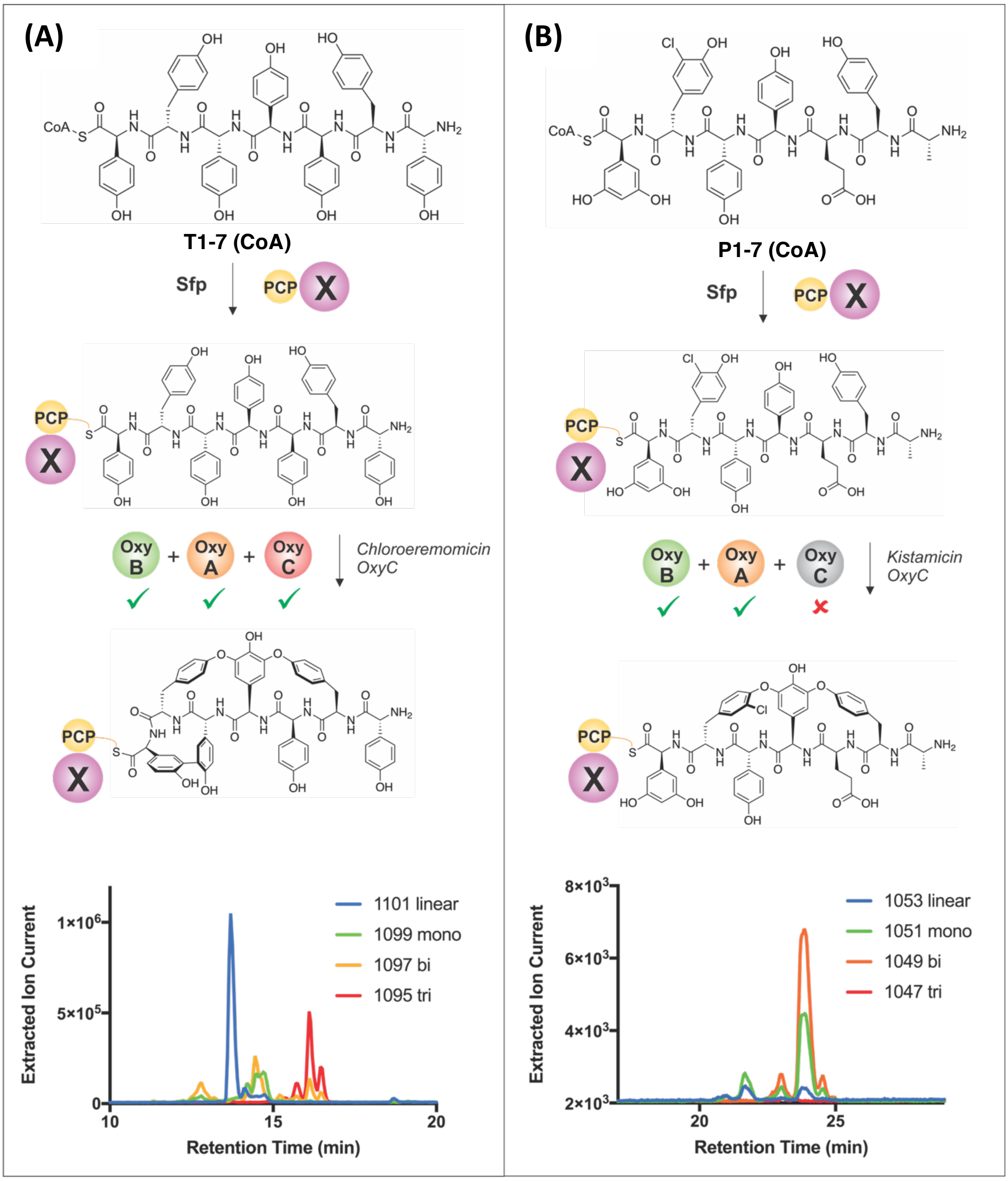

Supplementary Figure 57. Comparison of the activity of OxyC homologues

A turnover assay was performed with a GPA type I OxyC (from chloroeremomycin) and type V OxyC (from kistamicin) to check AB/ A-*O*-B ring formation in peptides with different C-terminal residues. Peptides tested include a teicoplanin related peptide T1-7 bearing a Hpg7 residue (A) and a pekiskomycin (Type I) peptide P1-7 bearing a Dpg-7 residue (B). These assays show that the chloroeremomycin OxyC homologue is active for AB ring formation in the peptide with a Hpg-7 residue (13-membered ring based on comparison to reported activity for balhimycin OxyC homologue),[5] whilst the kistamicin OxyC homologue is not active for A-*O*-B ring formation in a peptide with a Dpg-7 residue. Peptide preparation and cyclisation assays performed using reported protocols.[6-8] Source data are provided as a Source Data file.

Supplementary Tables

Supplementary Table 1. ^{1}H and ^{13}C NMR spectroscopic data and HMBC correlations for Kistamicin A (700 MHz, methanol-d_4) Numbering of Kistamicin A according Supplementary Figure 4, HMBC correlation Supplementary Figure 5 and NMR spectra Supplementary Figures 6-11.

Units	^{1}H	^{13}C	HMBC	Units	^{1}H	^{13}C	HMBC
A-1	-	135.9	-	D-1	-	131.1	-
A-2	7.73	134.7	A-6, A-4	D-2	7.26	121.3	D-5, D-6
A-3	7.57	124.4	A-1	D-3	6.96	118.1	D-1, D-4,
A-4	-	156.3	-	D-4	-	149.4	-
A-5	7.24	125.8	A-1, A-4	D-5	-	145.7	-
A-6	7.83	131.8	A-2, A-4	D-6	5.24	112.9	D-2, D-4,
A-1’	2.92, 3.61	41.7	A-2’, A-1,	D-1’	5.43	58.7	D-1, D-6
A-2’	4.33	59.3	-	D-2’	-	170.6	-
A-3’	-	170.6	-	D-NH*	8.55	-	-
A-NH	8.17	-	E-1’	E-1	-	136.8	-
B-1	-	130.4	-	E-2	7.96	135.1	E-3, E-4,
B-2	5.55	132.5	B-3, B-4,	E-3	-	130.6	-
B-3	-	136.4	-	E-4	-	154.2	-
B-4	-	140.3	-	E-5	6.84	128.1	E-1, E-3,
B-5	-	150.7	-	E-6	7.38	130.4	E-2, E-4
B-6	5.70	109.8	B-2, B-4,	E-1’	4.99	55.5	E-1, E-6
B-1’	6.40	56.5	B-3	E-2’	-	171.3	-
B-2’	-	171.3	-	E-NH	9.53	-	-
B-NH*	8.37	-	-	F-1	-	141.8	-
C-2	6.99	125.8	C-3, C-4,	F-2,6	6.15	105.2	F-3,5, F-4,
C-3	-	111.8	-	F-3,5	-	159.7	-
C-4	-	127.2	-	F-4	5.78	102.4	F-2,6
C-5	7.99	121.9	C-9	F-1’	4.94	58.0	F-1, F-2,6
C-6	6.84	123.4	C-4, C-7,	F-2’	-	172.1	-
C-7	-	133.1	-	F-NH	8.75	8.75	-
C-8	7.32	116.5	C-4, C-6,	G-1	-	126.9	-
C-9	-	139.1	-	G-2,6	6.70	131.8	G-4, G-1’
C-1’	3.01, 3.59	30.5	C-3, C-4	G-3,5	6.31	116.8	G-1, G-4
C-2’	6.29	54.9	-	G-4	-	157.8	-
C-3’	-	170.9	-	G-1’	2.90, 3.45	38.4	G-2,6, G-
C-NH*	9.29	-	-	G-2’	4.06	56.9	-
C-1-NH*	10.28	-	-	G-3’	-	175.6	-

* observed in methanol-d_3 (where all the amide protons were visible)

Supplementary Table 2. Bacterial strains and plasmids used in this study

Strains	Characteristics	Reference
E. coli NovaBlue	*recA*1, *endA*1, *gyrA*96, *thi*-1, *hsdR*17 (rK12$^-$,mK12$^+$) *supE*44, *relA*1, *lac* [F', *pro*AB, *lacI*^{q}, *lacZ*ΔM15, Tn*10*] (Tet^R)	Novagen
E. coli ET12567	DNA methylation deficient donor strain for conjugation: F2*dam*13::Tn*9*, *dcm*-6, *hsdM, hsdR, recF143, zjj-202::*Tn*10, galK2, galT22 ara-14, lacY1,xyl-5, leuB6, thi-1, tonA31, rpsL136, hisG4, tsx-78, mtl-1, glnV44*	MacNeil, *et al.*, 1992. [9]
E. coli ArcticExpress	*E. coli* B F$^-$ *ompT hsdS*(r_B^- m_B^-) *dcm*$^+$ *Tet*^{r}*, gal* λ(DE3) *endA* Hte [*cpn10 cpn60* Genta^r]	Agilent Technologies
**Plasmids**		
pJet1.2/blunt	rep(pMB1), *bla*(Amp^R), *eco47IR*, P_{lacUV5}, T7 RNA polymerase promoter. Part of the CloneJET™ PCR Cloning Kit used for high efficiency cloning of PCR products	Thermo Fisher Scientific Inc.
pRM4.2	pSET152*erm*Ep*derivate (ΦC31 integration vector with *ermE** promoter, Apra^R) with artificial RBS, used for construction of pRM4_kisN and pRM4_KisO	Menges, *et al.*, 2007.[10]
pUB307	self-transmissible plasmid that mobilizes other plasmids in trans for DNA transfer into hosts: RP4, kan^R	Bennett, *et al.*, 1977.[11]
pGUSA21	Promoter probe vector, pSETGUS with deleted KpnI fragment containing TipA promoter. gusA, Δint, ΔattB, MCS from pUC21. Used for gene inactivation	Myronovskyi *et al.*, 2011.[12]
pGUSA21_KisN-up_down	pGUSA21 deletion plasmid of kisN containing 1.5kb flanking regions each up-and downstream of the *kisN* gene	This study
pGUSA21_KisO-up_down	pGUSA21 deletion plasmid of kisO containing 1.5kb flanking regions each up-and downstream of the *kisO* gene	This study
pRM4_kisN	pRM4 with *kisN* cloned in *Nde*I + *Hin*dIII site	This study
pRM4_KisO	pRM4 with *kisO* cloned in *Hin*dIII + *Xba*I site	This study

Supplementary Table 3. Primers used in this study

Primer Name	Sequence	Amplicon length bp	PCR Programm
KisN-down Fw	CGCCTCTAGAGTCCCGTCGATCAC	1520	1. 98°C, 0:30 min<br>2. 98°C, 0:10 min<br>3. 70°C, 0:30 min<br>4. 72°C, 1:30 min<br>5. 72°C, 2:00 min<br>(Steps 2-4 × 32)
KisN-down Rv	GCGGCATATGGGGTGCCATGCTT		
KisN-up Fw	CGGACATATGACGGCGCCGTCCGC	1520	
KisN-up Rv	CGCCAAGCTTCGCGGCCAGGGACT		
KisO-down Fw	GCACTCTAGAGGCAGCACTCCCTGTG	1519	
KisO-down Rv	GGACCATATGCACAGCTCCTTCACAGAG		
KisO-up Fw	GCTGCATATGGCGCGGCGAGCCGGG	1520	
KisO-up Rv	CTGCAAGCTTGTGCGGGGCGCTCGG		
pGus-Fw	CGCCGTTGGATACACCAAGG	3271	1. 98°C, 0:30 min<br>2. 98°C, 0:10 min<br>3. 60°C, 0:30 min<br>4. 72°C, 3:00 min<br>5. 72°C, 2:00 min<br>(Steps 2-4 × 32)
pGus-Rw	GTGGACCGGGATGAACTTCG		
pRM4SeqFw	CACACGTGGCACCGCGATGCTGTTG	1336	1. 98°C, 0:30 min<br>2. 98°C, 0:10 min<br>3. 64°C, 0:30 min<br>4. 72°C, 1:30 min<br>5. 72°C, 2:00 min<br>(Steps 2-4 × 32)
pRM4SeqRv	GTTGTAAAACGACGGCCAGTGCCAAG		
kisN-NdeI-Fw	GCTGCATATGGTCGCGCCGGAGC	1172	1. 98°C, 0:30 min<br>2. 98°C, 0:10 min<br>3. 63°C, 0:30 min<br>4. 72°C, 1:30 min<br>5. 72°C, 2:00 min<br>(Steps 2-4 × 32)
kisN-HindIII-Rv	CTCGAAGCTTTCACCAGGTCAACGGCAG		
kisO-HindIII-Fw	CTGCAAGCTTATGACGGATGTGACGCCAG	1301	
kisO-XbaI-Rv	GCGGTCTAGATCATCGGGTCGCGGC		
Delta kisN Fw	GCGCGCACCTGTTCCCGCTCA	2500 (wt)	1. 98°C, 0:30 min<br>2. 98°C, 0:10 min<br>3. 63°C, 0:30 min<br>4. 72°C, 1:30 min<br>5. 72°C, 2:00 min<br>(Steps 2-4 × 32)
Delta kisN Rv	CGGACGCTGTAGGCGGGCG		
Delta kisN Fw	GCGCGCACCTGTTCCCGCTCA	4708 (wt)<br>3553 (Δ*kisN*)	1. 98°C, 0:30 min<br>2. 98°C, 0:10 min<br>3. 78°C, 0:30 min<br>4. 72°C, 3:30 min<br>5. 72°C, 2:00 min<br>(Steps 2-4 × 32)
kisO-XbaI-Rv	GCGGTCTAGATCATCGGGTCGCGGC		
Delta KisO f	CATCTGGCTGGCCACCGGGTAC	2800 (wt)	
Delta KisO r	CGCCCTGCGATTCCGCGGC		
X-for	ATTATAccatggcaCGTCCAGTCCTTGGTCCG	1519	
T7-rev	GCTAGTTATTGCTCAGCGG		

Supplementary Table 4: Media used in this study

Name	Mass (g/l)	Further information
**SFM-agar**		
mannitol	20	
soy meal full fat	20	
agar	16	
**LB Broth**		
tryptone	10	
yeast extract	5	
NaCl	5	
**R5 medium**		pH 7.2; for plates 16g/L agar
Sucrose	103	
K_2SO_4	0.25	
$MgCl_2$ ($6xH_2O$)	10.12	
Glucose	10	
Difco Casaminoacids	0.1	
Trace elements	2ml	
Difco Yeast extract	5	
TES Puffer	5.73	
	0.893 L ion free H_2O	autoclave separately
**Supplementation R5**		autoclave separately
$CaCl_2$	80 mL	3.68%
KH_2PO_4	10 mL	0.54%
L- Proline	15 mL	20%
**Trace elements**	1 L	autoclave separately
$FeCl_3$ x $6H_2O$	200 mg	
$Na_2B_4O_7$ x $10H_2O$	10 mg	
$(NH_4)6Mo_7O_{24}$ x $4H_2O$	10 mg	
$CuCl_2$ $x2H_2O$	10 mg	
$MnCl_2$ $x4H_2O$	10 mg	
$ZnCl_2$	40 mg	
**ZYM 5052 Media**		pH 7.4
Tryptone	10 g	
Yeast extract	5 g	
Na_2HPO_4	25 mM	
KH_2PO_4	25 mM	
NH_4Cl	50 mM	
$NaSO_4$	5 mM	
$MgSO_4$	2 mM	
Glycerol	5 g	
D-Glucose	0.5 g	
Lactose	2 g	

Supplementary Table 5. Crystallography data for the $OxyA_{kis}$/X_{kis} complex

**Data Collection**	
Beamline	MX1, AS
Wavelength (Å)	0.9537
Space group	C2
Cell parameters<br>*a, b, c* (Å)<br>*x, y, z* (°)	 118.8, 87.3, 96.0;<br>90.00, 95.77, 90.00.
**Data statistics**	
Resolution (Å)	50.0 – 2.65
Completeness (%) [(1)]	99.7 (98.9)
Multiplicity[1]	3.8 (3.8)
(I) / σ(I) [(1)]	21.3 (2.1)
R_{merge} [(1)]	0.04 (0.50)
R_{pim} [(1)]	0.026 (0.46)
CC1/2 [(1)]	0.99 (0.83)
**Refinement**	
R / R_{free} [(2)]	0.212 / 0.264
Bond length rmsd (Å)	0.004
Bond angle rmsd (°)	0.634
Favored (%) [(3)]	94.7
Disallowed (%) [(3)]	0.7
MOLPROBITY score	2.27 (92nd percentile)
**PDB ID**	6M7L

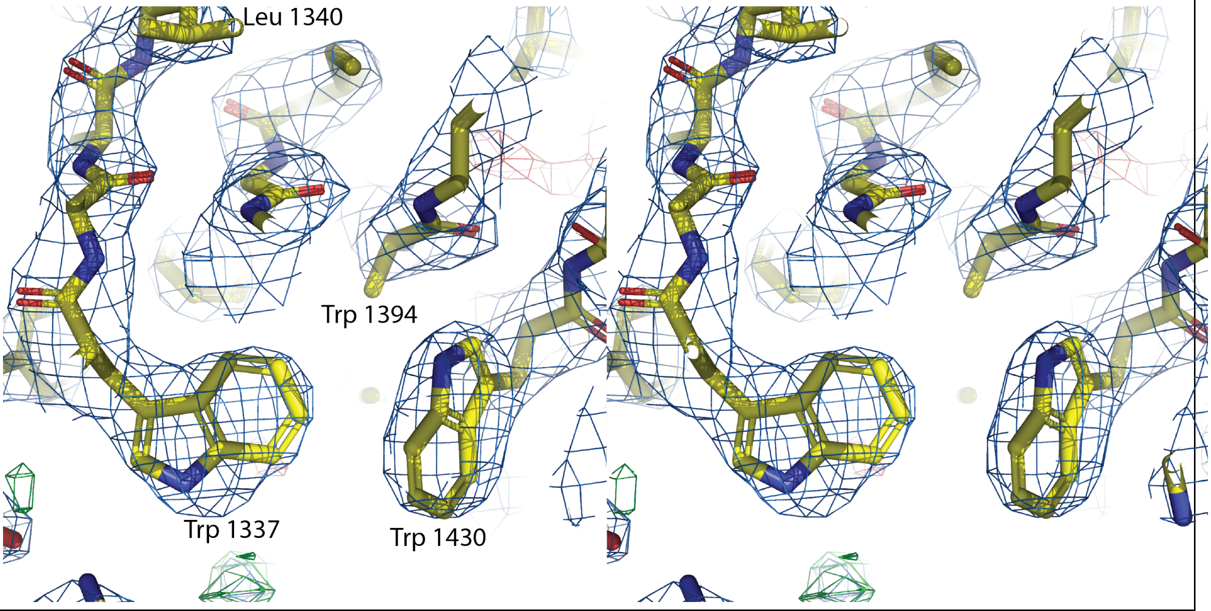

Stereo image of a portion of the electron density map for the X-domain (maps: 2Fo-Fc (blue, sigma level 1.5), positive Fo-Fc (green, sigma level 3.0) and negative Fo-Fc (red, sigma level 3.0)).

[(1)] Values in parentheses refer to the highest recorded resolution shell.
[(2)] 5% of reflections were randomly selected before refinement.
[(3)] Percentage of residues in the Ramachandran plot

Supplementary Table 6. Similar proteins to OxyA from kistamicin biosynthesis

Top ranking and carrier-protein interacting P450 structures with high levels of homology to the $OxyA_{kis}$ in complex with the X-domain as identified by Dali.[13]

PDB code	Chain	Z-score	RMSD Cα [Å]	% Identity	Description (donor organism)	Ref
5HH3	C	44.2	1.5	48	$OxyA_{tei}$ (*Actinoplanes teichomyceticus*)	14
3O1A	A	43.5	2.2	50	$OxyE_{tei}$ (CYP165D3, *Actinoplanes teichomyceticus*)	15
1UED	A	43.2	2.1	44	$OxyC_{van}$ (*Amycolatopsis orientalis*)	16
5EX8	A	43.0	2.1	39	StaF (*Streptomyces toyocaensis*)	17
1LGF	A	42.9	2.0	43	$OxyB_{van}$ (*Amycolatopsis orientalis*)	18
5EX6	C	42.7	1.9	44	StaH (*Streptomyces toyocaensis*)	19
6FSH	D	41.4	2.0	44	Hybrid $OxyB_{tei}$ BC/FG_{van} (*Actinoplanes teichomyceticus*)	20
4UBS	A	41.2	2.6	37	CYP105D7 (*Streptomyces avermitilis*)	21
5Y1I	B	41.1	2.2	35	GfsF (*Streptomyces graminofaciens*)	22
2ZBZ	A	40.9	2.8	33	CYP105A1 (*Streptomyces griseolus*)	23
4TVF	A	40.6	2.1	45	$OxyB_{tei}$ (*Actinoplanes teichomyceticus*)	24
3BUJ	A	39.4	2.2	32	Cal02 (*Micromonospora echinospora*)	25
3EJB	H	38.5	2.5	34	$P450_{BioI}$ (CYP107H1, *Bacillus subtilis*)	26
4L0E	A	36.4	2.5	24	$P450_{sky}$ (CYP163B3, *Streptomyces* sp. ACTA 2897)	27,28
3MGX	B	33.7	3.1	28	OxyD (*Amycolatopsis balhimycina*)	29

Supplementary Table 7. Similar proteins to the X domain from kistamicin biosynthesis

Top ranking structures with high levels of homology to the X_{kis}-domain in complex with $OxyA_{kis}$ as identified by Dali.[13]

PDB code	Chain	Z-score	RMSD Cα [Å]	% Identity	Description (donor organism)	Ref
4TX2	B	49.1	2.1	55	X-domain from teicoplanin Tcp12 synthetase (*Actinoplanes teichomyceticus*)	30
4TX3	B	48.9	1.6	55	X-domain from teicoplanin Tcp12 synthetase (*Actinoplanes teichomyceticus*)	30
2JGP	A	38.4	3.2	24	PCP and C-domain from tyrocidine synthetase (*Brevibacillus brevis*)	31
5U89	A	35.5	2.8	38	A, PCP and C domains from baccilibactin synthetase (*Geobacillus* sp. Y4.1MC1)	32
4ZXI	A	34.5	3.2	23	C-A-PCP-TE termination module from *holo*-AB3403 synthetase (*Acinetobacter baumannii*)	33
5DU9	A	33.9	4.9	19	C-domain from calcium-dependent antibiotic synthetase bound to acceptor mimic (*Streptomyces coelicolor A3(2))*	34
4JN3	B	32.7	4.9	18	C-domain from calcium-dependent antibiotic synthetase (*Streptomyces coelicolor A3(2))*	35
5T3D	A	31.7	3.5	19	C-A-PCP-TT termination module from enterobactin synthetase (*Escherichia coli*)	33
2VSQ	A	31.0	4.7	21	C-A-PCP-TE termination module from surfactin synthetase (*Bacillus subtilis*)	36

Supplementary Table 8. Comparison of the protein-protein interface within the two Oxy/X-domain complexes identified to date as performed by PISA.[37]

Complex	Residues (X-domain)	Residues (Oxy)	Interface ($Å^2$)	ΔG (kcal/mol)	ΔG P-value [a]	H-bonds	Salt bridges
OxyB-X_{tei}	24	23	725	2.2	0.761	17	16
OxyA-X_{kis}	19	33	976	2.6	0.740	17	20

[a] indication of hydrophobic nature of the interface, > 0.5 indicates the interface is less hydrophobic than usual for a protein-protein interface

Supplementary Table 9. Theoretical molecular weight of synthesised kistamicin peptides
Masses of starting material peptide-CoAs and their subsequent modifications during turnovers.
MA: peptide generated from methylamine cleavage to generate methylamide peptide

Peptide	-CoA	MA linear	MA 1 ring	MA 2 rings	MA 3 rings
**K1-7**	1891.51	1155.43	1153.42	1151.40	1149.39
**K4-7**	1377.32	641.25	639.23	637.22	-
**K2-4**	1267.28	531.20	529.20	-	-
**K3-6**	1377.32	641.25	639.23	637.22	-
**K2-7**	1526.36	790.29	788.28	786.27	-
**K1-6**	1742.46	1006.39	1004.37	1002.36	1000.34
**K1-4**	1414.35	678.28	676.26	674.25	-
**P1-7**	1790.42	1054.35	1052.33	1050.32	1048.30
**T1-7**	1838.48	1102.41	1100.39	1098.38	1096.36

Supplementary Discussion

In terms of the peptide-producing NRPS, these differences are found in the 1st, 3rd and 4th modules, whilst the incorporation of Tyr-1, Trp-2 and Hpg-7 lead to a divergence in peptide structure (**Figure 1, Supplementary Figure 1**). In module 1, the lack of activation domain here implies that the tyrosine residue activated and loaded onto this position must be derived from an additional source. Previous work has shown that carrier protein (CP) domains can be aminoacylated by tRNA synthetases,[38] and that A-domains can be utilised to load tRNAs,[39] indicating a degree of crossover between these systems: this is one possible mechanism to account for the lack of the A_{core}-domain 1 in kistamicin biosynthesis. Furthermore, the D-configuration of this amino acid implies that the epimerisation of this residue would be occurring during initial peptide bond formation in the neighbouring condensation (C) domain, which is in agreement with early work on the chloroeremomycin (Type I) system.[40] Further important differences found in the kistamicin NRPS when compared to Type I-IV GPAs are the placement of E-domains within modules 3 and 4: specifically, the presence of an E-domain in module 3 and a lack of such a domain in module 4. Whilst a module 3 E-domain has previously been reported in the NRPS producing A47934,[41] this domain was shown to have been inactivated in this system, leading to the structure of A47934 matching other GPAs in possessing an (L)-configured residue at position 3 of the peptide. In the case of kistamicin, the presence of this E-domain agrees with the structural analysis performed for the initial identification of kistamicin that demonstrated a (D)-configured 3,5-dihydroxyphenylglycine (Dpg) residue at this position.[42] The results of $OxyC_{kis}$ reconstitution shows that peptides with residue 3 in a (D)-configuration are preferred over those with this residue in the (L)-configuration, which is consistent with the activity of this E-domain. The stereochemistry of the central Hpg residue installed by module 4 of the GPA NRPS is a key feature in all GPA structures, as this forms the central connection between both the C-*O*-D ring and D-*O*-E/ DE ring of the GPA. All GPA structures to date have shown this central residue to be (D) configured.[43,44] However, the kistamicin cluster does not possess an E-domain in this module (**Figure 1**). Given the central nature of this residue, alteration of the stereochemistry of this position to the (L)-form would appear highly unlikely, with $OxyC_{kis}$ reconstitution again showing the importance of a (D)-configuration of Hpg-4. This implies a dual C/E function for one of the neighbouring C-domains that are involved with peptide bond formation at this residue, although the identity of this potential dual C/E-domain is not clear from phylogenetic analyses (**Supplementary Figure 3**). When comparing kistamicin with the only other characterised Type-V GPA complestatin, the relative similarity of these molecules is not repeated in their NRPS architectures, as modules 1, 3 and 4 in the complestatin NRPS conform to that of a standard GPA.[45] Differences found within the complestatin NRPS – such as the extended linker between the X- and thioester (TE) domains in the final module, unique methyl transferase domain and

the silent additional E-domain within the 6th module – are not repeated within the kistamicin cluster, indicating significant diversity within the biosynthetic machineries of these two Type-V GPAs (**Figure 1**).[45]

Supplementary Methods

OxyE complementation in *A. parvosata* WT

Due to the results from the *A. parvosata* Δ*oxyA* (Δ*kisN*) strain showing that $OxyC_{kis}$ could install an addition crosslink between the Tyr-1 and Trp-2/ Dpg-3 residues of the C-*O*-D crosslinked peptide, we also tested whether the addition of a Type IV OxyE enzyme to the kistamicin WT producer would be able to reproduce the insertion of an extra ring at the N-terminus of kistamicin. To this end, we ascertained that the heterologous expression of *oxyE* (*staG*) gene[46] from the A47934 Type IV GPA into the *A. parvosata* WT kistamicin producer does not support insertion of an F-*O*-G ring between Tyr-1 and Dpg-3 in kistamicin. This lack of activity could stem from a number of reasons, concerning either the timing of OxyA ring formation restricting the activity of OxyE[47] or changes in the peptide structure or the stereochemistry of the Dpg-3 residue.

Molecular Modelling of the rings in kistamicin

Molecular modelling calculations were undertaken to compare the stability of native kistamicin (containing a 15-membered A-*O*-B ring) relative to kistamicin analogues containing 13- or 12-membered AB rings. The peptides were modelled as structures **1**–**3**, where the *N*-terminal tyrosine residue was replaced by an acetyl group (**Figure 3**). First, the conformations of each peptide were explored using the Macrocycle Conformational Sampling algorithm of MacroModel 11.7.[48,49] The conformer sampling was conducted with the OPLS_2005 forcefield,[50,51] using the GB/SA model[52] to simulate solvation in water. The default settings of the Macrocycle Conformational Sampling protocol in MacroModel were used, in which 5000 cycles of large-scale low mode searches were performed on a set of seed structures obtained from 5000 cycles of MD simulated annealing, with eigenvectors determined for each new global minimum, and included the "enhanced" option for torsional sampling (which samples certain C–N and C–O single bonds with higher rotational barriers). For each molecule, the three most stable conformers identified by this protocol were then submitted to single-point energy calculation with density functional theory (DFT), performed with M06-2X/6-31G(d)[53] in Gaussian 09.[54] The conformer having the lowest DFT energy was used for analysis. This series of calculations provided the relative potential energies reported in **Figure 3**, which predict that the native kistamicin structure, containing the 15-membered A-*O*-B ring (**1**), is about 10 kcal/mol less stable than the analogues containing the 13- (**2**) and 12-membered (**3**) AB rings. In a similar series of calculations where the conformer sampling was performed with the OPLS3 forcefield rather than with OPLS_2005, the order of stabilities of **1**–**3** was the same, the spread between the most stable and least stable isomers was smaller (5 kcal/mol).

Each molecule was then truncated to give just the A-*O*-B or AB ring as shown in **Supplementary Figure 27**. Free valences were capped with hydrogen atoms. The 15-, 13-, and 12-membered macromonocycles **4**–**6** were subjected to full geometry optimization with M06-2X/6-31G(d). This series of calculations showed that the 15-membered A-*O*-B ring substructure of kistamicin (**4**) was again about 10 kcal/mol less stable than the 12- or 13-membered AB rings of the analogues (**5**, **6**). This suggests that the origins of the difference in stability between native kistamicin and the smaller-ring AB analogues are localized largely within the A-*O*-B/AB ring.

A similar analysis was performed for the kistamicin C-*O*-D ring (**Supplementary Figure 28**). The native 16-membered C-*O*-D ring **7** was compared with the 14-membered CD ring analogue **8**. In this case, the C-*O*-D ring was 3–4 kcal/mol less stable than the CD ring. The 14-membered biaryl structure in **8** is analogous to that found in arylomycins.

We also explored ring size effects in pekiskomycin, a GPA which natively contains an AB ring (**Supplementary Figure 29**). The native structure **9**, containing a 12-membered AB ring, was compared with a ring-expanded analogue **10** containing a 14-membered A-*O*-B ring. In this case the native AB-containing structure was 2.7 kcal/mol more stable than the larger-ring A-*O*-B analogue.

Each of these sets of calculations reveals a common trend, namely that larger-ring diaryl ethers are less stable than the isomeric smaller-ring biaryl alcohols. This trend can, in part, be attributed to the intrinsic stabilities of diaryl ethers relative to biaryl alcohols. For example, calculations on diphenyl ether **11** and 2-hydroxybiphenyl **12** (**Supplementary Figure 30**) predict that ether **11** is about 7 kcal/mol less stable than biaryl alcohol **12**, the same trend observed in the GPAs above.

Ring strain also contributes to the relative stabilities of diaryl ether versus biaryl alcohol-containing macrocyclic rings in GPAs. In kistamicin, the B ring is a locus of deformation: as shown in **Supplementary Figure 31**, the aryl ring is puckered and the ether oxygen and α-carbon are bent significantly out of plane.

The Cartesian coordinates for **1**–**12** are provided in the raw data folder. For species **1**, **2**, **3**, **9** and **10**, where a DFT single-point energy calculation was performed using the OPLS_2005 optimized geometry, the M06-2X/6-31G(d) single-point electronic potential energy (E) is reported. For species **4**, **5**, **6**, **7**, **8**, **11** and **12**, which were fully optimized with DFT, both the M06-2X/6-31G(d) electronic potential energy (E) and Gibbs free energy (G, 298.15 K, 1 atm) are reported.

Supplementary References

1 Edgar, R. C. MUSCLE: multiple sequence alignment with high accuracy and high throughput. *Nucleic Acids Res* **32**, 1792-1797, (2004).

2 Stamatakis, A. RAxML version 8: a tool for phylogenetic analysis and post-analysis of large phylogenies. *Bioinformatics* **30**, 1312-1313, (2014).

3 Biemann, K. Contributions of mass spectrometry to peptide and protein structure. *Biomed Environ Mass Spec* **16**, 99-111, (1988).

4 Roepstorff, P. & Fohlman, J. Proposal for a Common Nomenclature for Sequence Ions in Mass Spectra of Peptides. *Biomed Mass Spec* **11**, 601-601, (1984).

5 Bischoff, D. *et al.* The biosynthesis of vancomycin-type glycopeptide antibiotics-a model for oxidative side-chain cross-linking by oxygenases coupled to the action of peptide synthetases. *ChemBioChem* **6**, 267-272, (2005).

6 Tailhades, J. *et al.* A route to diastereomerically pure phenylglycine thioester peptides: crucial intermediates for investigating glycopeptide antibiotic biosynthesis. *Chem Commun* **54**, 2146-2149, (2018).

7 Brieke, C., Peschke, M., Haslinger, K. & Cryle, M. J. Sequential In Vitro Cyclization by Cytochrome P450 Enzymes of Glycopeptide Antibiotic Precursors Bearing the X-Domain from Nonribosomal Peptide Biosynthesis. *Angew Chem Int Ed* **54**, 15715-15719, (2015).

8 Brieke, C., Kratzig, V., Haslinger, K., Winkler, A. & Cryle, M. J. Rapid access to glycopeptide antibiotic precursor peptides coupled with Cytochrome P450-mediated catalysis: towards a biomimetic synthesis of glycopeptide antibiotics. *Org Biomol Chem* **13**, 2012-2021, (2015).

9 MacNeil, D. J. *et al.* Analysis of Streptomyces avermitilis genes required for avermectin biosynthesis utilizing a novel integration vector. *Gene* **111**, 61-68, (1992).

10 Menges, R., Muth, G., Wohlleben, W. & Stegmann, E. The ABC transporter Tba of Amycolatopsis balhimycina is required for efficient export of the glycopeptide antibiotic balhimycin. *Applied Microbiol Biotech* **77**, 125-134, (2007).

11 Bennett, P. M., Grinsted, J. & Richmond, M. H. Transposition of TnA does not generate deletions. *Mol Gen Genetics* **154**, 205-211, (1977).

12 Myronovskyi, M., Welle, E., Fedorenko, V. & Luzhetskyy, A. β-Glucuronidase as a Sensitive and Versatile Reporter in Actinomycetes. *Applied Environ Microbiol* **77**, 5370-5383, (2011).

13 Holm, L. & Rosenström, P. Dali server: conservation mapping in 3D. *Nucleic Acids Res* **38**, W545-W549, (2010).

14 Haslinger, K. & Cryle, M. Structure of OxyAtei: completing our picture of the glycopeptide antibiotic producing Cytochrome P450 cascade. *Febs Lett* **590**, 571-581, (2016).

15 Cryle, M. J., Staaden, J. & Schlichting, I. Structural characterization of CYP165D3, a cytochrome P450 involved in phenolic coupling in teicoplanin biosynthesis. *Arch Biochem Biophys* **507**, 163-173, (2011).

16 Pylypenko, O., Vitali, F., Zerbe, K., Robinson, J. A. & Schlichting, I. Crystal Structure of OxyC, a Cytochrome P450 Implicated in an Oxidative C-C Coupling Reaction during Vancomycin Biosynthesis. *J Biol Chem* **278**, 46727-46733, (2003).

17 Ulrich, V., Brieke, C. & Cryle, M. J. Biochemical and structural characterisation of the second oxidative crosslinking step during the biosynthesis of the glycopeptide antibiotic A47934. *Beilstein J Org Chem* **12**, 2849-2864, (2016).

18 Zerbe, K. *et al.* Crystal Structure of OxyB, a Cytochrome P450 Implicated in an Oxidative Phenol Coupling Reaction during Vancomycin Biosynthesis. *J Biol Chem* **277**, 47476-47485, (2002).

19 Ulrich, V., Peschke, M., Brieke, C. & Cryle, M. J. More than just recruitment: the X-domain influences catalysis of the first phenolic coupling reaction in A47934 biosynthesis by Cytochrome P450 StaH. *Mol BioSys* **12**, 2992-3004, (2016).

20 Brieke, C., Tarnawski, M., Greule, A. & Cryle, M. J. Investigating Cytochrome P450 specificity during glycopeptide antibiotic biosynthesis through a homologue hybridization approach. *J Inorg Biochem* **185**, 43-51, (2018).

21 Xu, L.-H. *et al.* Structural basis for the 4′-hydroxylation of diclofenac by a microbial cytochrome P450 monooxygenase. *Appl Microbiol Biotech* **99**, 3081-3091, (2015).

22 Akimasa, M. *et al.* Substrate Recognition by a Dual-Function P450 Monooxygenase GfsF Involved in FD-891 Biosynthesis. *ChemBioChem* **18**, 2179-2187, (2017).

23 Sugimoto, H. *et al.* Crystal Structure of CYP105A1 (P450SU-1) in Complex with 1α,25-Dihydroxyvitamin D3. *Biochemistry* **47**, 4017-4027, (2008).

24 Haslinger, K., Maximowitsch, E., Brieke, C., Koch, A. & Cryle, M. J. Cytochrome P450 OxyBtei catalyzes the first phenolic coupling step in teicoplanin biosynthesis. *ChemBioChem* **15**, 2719-2728, (2014).

25 G., M. J. *et al.* Structural characterization of CalO2: A putative orsellinic acid P450 oxidase in the calicheamicin biosynthetic pathway. *Proteins Struct Func Bioinf* **74**, 50-60, (2009).

26 Cryle, M. J. & Schlichting, I. Structural insights from a P450 Carrier Protein complex reveal how specificity is achieved in the $P450_{BioI}$ ACP complex. *Proc Nat Acad Sci USA* **105**, 15696-15701, (2008).

27 Uhlmann, S., Süssmuth, R. D. & Cryle, M. J. Cytochrome P450sky Interacts Directly with the Nonribosomal Peptide Synthetase to Generate Three Amino Acid Precursors in Skyllamycin Biosynthesis. *ACS Chem Biol* **8**, 2586-2596, (2013).

28 Haslinger, K. *et al.* The Structure of a Transient Complex of a Nonribosomal Peptide Synthetase and a Cytochrome P450 Monooxygenase. *Angew Chem Int Ed* **53**, 1-6, (2014).

29 Cryle, M. J., Meinhart, A. & Schlichting, I. Structural Characterization of OxyD, a Cytochrome P450 Involved in b-Hydroxytyrosine Formation in Vancomycin Biosynthesis. *J Biol Chem* **285**, 24562-24574, (2010).

30 Haslinger, K., Peschke, M., Brieke, C., Maximowitsch, E. & Cryle, M. J. X-domain of peptide synthetases recruits oxygenases crucial for glycopeptide biosynthesis. *Nature* **521**, 105-109, (2015).

31 Samel, S. A., Schoenafinger, G., Knappe, T. A., Marahiel, M. A. & Essen, L.-O. Structural and Functional Insights into a Peptide Bond-Forming Bidomain from a Nonribosomal Peptide Synthetase. *Structure* **15**, 781-792, (2007).

32 Tarry, M. J., Haque, A. S., Bui, K. H. & Schmeing, T. M. X-Ray Crystallography and Electron Microscopy of Cross- and Multi-Module Nonribosomal Peptide Synthetase Proteins Reveal a Flexible Architecture. *Structure* **25**, 783-793.e784, (2017).

33 Drake, E. J. *et al.* Structures of two distinct conformations of holo-non-ribosomal peptide synthetases. *Nature* **529**, 235, (2016).

34 Bloudoff, K., Alonzo, Diego A. & Schmeing, T. M. Chemical Probes Allow Structural Insight into the Condensation Reaction of Nonribosomal Peptide Synthetases. *Cell Chem Biol* **23**, 331-339, (2016).

35 Bloudoff, K., Rodionov, D. & Schmeing, T. M. Crystal Structures of the First Condensation Domain of CDA Synthetase Suggest Conformational Changes during the Synthetic Cycle of Nonribosomal Peptide Synthetases. *J Mol Biol* **425**, 3137-3150, (2013).

36 Tanovic, A., Samel, S. A., Essen, L.-O. & Marahiel, M. A. Crystal Structure of the Termination Module of a Nonribosomal Peptide Synthetase. *Science* **321**, 659-663, (2008).

37 Krissinel, E. & Henrick, K. Inference of Macromolecular Assemblies from Crystalline State. *J Mol Biol* **372**, 774-797, (2007).

38 Mocibob, M. *et al.* Homologs of aminoacyl-tRNA synthetases acylate carrier proteins and provide a link between ribosomal and nonribosomal peptide synthesis. *Proc Nat Acad Sci USA* **107**, 14585-14590, (2010).

39 Giessen, T. W. *et al.* A Synthetic Adenylation-Domain-Based tRNA-Aminoacylation Catalyst. *Angew Chem Int Ed* **54**, 2492-2496, (2015).

40 Trauger, J. W. & Walsh, C. T. Heterologous expression in *Escherichia coli* of the first module of the nonribosomal peptide synthetase for chloroeremomycin, a vancomycin-type glycopeptide antibiotic. *Proc Nat Acad Sci USA* **97**, 3112-3117, (2000).

41 Pootoolal, J. *et al.* Assembling the glycopeptide antibiotic scaffold: The biosynthesis of from *Streptomyces toyocaensis* NRRL15009. *Proc Nat Acad Sci USA* **99**, 8962-8967, (2002).

42 Naruse, N., Oka, M., Konishi, M. & Oki, T. New antiviral antibiotics, kistamicins A and B. II. Structure determination. *J Antibiot* **46**, 1812-1818, (1993).

43 Yim, G., Thaker, M. N., Koteva, K. & Wright, G. Glycopeptide antibiotic biosynthesis. *J Antibiot* **67**, 31-41, (2014).

44 Al Toma, R. S., Brieke, C., Cryle, M. J. & Suessmuth, R. D. Structural aspects of phenylglycines, their biosynthesis and occurrence in peptide natural products. *Nat Prod Rep* **32**, 1207-1235, (2015).

45 Chiu, H.-T. *et al.* Molecular cloning and sequence analysis of the complestatin biosynthetic gene cluster. *Proc Nat Acad Sci USA* **98**, 8548-8553, (2001).

46 Hadatsch, B. *et al.* The biosynthesis of teicoplanin-type glycopeptide antibiotics: Assignment of P450 mono-oxygenases to side chain cyclizations of glycopeptide A47934. *Chem Biol* **14**, 1078-1089, (2007).

47 Peschke, M., Brieke, C. & Cryle, M. J. F-O-G Ring Formation in Glycopeptide Antibiotic Biosynthesis is Catalysed by OxyE. *Sci Rep* **6**, 35584, (2016).

48 Schrödinger Release 2017-3: MacroModel (version 11.7) (Schrödinger, New York, NY, 2017).

49 Schrödinger Release 2017-3: Maestro (version 11.3) (Schrödinger, New York, NY, 2017).

50 Kaminski, G. A., Friesner, R. A., Tirado-Rives, J. & Jorgensen, W. L. Evaluation and Reparametrization of the OPLS-AA Force Field for Proteins via Comparison with Accurate Quantum Chemical Calculations on Peptides. *J Phys Chem B* **105**, 6474-6487, (2001).

51 Hornak, V. *et al.* Comparison of multiple Amber force fields and development of improved protein backbone parameters. *Proteins Struct Funct Bioinf* **65**, 712-725, (2006).

52 Still, W. C., Tempczyk, A., Hawley, R. C. & Hendrickson, T. Semianalytical treatment of solvation for molecular mechanics and dynamics. *J Am Chem Soc* **112**, 6127-6129, (1990).

53 Zhao, Y. & Truhlar, D. G. The M06 suite of density functionals for main group thermochemistry, thermochemical kinetics, noncovalent interactions, excited states, and transition elements: two new functionals and systematic testing of four M06-class functionals and 12 other functionals. *Theoret Chem Acc* **120**, 215-241, (2008).

54 Gaussian 09, Revision E.01 (Gaussian, Inc., Wallingford, CT, 2013).
