# Supplementary material for: Kistamicin biosynthesis reveals the biosynthetic requirements for production of highly crosslinked glycopeptide antibiotics
Source: Nat Commun. 2019 Jun 13;10:2613. doi: 10.1038/s41467-019-10384-w (PMC6565677; doi:10.1038/s41467-019-10384-w)
Supplement: Supplementary file 2 — Description of Additional Supplementary Files [file 41467_2019_10384_MOESM2_ESM.pdf]

Description of Additional Supplementary Files

File name: Supplementary Data 1

Description: Yield crosslinking [%] of synthesized peptides by OxyA, OxyC or $OxyB_{tei}$
