# Supplementary material for: Kistamicin biosynthesis reveals the biosynthetic requirements for production of highly crosslinked glycopeptide antibiotics
Source: Nat Commun. 2019 Jun 13;10:2613. doi: 10.1038/s41467-019-10384-w (PMC6565677; doi:10.1038/s41467-019-10384-w)
Supplement: Supplementary file 3 — Supplementary Data 1 [file 41467_2019_10384_MOESM3_ESM.docx]

**Supplementary Dataset 1: *In vitro* crosslinking**

**Table 1. *In vitro* cyclisation [%] of synthesised kistamicin peptides**

Yield crosslinking [%] of synthesised peptides by OxyA, OxyC or OxyB_tei_

| **peptide** | **enzyme** | **linear** | **monocyclic** | **bicyclic** | **tricyclic** |
| --- | --- | --- | --- | --- | --- |
| **K1-7** | Control | 100 % | - | - | - |
|  | OxyA | 99.7 % | - | - | - |
|  | OxyC | 94.2 % | 5.8 % | - | - |
|  | OxyA + OxyC | 94.4 % | 5.6 % | - | - |
|  | OxyB_tei_ | 63.9 % | 36.1 % | - | - |
| **K1-3D** | Control | 100 % | - | - | - |
|  | OxyA | 100 % | - | - | - |
|  | OxyC | 96.1 % | 3.9 % | - | - |
|  | OxyB_tei_ | 96.6 % | 3.4 % | - | - |
| **K1-3L** | Control | 100 % | - | - | - |
|  | OxyA | 99.8 % | 0.2 % | - | - |
|  | OxyC | 99.5 % | 0.5 % | - | - |
|  | OxyB_tei_ | 98.4 % | 1.6 % | - | - |
| **K4-7D** | Control | 100 % | - | - | - |
|  | OxyA | 99.4 % | 0.6 % | - | - |
|  | OxyC | 41.4 % | 58.6 % | - | - |
|  | OxyA + OxyC | 40.8 % | 59.2 % | - | - |
|  | OxyB_tei_ | 43.4 % | 56.6 % | - | - |
|  | OxyC + OxyB_tei_ | 43.1 % | 56.9 % | - | - |
|  | OxyA + OxyB_tei_ | 97.2 % | 2.8 % | - | - |
| **K4-7L** | Control | 100 % | - | - | - |
|  | OxyA | 100 % | - | - | - |
|  | OxyC | 80.4 % | 19.6 % | - | - |
|  | OxyA + OxyC | 75 % | 25 % | - | - |
|  | OxyB_tei_ | 73.4 % | 26.6 % | - | - |
| **K3-6** | Control | 100 % | - | - | - |
|  | OxyA | 99.8 % | - | - | - |
|  | OxyC | 20.9 % | 79.1 % | - | - |
|  | OxyA + OxyC | 20.8 % | 79.2 % | - | - |
|  | OxyB_tei_ | 76.7 % | 23.3 % | - | - |
|  | OxyC + OxyB_tei_ | 22.8 % | 77.2 % | - | - |
|  | OxyA + OxyB_tei_ | 76.3 % | 23.7 % | - | - |
| **K3-7** | Control | 100 % | - | - | - |
|  | OxyA | 98.5 % | 1 % | 0.5 % | - |
|  | OxyC | 39.9 % | 52.8 % | 7.3 % | - |
|  | OxyA + OxyC | 40.1 % | 51.7 % | 8.2 % | - |
|  | OxyB_tei_ | 55.8 % | 39.3 % | 4.9 % | - |
|  | OxyC + OxyB_tei_ | 39.9 % | 48.7 % | 11.4 % | - |
|  | OxyA + OxyB_tei_ | 64.8 % | 29.6 % | 5.6 % | - |
| **K1-6** | Control | 100 % | - | - | - |
|  | OxyA | 99.1 % | 0.9 % | - | - |
|  | OxyC | 54.9 % | 45.1 % | - | - |
|  | OxyA + OxyC | 54.0 % | 46.0 % | - | - |
|  | OxyB_tei_ | 48.7 % | 51.3 % | - | - |
|  | OxyC + OxyB_tei_ | 46.6 % | 53.4 % | - | - |
|  | OxyA + OxyB_tei_ | 49.1 % | 50.9 % | - | - |
| **peptide** | **enzyme** | **linear** | **monocyclic** | **bicyclic** | **tricyclic** |
| **K1-4** | Control | 100 % | - | - | - |
|  | OxyA | 99.6 % | 0.4 % | - | - |
|  | OxyC | 53.1 % | 46.9 % | - | - |
|  | OxyA + OxyC | 51.5 % | 48.5 % | - | - |
|  | OxyB_tei_ | 85.8 % | 14.2 % | - | - |
|  | OxyC + OxyB_tei_ | 38.5 % | 61.5 % | - | - |
|  | OxyA + OxyB_tei_ | 86.6 % | 13.4 % | - | - |
| **T1-7** | Control | 100 % | - | - | - |
|  | OxyC | 53 % | 47 % | - | - |
|  | OxyB_tei_ | 29.6 % | 70.4 % | - | - |
|  | OxyC + OxyB_tei_ | 29.5 % | 70.5 % | - | - |
