# Supplementary material for: Kistamicin biosynthesis reveals the biosynthetic requirements for production of highly crosslinked glycopeptide antibiotics
Source: Nat Commun. 2019 Jun 13;10:2613. doi: 10.1038/s41467-019-10384-w (PMC6565677; doi:10.1038/s41467-019-10384-w)
Supplement: Supplementary file 5 — Reporting Summary [file 41467_2019_10384_MOESM5_ESM.pdf]

Corresponding author(s): Cryle

Reporting Summary

Nature Research wishes to improve the reproducibility of the work that we publish. This form provides structure for consistency and transparency in reporting. For further information on Nature Research policies, see Authors & Referees and the Editorial Policy Checklist.

Statistical parameters

When statistical analyses are reported, confirm that the following items are present in the relevant location (e.g. figure legend, table legend, main text, or Methods section).

n/a	Confirmed	
☐	☒	The exact sample size (n) for each experimental group/condition, given as a discrete number and unit of measurement
☐	☒	An indication of whether measurements were taken from distinct samples or whether the same sample was measured repeatedly
☒	☐	The statistical test(s) used AND whether they are one- or two-sided<br>*Only common tests should be described solely by name; describe more complex techniques in the Methods section.*
☒	☐	A description of all covariates tested
☐	☒	A description of any assumptions or corrections, such as tests of normality and adjustment for multiple comparisons
☒	☐	A full description of the statistics including central tendency (e.g. means) or other basic estimates (e.g. regression coefficient) AND variation (e.g. standard deviation) or associated estimates of uncertainty (e.g. confidence intervals)
☒	☐	For null hypothesis testing, the test statistic (e.g. F, t, r) with confidence intervals, effect sizes, degrees of freedom and P value noted<br>*Give P values as exact values whenever suitable.*
☒	☐	For Bayesian analysis, information on the choice of priors and Markov chain Monte Carlo settings
☒	☐	For hierarchical and complex designs, identification of the appropriate level for tests and full reporting of outcomes
☒	☐	Estimates of effect sizes (e.g. Cohen's d, Pearson's r), indicating how they were calculated
☐	☒	Clearly defined error bars<br>*State explicitly what error bars represent (e.g. SD, SE, CI)*

Our web collection on statistics for biologists may be useful.

Software and code

Policy information about availability of computer code

Data collection	MacroModel 11.7, Gaussian 09, Coot, Topsin (Bruker), Lab Solutions (Shimadzu), Unicorn 7 (GE), Spectra Manager™ Suite (Jasco), Skyline
Data analysis	Microsoft Excel, Pymol (Schroedinger), antiSMASH 3.0, Muscle, RaxML, PISA, ClustalOmega, Dali, XDS, Aimless, CCP4, Phenix, Phaser, Phyre, NITPIC, SEDPHAT, GUSSI

For manuscripts utilizing custom algorithms or software that are central to the research but not yet described in published literature, software must be made available to editors/reviewers upon request. We strongly encourage code deposition in a community repository (e.g. GitHub). See the Nature Research guidelines for submitting code & software for further information.

Data

Policy information about availability of data

All manuscripts must include a data availability statement. This statement should provide the following information, where applicable:

- Accession codes, unique identifiers, or web links for publicly available datasets
- A list of figures that have associated raw data
- A description of any restrictions on data availability

The authors declare that all other data supporting the findings of this study are available within the paper and its supplementary information files. The raw data underlying Figures 4 and 7, as well as Supplementary Figures 39-47 are provided as a Source Data files. The raw data underlying Supplementary Figures 20, 22, 24,

48-51 are available upon request. The structure of OxyA-X has been deposited in the Protein Data Bank (PDB) on the 20th of August 2018 with the primary accession code 6M7L.

Field-specific reporting

Please select the best fit for your research. If you are not sure, read the appropriate sections before making your selection.

☒ Life sciences ☐ Behavioural & social sciences ☐ Ecological, evolutionary & environmental sciences

For a reference copy of the document with all sections, see nature.com/authors/policies/ReportingSummary-flat.pdf

Life sciences study design

All studies must disclose on these points even when the disclosure is negative.

Sample size	Enzyme activity was tested against 10 different peptide substrates that were selected based on related works concerning the activity of enzyme homologues as well as the structure of the final compound. The 6 inhibitors chosen were again done so through comparison to literature to disclose the best range to probe the properties of these enzymes. Gene disruption strains were prepared in duplicate.
Data exclusions	No data was excluded
Replication	Turnover experiments were all performed in triplicate together with appropriate controls, as were binding curves for substrate affinity
Randomization	Not possible due to the different physical properties of the peptide substrates involved
Blinding	Not relevant

Reporting for specific materials, systems and methods

Materials & experimental systems

n/a	Involved in the study
☐	☒ Unique biological materials
☒	☐ Antibodies
☒	☐ Eukaryotic cell lines
☒	☐ Palaeontology
☒	☐ Animals and other organisms
☒	☐ Human research participants

Methods

n/a	Involved in the study
☒	☐ ChIP-seq
☒	☐ Flow cytometry
☒	☐ MRI-based neuroimaging

Unique biological materials

Policy information about availability of materials

Obtaining unique materials	All bacterial strains will be made available by contacting the authors
